# Spliceosomal proteins direct RNA methylation to modulate gene expression and silence retrotransposons

**DOI:** 10.1038/s41467-026-75362-5

**Published:** 2026-07-08

**Authors:** Drisya Vijayakumari, Xander Gottfried, Brent Groubert, Jothy Dhakshnamoorthy, Shweta Jain, Martin Zofall, Hernan Diego Folco, Hua Xiao, Anupa T Anil, Thorkell Andresson, David Wheeler, Shiv I. S. Grewal

**Affiliations:** 1 Laboratory of Biochemistry and Molecular Biology, National Cancer Institute, National Institutes of Health, Bethesda, MD USA; 2 Cancer Research Technology Program, Frederick National Laboratory for Cancer Research, Frederick, MD USA; 3 Present Address: The Rockefeller University, 1230 York Avenue, New York, NY USA; 4 Present Address: Department of Molecular Biosciences, Northwestern University, Evanston, IL USA

**Keywords:** Gene silencing, Gene regulation, RNA modification

## Abstract

RNA modifications are fundamental to gene regulation and RNA processing, yet their diversity and transcript specificity remain incompletely defined. Here, using a genetic screen in *S. pombe*, we identify the RNA methyltransferase Tgs1 and Coilin-related proteins as regulators of transcripts harboring inefficiently spliced cryptic introns. These factors associate to form a protein assembly, termed TEaM, which is recruited to cryptic-intron-containing RNAs, including retrotransposon-derived transcripts, by spliceosomal components, and to gametogenic gene transcripts by a YTH-family RNA-binding protein. Upon recruitment, Tgs1 catalyzes trimethylguanosine (TMG) capping, facilitating engagement of the conserved factor Pir2/ARS2, which cooperates with the DROSHA homolog Pac1 and other factors to promote RNA processing and RNAi-mediated silencing. This pathway also targets centromeric repeat RNAs containing cryptic introns, enabling de novo production of siRNAs that specify heterochromatin nucleation. Together, these findings delineate a mechanism in which Tgs1-directed TMG capping, coupled with Pir2/ARS2, specifies RNAi substrates to broadly regulate gene expression and silence retrotransposons.

## Abstract (web summary)

RNA modifications help determine how RNAs are processed and regulated. Here, the authors identify a pathway in fission yeast in which Tgs1-mediated RNA capping recruits RNAi machinery to target cryptic-intron-containing transcripts, promoting their processing and silencing.

## Introduction

The precise and timely regulation of gene expression is essential for the progression of developmental programs and cellular adaptation to changes in environmental or growth conditions. Gene regulation occurs at multiple levels and involves both transcriptional and post-transcriptional mechanisms. At the transcriptional level, chromatin remodeling directed by histone-modifying enzymes plays a key role^1,2^. Post-transcriptionally, factors affecting RNA stability provide another level of control^3,4^. Recent studies have highlighted the pivotal role of RNA modifications as regulators of gene expression^5–8^. These chemical modifications can be dynamically regulated in response to external signals, influencing RNA processing and degradation to rapidly reprogram gene expression^9,10^. Although the importance of RNA modifications and their associated enzymes is increasingly recognized, key questions remain. In particular, how these enzymes target specific RNA transcripts and influence RNA processing remains to be fully investigated.

The fission yeast *Schizosaccharomyces pombe* serves as an excellent model for studying gene regulatory pathways due to its highly conserved RNA processing mechanisms. *S. pombe* retains the core components of the RNA interference (RNAi) machinery, including RNA-dependent RNA polymerase (Rdp1), Dicer (Dcr1), and Argonaute (Ago1), which generate small interfering RNAs (siRNAs) from specific transcripts^1^. These siRNAs guide the Ago1-containing RNA-induced transcriptional silencing (RITS) complex to nascent transcripts, facilitating gene silencing through degradation of target RNAs^11–14^. Additionally, the RITS complex recruits the histone methyltransferase Clr4^15,16^, a homolog of mammalian Suv39h, which methylates histone H3 on lysine 9 (H3K9me) to promote heterochromatin formation^17^. Notably, siRNA production by RNAi requires spliceosomal proteins^18^. Moreover, transcripts targeted by the RNAi machinery often contain inefficiently spliced, or cryptic introns, which accumulate in cells defective in RNA processing^19–22^. These transcripts originate from various loci, including pericentromeric repeats, *Tf2* retrotransposons, regulated genes, and non-coding RNAs (ncRNAs)^19–21^. Mutations that disrupt the splice sites of cryptic introns in centromeric repeats or regulated gene loci abolish siRNA production^19,20,22^. While these findings underscore the importance of cryptic introns as silencing elements, the mechanisms by which these introns and the splicing machinery direct RNAi factor recruitment, and how RNA modifications influence this process, remain to be elucidated.

Another highly conserved RNA processing system, present from *S. pombe* to humans, centers on the YTH family RNA-binding protein Mmi1^23^. Mmi1 recognizes hexameric DSR (determinant of selective removal) motifs embedded in coding and non-coding RNAs^23,24^. Together with Erh1, the ortholog of human enhancer of rudimentary (ERH), Mmi1 forms the Erh1-Mmi1 complex^25^. This complex facilitates RNA degradation by CCR4-NOT^25,26^ and the Mtl1-Red1 core complex (MTREC)^19,27–30^, the latter being functionally analogous to the mammalian poly(A) exosome targeting (PAXT) complex^31^. The MTREC Zn-finger protein Red1 connects the Mtl1 RNA helicase to other factors, including the serine-proline rich protein Pir1^32,33^. Beyond mediating RNA degradation through the nuclear exosome (Rrp6), MTREC-Pir1 also recruits Clr4^Suv39h^ to assemble heterochromatin^19,34–36^. Mmi1 also facilitates silencing by recruiting the cleavage and polyadenylation factor (CPF) complex to promote premature RNA polymerase II (RNAPII) termination at non-canonical sites^37,38^. Importantly, Mmi1 plays a key role in RNAi-mediated silencing of gametogenic genes^39^. Mutations in Mmi1 or RNAi components lead to defective silencing of these genes^23,39,40^, which is associated with chromosomal abnormalities^40^ that are frequently linked to cancer and congenital disorders^41^. Nonetheless, the mechanism by which Mmi1 triggers RNAi-mediated silencing of these developmental genes remains unclear.

Here, we describe a mechanism in which a conserved RNA-modifying factor silences retrotransposons harboring cryptic introns and gametogenic genes. Through a genetic screen, we identified Tgs1, an RNA methyltransferase responsible for trimethylguanosine (TMG) cap formation, together with the human Coilin ortholog Mug174 and a related protein, hereafter referred to as Eas1, as regulators of cryptic intron-containing transcripts. Biochemical analyses suggest that these factors are constituents of a protein assembly, designated TEaM (Tgs1-Eas1-Mug174), that associates with spliceosomal proteins and Mmi1. We find that TEaM is recruited to its targets through two distinct routes. The spliceosomal proteins direct TEaM to transcripts with cryptic introns, whereas Mmi1 targets TEaM to gametogenic genes. TEaM-mediated TMG capping promotes recruitment of the RNAi machinery via Pir2^ARS2^, thereby silencing specific genes and retrotransposons. We further show that depletion of Tgs1 in human cells derepresses endogenous retrotransposons and induces antiviral response pathways.

## Results

### Genetic screen for factors affecting cryptic intron splicing

**Fig. 1:**
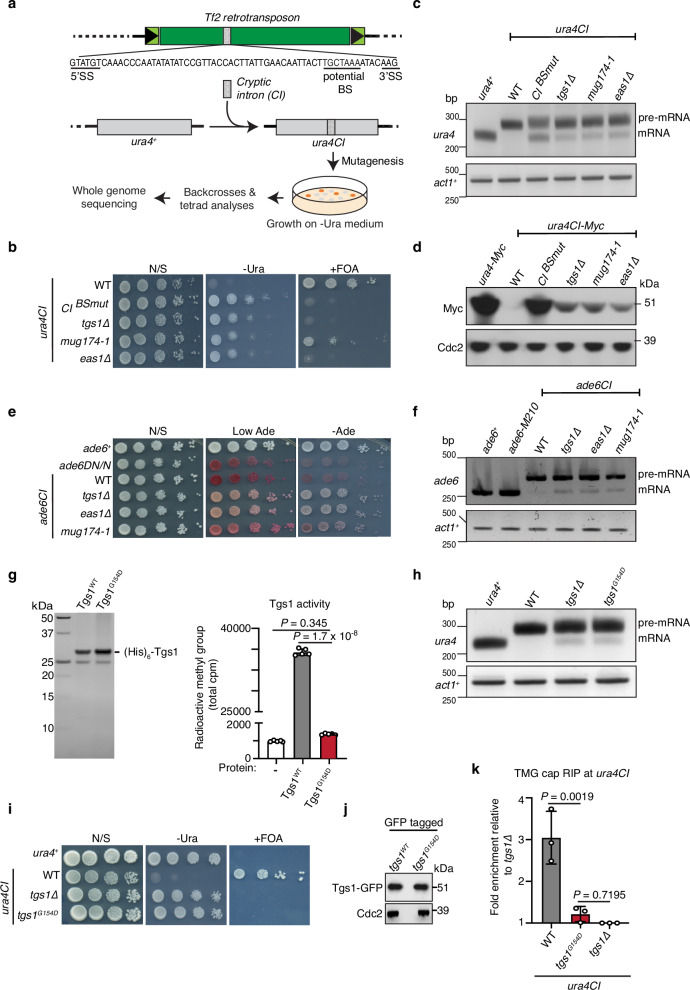
Genetic screen identifies factors that suppress cryptic intron splicing. **a** Schematic of the genetic screen design used to identify mutants affecting cryptic intron splicing. The 62 bp *Tf2* cryptic intron with splice sites as indicated was inserted at the *ura4*^*+*^ locus (*ura4CI*). The *ura4CI* reporter strain was subjected to UV or chemical mutagenesis, and colonies that grew on uracil-deficient medium were selected. Mutants were backcrossed, and mutations were identified by whole-genome sequencing. **b** Serial dilutions of the indicated strains spotted on non-selective (N/S), uracil-deficient (-Ura) and counter-selective 5-fluoroorotic acid media ( + FOA). **c** RT-PCR analysis using primers flanking the cryptic intron in *ura4* (*ura4CI*) to assess its splicing in the indicated strains. *ura4*^*+*^ WT (*ura4*^*+*^) without the *CI* serves as a marker for mRNA. *act1*^*+*^ serves as the control for reverse-transcription and loading. Data are representative of three independent experiments with similar results. **d** Immunoblot analysis of the epitope-tagged Ura4CI-Myc in the indicated strains. Cdc2 serves as the loading control. Data are representative of three independent experiments with similar results. **e** Serial dilutions of the indicated strains were spotted on non-selective (N/S), low adenine (Low Ade) and adenine-free medium (-Ade). Reduced expression of *ade6*^*+*^ produces red colony coloration on low adenine medium. **f** RT-PCR analysis using primers flanking the cryptic intron in the *ade6* reporter to assess splicing in the indicated strains. RT-PCR for *act1*^*+*^ serves as the control. Data are representative of three independent experiments with similar results. **g** Purification profiles (left) and in vitro methyltransferase activity (right) of WT and *tgs1*^*G154D*^ recombinant proteins. Radioactivity transferred from radiolabeled S-adenosyl methionine to m7GTP was measured, with protein-free reactions serving as background controls. The y-axis shows radioactivity in counts per million (cpm). Data are presented as mean ± SD; *n* = 5 independent experiments. *P*-values were determined by one-way ANOVA followed by Dunnett’s multiple comparisons test. **h** RT-PCR using primers flanking the cryptic intron in the *ura4* reporter to assess splicing in the indicated strains. Data are representative of three independent experiments with similar results. **i** Serial dilutions of the indicated strains were spotted on the indicated media to monitor *ura4* reporter expression. **j** Immunoblot showing Tgs1 levels in GFP-tagged WT and *tgs1*^*G154D*^ strains. **k** RT-qPCR analysis of RNA immunoprecipitated (RIP) samples showing enrichment of the TMG cap on the *ura4CI* reporter transcript in indicated strains. Fold enrichment was calculated relative to *tgs1∆*, normalized to 1. Data are presented as mean ± SD; *n* = 3 independent experiments. *P*-values were determined by one-way ANOVA followed by Dunnett’s multiple comparisons test. Source data are provided as a Source Data file.

Cryptic introns, despite having canonical 5’ and 3’ splice sites (Supplementary Fig. 1a), are spliced inefficiently. To identify factors that influence the splicing of these cryptic introns, we performed a genetic screen. We inserted a previously identified cryptic intron (*CI*) from the *Tf2* retrotransposon^19^ into the *ura4* gene, generating the *ura4CI* reporter (Fig. 1a). *CI* insertion disrupts *ura4*, preventing growth on medium lacking uracil (-Ura) unless the cryptic intron is spliced. Following mutagenesis of the *ura4CI* strain, we isolated mutants that grew on -Ura medium and then assessed them for growth on counter-selective medium containing 5-Fluoroorotic Acid (FOA) (Supplementary Fig. 1b). Mutants able to grow on -Ura but not on FOA medium showed increased *ura4CI* splicing, as confirmed by RT-PCR (Supplementary Fig. 1c).

To identify loci responsible for altered *ura4CI* splicing, we backcrossed these mutants and performed whole-genome sequencing. Among the 22 mutants isolated, two contained mutations within the cryptic intron itself (Supplementary Fig. 1d). These *cis*-acting mutant alleles mapped to the putative branch point, which we confirmed using lariat intron sequencing (Supplementary Fig. 2a). In both cases, a *G*-to-*A* substitution at the *TGCTAAA* branch site is predicted to restore proper base pairing with U2 snRNA, thereby enhancing intron splicing (Supplementary Figs. 1b–d, 2a). These data suggest that the *Tf2* cryptic intron contains a suboptimal branch point, resulting in inefficient splicing.

Of the remaining 20 mutants, 18 carried mutations mapping to one of three loci: *tgs1*, *mug174*, and *SPAC18G6.13* (Supplementary Fig. 1d). The other two mapped to genes encoding splicing factors, specifically the U1 snRNP component Luc7 and the branch-site-binding protein Bpb1 (Supplementary Fig. 1d). Among the frequently mutated loci, *tgs1* encodes an RNA methyltransferase that catalyzes 2,2,7-trimethylguanosine capping of RNAs^42^, whereas *mug174* encodes the *S. pombe* homolog of Coilin, a component of Cajal bodies (Supplementary Fig. 2b, c)^43^. *SPAC18G6.13*, which we named Eas1 (Effect Analogous to Tgs1), encodes a protein that shares homology with the amino terminus of Mug174 (Supplementary Fig. 2d). Superimposition of predicted structures shows substantial overlap between the amino-terminal regions of Mug174 and *SPAC18G6.13* (Supplementary Fig. 2e).

To validate these findings, we generated deletion alleles of *tgs1*, *mug174* and *eas1*. Whereas *tgs1∆* and *eas1∆* cells grew comparably to WT cells, *mug174∆* cells exhibited a slow-growth phenotype, as previously reported^43^. We therefore used CRISPR to generate a partial loss-of-function allele (*mug174-1*) carrying the V32M mutation, which maps to the self-interaction domain of Mug174^43^. Similarly, we used CRISPR to recreate the branch site mutation in *ura4CI* (*CI*^*BSmut*^). The *tgs1∆, eas1∆, mug174-1*, and *CI*^*BSmut*^ alleles reproduced the phenotypes of the mutants isolated in the genetic screen (Fig. 1b, c). Increased cryptic intron splicing in these mutants correlated with elevated Ura4 protein levels, as demonstrated by western blot analysis of *Myc* epitope-tagged *ura4CI* (Fig. 1d).

To determine whether *tgs1*, *mug174*, or *eas1* affects cryptic introns inserted at other loci, we assessed their effects on a *Tf2 CI* inserted at *ade6* (*ade6CI*). Cells carrying *tgs1∆*, *eas1∆*, and *mug174-1* alleles showed enhanced *ade6CI* splicing and reporter gene expression (Fig. 1e, f). These results indicate that cryptic introns can be transferred between loci and that Tgs1, Mug174, and Eas1 affect *CI* splicing across different genomic contexts.

### Cryptic intron control requires Tgs1 methyltransferase activity

To determine whether Tgs1 methyltransferase activity is required for modulating cryptic intron splicing, we mutated a conserved residue within its methyltransferase domain (Tgs1^G154D^). In vitro methyltransferase assays using purified recombinant proteins showed that WT Tgs1 exhibits robust activity, as indicated by methyl group transfer from S-adenosyl methionine to the cap analog m7GTP (Fig. 1g). In contrast, Tgs1^G154D^ showed significantly reduced enzymatic activity (Fig. 1g).

We next introduced the *tgs1*^*G154D*^ allele into cells carrying *ura4CI* to assess its impact on cryptic intron splicing. *tgs1*^*G154D*^ mutant cells showed enhanced cryptic intron splicing and, consequently, increased *ura4* expression (Fig. 1h, i). This effect was not due to altered protein expression, as Tgs1 was expressed at comparable levels in *tgs1*^*WT*^ and *tgs1*^*G154D*^ cells (Fig. 1j). To test whether Tgs1 catalyzes TMG capping of *ura4CI* transcripts, we performed RNA immunoprecipitation (RIP) analyses using a TMG-specific antibody. *ura4CI* transcripts were TMG capped in WT cells, and this modification was substantially reduced in both the *tgs1*^*G154D*^ catalytic mutant and *tgs1∆* cells (Fig. 1k). Together, these in vitro and in vivo results indicate that the methyltransferase activity of Tgs1 is crucial for controlling cryptic intron splicing.

### Tgs1, Eas1, and Mug174 associate with each other

**Fig. 2:**
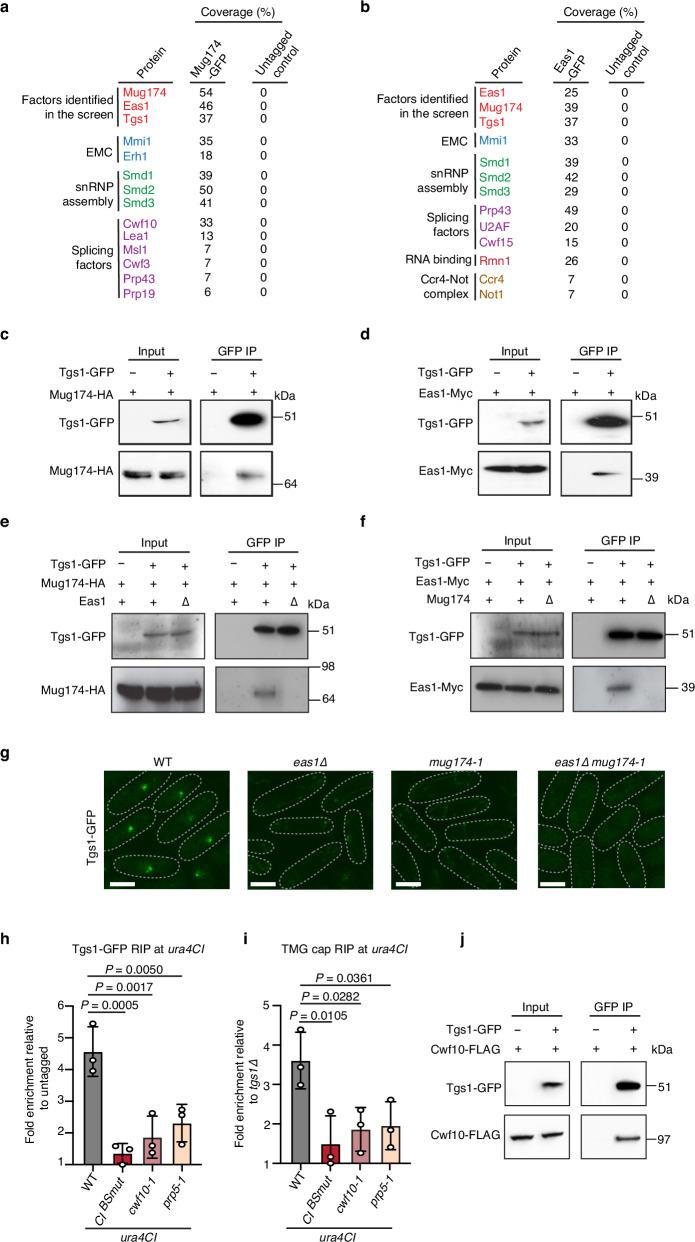
Tgs1, Mug174 and Eas1 interact with each other. **a**,**b** Mass spectrometry analyses of immunopurified Mug174-GFP (**a**) and Eas1-GFP (**b**) fractions. Strains lacking the epitope tag serve as untagged controls. Total peptide coverage for each interacting protein is shown relative to the untagged control. **c**,**d** Immunoblot showing the co-IP of Mug174-HA with Tgs1-GFP (**c**), and Eas1-Myc with Tgs1-GFP (**d**). **e**,**f** Immunoblot showing the co-IP of Mug174-HA with Tgs1-GFP in the presence and absence of Eas1 (**e**), and Eas1-Myc with Tgs1-GFP in the presence and absence of Mug174 (**f**). **g** Representative live-cell imaging of Tgs1-GFP in the indicated strains. 100–150 cells were imaged in each experiment with similar results. Scale bars, 5 µm. **h**,**i** RT-qPCR analysis of RIP samples assessing enrichment of Tgs1 (**h**) or TMG cap (**i**) on *ura4CI* reporter transcript variants in the indicated strains. Enrichments were quantified relative to an untagged control strain or *tgs1∆*, respectively. Data are presented as mean ± SD; *n* = 3 independent experiments. *P*-values were determined by one-way ANOVA followed by Dunnett’s multiple comparisons test. **j** Co-IP of Cwf10-FLAG with Tgs1-GFP, shown alongside the untagged control. **c**,**d**,**e**,**f**,**j** Data are representative of two independent experiments with similar results. Source data are provided as a Source Data file.

To identify interacting partners of the factors uncovered in our screen, we performed immunoaffinity purification using epitope-tagged proteins. Cells expressing tagged Tgs1, Mug174, or Eas1 grew normally and exhibited no detectable changes in *ura4CI* splicing (Supplementary Fig. 3a). Mass spectrometric analysis of Mug174 purifications identified Tgs1, consistent with previous work^43^, as well as Eas1 (Fig. 2a). Reciprocally, Eas1 co-purified with Tgs1 and Mug174 (Fig. 2b). These interactions were validated by co-immunoprecipitation (co-IP) (Fig. 2c, d). To assess interdependence in protein associations, we examined whether loss of Mug174 affects the Tgs1–Eas1 interaction and whether deletion of Eas1 impacts the Tgs1–Mug174 interaction. In both cases, Tgs1 association with its partner was disrupted in the absence of the third component (Fig. 2e, f). However, we do not rule out the possibility that a residual association between Tgs1 and Mug174 is maintained in cells lacking Eas1, and vice versa. Collectively, these findings indicate that Tgs1, Mug174, and Eas1, each recurrently identified in our screen, are constituents of TEaM.

We next examined the interdependence of TEaM components for their subnuclear localization. Both Mug174 and Tgs1 form nuclear condensates reminiscent of mammalian Cajal bodies^43^. Tgs1–GFP nuclear foci were partially affected in *mug174-1* cells, which carry a mutation in the amino-terminal domain of Mug174 required for Tgs1 interaction^43^, and in *eas1Δ* cells (Fig. 2g). Tgs1 foci were nearly abolished in the *eas1Δ mug174-1* double mutant (Fig. 2g). This loss was not attributable to decreased Tgs1 protein levels (Supplementary Fig. 3b). Similarly, Mug174 localization was severely compromised in the *eas1Δ mug174-1* double mutant, whereas each single mutant retained weak foci (Supplementary Fig. 3c). Together with prior evidence for interdependent localization of Tgs1 and Mug174^43^, these results support a model in which a substantial fraction of Tgs1, Mug174, and Eas1 are physically associated.

Mass spectrometry analyses of both Mug174 and Eas1 purifications also identified several additional factors, including Sm proteins (Smd1, Smd2, and Smd3) (Fig. 2a, b and Supplementary Data 1), which are implicated in the processing and maturation of snRNAs and telomerase RNA TER1^44–46^. We also detected multiple core spliceosome components (Fig. 2a, b), suggesting that TEaM functionally interfaces with the splicing machinery. Notably, the YTH domain protein Mmi1, known for its role in silencing gametogenic genes^23^, co-purified with TEaM (Fig. 2a, b). These findings indicate that TEaM associates with other factors that may help direct its methyltransferase activity to a diverse set of RNA substrates.

### Inefficient cryptic intron splicing is linked to Tgs1 recruitment

The finding that Tgs1 catalyzes TMG capping of the *ura4CI* transcript (Fig. 1k) prompted us to investigate whether its recruitment is directly linked to inefficient splicing of the cryptic intron. To address this, we examined Tgs1 binding and TMG capping of the *ura4* transcript containing either WT *CI* or *CI*^*BSmut*^, which harbors a branch-point mutation that enables efficient splicing of the cryptic intron (Fig. 1c). RIP analyses revealed enrichment of Tgs1 and the TMG cap at the *ura4* transcript containing WT *CI* (Fig. 2h, i). In contrast, both Tgs1 binding and TMG capping were markedly reduced at the *ura4CI*^*BSmut*^ transcript (Fig. 2h, i). These results suggest that inefficient cryptic intron splicing is functionally linked to Tgs1 recruitment and modification of this reporter transcript.

We next examined whether the splicing machinery recruits Tgs1 to transcripts harboring cryptic introns. The splicing factor Cwf10, a U5 snRNP subunit that co-purified with TEaM (Fig. 2a), has been implicated in the regulation of cryptic intron–containing RNAs^19,22^. A mutation in *cwf10* (ref. ^18^) markedly reduced Tgs1 binding to the *ura4CI* transcript (Fig. 2h), despite comparable Tgs1 levels in *cwf10-1* mutant and WT cells (Supplementary Fig. 3b). Consistent with this, *cwf10-1* mutants exhibited reduced TMG capping of the *ura4CI* transcript (Fig. 2i). Co-IP experiments further showed that Tgs1 associates with Cwf10 (Fig. 2j), corroborating the IP–mass spectrometry results (Fig. 2a). Similarly, the splicing factor mutant *prp5-1*, encoding a component of the Prp19 complex that also co-purified with TEaM (Supplementary Data 1), displayed reduced Tgs1 binding and TMG capping of *ura4CI* (Fig. 2h, i). Together, these findings indicate that the splicing machinery recruits Tgs1 to inefficiently spliced transcripts, thereby promoting TMG capping.

### Splicing machinery broadly targets Tgs1 RNA methyltransferase

**Fig. 3:**
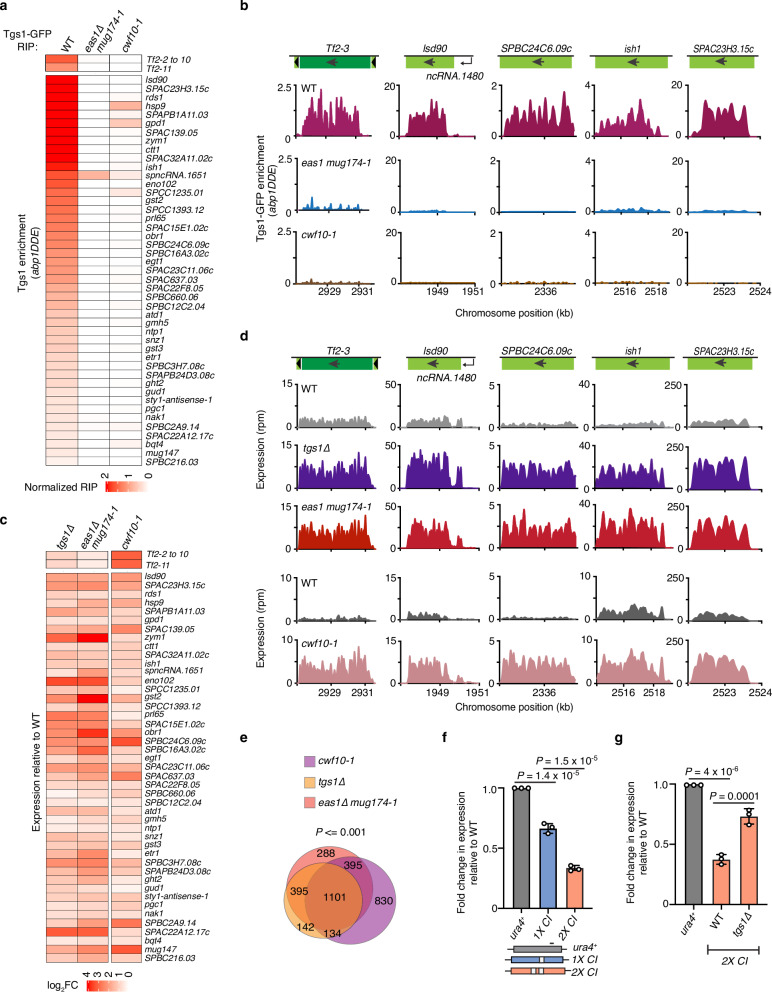
Splicing machinery targets Tgs1 to repress cryptic intron-containing transcripts. **a** Heatmap of Tgs1-GFP enrichment (RIP-seq) on the indicated transcripts in WT, *eas1∆ mug174-1* and *cwf10-1* mutant strains. **b** Tgs1-GFP enrichment profiles (RIP-seq) for representative transcripts in the indicated strains. For (**a**) and (**b**), the *abp1DDE* mutation is included in all genetic backgrounds to enable *Tf2* transcript detection. The data are representative of two independent experiments. **c** Heatmap of RNA-seq showing upregulation of the indicated transcripts in *tgs1∆*, *eas1∆ mug174-1* and *cwf10-1* mutant strains relative to WT. **d** Expression profiles for representative transcripts in *tgs1∆*, *eas1∆ mug174-1* and *cwf10-1* mutant strains compared with the corresponding WT. For (**c**) and (**d**), *cwf10-1* and the corresponding WT were cultured at 30 °C, whereas *tgs1∆*, *eas1∆ mug174-1* and WT were cultured at 18 °C. The data are representative of two independent experiments. For (**a**–**d**), only transcripts that are both associated with Tgs1 by RIP-seq in two independent experiments and upregulated in *tgs1∆* by RNA-seq in two independent experiments are considered direct Tgs1 targets and are presented. **e** An euler diagram showing overlaps of transcripts upregulated (fold change, FC > = 1.5, *P* < = 0.001) in *tgs1∆*, *eas1∆ mug174-1* and *cwf10-1* mutants. FC is calculated relative to the corresponding WT. *P* < = 0.001 indicates significance of overlap, calculated from one-sided distribution of counts derived from random sampling of N-way intersection as described in Methods. The data are representative of two independent experiments. **f** RT-qPCR analysis of *ura4* transcript levels in one (*1X CI*) and two (*2X CI*) cryptic intron-containing *ura4* reporter strains, normalized to WT *ura4*^*+*^ without cryptic introns. Data are presented as mean ± SD; *n* = 3 independent experiments. *P* values were determined by one-way ANOVA followed by Dunnett’s multiple comparisons test. The black solid line in the schematic indicates the primer position used for RT-qPCR. **g** RT-qPCR analysis of *ura4* transcript levels in the *ura4 2X CI* reporter in WT and *tgs1∆* strains, normalized to *ura4*^*+*^ expression from the non-intron-containing *ura4*^*+*^ locus. Data represent mean ± SD; *n* = 3 independent experiments. *P*-values were determined by one-way ANOVA followed by Dunnett’s multiple comparisons test. Source data are provided as a Source Data file.

To determine whether Tgs1 targets additional transcripts, we performed RNA immunoprecipitation followed by sequencing (RIP-seq) using cells expressing Tgs1-GFP. We used strains carrying a mutation in the CENP-B family protein Abp1 (also known as Cbp1), which increases transcription of *Tf*2 elements^47^ and facilitates detection of factors bound to these transcripts. As expected from previous studies linking Tgs1 to the processing of snRNAs and intron-containing telomerase RNA (TER1) precursors, these RNAs were enriched in the Tgs1-immunoprecipitated fraction (Supplementary Fig. 4a, b). *Tf2* retrotransposon transcripts were also prominent targets of Tgs1 (Fig. 3a, b). Notably, Tgs1 is associated with mRNAs from a broad range of genes (Fig. 3a, b). Tgs1 binding required Mug174 and Eas1, indicating that Tgs1 associates with target transcripts as part of the TEaM (Fig. 3a, b; Supplementary Fig. 4a, b). Consistent with this, cells lacking *eas1* or carrying the *mug174-1* mutation showed defects in mature TER1 production, similar to those observed in *tgs1∆* cells (Supplementary Fig. 4c). These analyses expand the known targets of the Tgs1 RNA methyltransferase beyond snRNAs and TER1 to include retrotransposons and a wide range of genic transcripts.

We next investigated whether the splicing machinery recruits Tgs1 to these RNAs. As noted above, *cwf10-1* mutant cells expressed Tgs1 at levels comparable to WT cells (Supplementary Fig. 3b). However, *cwf10-1* resulted in a marked reduction in Tgs1 binding to both *Tf2* retrotransposon and genic transcripts (Fig. 3a, b). Moreover, *cwf10-1* cells showed decreased Tgs1 association with TER1 and snRNAs, although the effect on snRNAs was less pronounced (Supplementary Fig. 4a). These findings underscore the importance of splicing machinery in engaging Tgs1 with a diverse array of transcripts, indicating that this mechanism operates broadly across the genome.

### TEaM represses cryptic intron-containing transcripts

To investigate whether Tgs1 affects the expression of target transcripts, we performed RNA-sequencing (RNA-seq) analysis of *tgs1∆* cells. These analyses revealed upregulation of a diverse array of transcripts (fold change ≥2), including mRNAs, especially those derived from stress-responsive genes, as well as ncRNAs, antisense RNAs, and *Tf2* retrotransposons (Supplementary Fig. 4d). Notably, 45% of the upregulated transcripts in *tgs1∆* mutants, including mRNAs, ncRNAs, and *wtf* elements, contained cryptic introns (Supplementary Fig. 4d, Supplementary Data 2).

Loss of Tgs1 enhanced splicing of cryptic introns in upregulated transcripts, including those within *Tf2* transcripts (Supplementary Fig. 4d). Moreover, annotated introns with suboptimal branch sites were more efficiently spliced in *tgs1∆* cells compared to WT (Supplementary Fig. 4e), and increased levels of transcripts containing these introns were observed (Supplementary Fig. 4d, e). Cells carrying *eas1∆* or *mug174-1* mutant alleles alone showed only modest upregulation of Tgs1 target transcripts (Supplementary Fig. 4d). However, the *eas1∆ mug174-1* double mutant showed pronounced upregulation comparable to that observed in *tgs1∆* cells (Supplementary Fig. 4d). The synthetic enhancement observed in the *mug174-1 eas1Δ* double mutant is consistent with a model in which Mug174 and Eas1 cooperatively support Tgs1-dependent TEaM activity. Under this model, the hypomorphic *mug174-1* allele retains sufficient function to sustain TEaM activity in cells expressing functional Eas1, but not when Eas1 is simultaneously absent. Because the splicing machinery is involved in recruiting TEaM, we next assessed whether it contributes to repression of Tgs1 target RNAs. Comparison of gene expression profiles from *tgs1∆* and *cwf10-1* mutant cells revealed substantial overlap in upregulated loci (Fig. 3c–e). These data support a model in which the splicing machinery facilitates Tgs1 recruitment, thereby enabling repression of a broad set of transcripts, particularly those containing inefficiently spliced cryptic introns.

We also investigated whether the methyltransferase activity of Tgs1 is essential for repressing retrotransposons and genic loci. Tgs1 is required for TMG capping of both *Tf2* transcripts and target mRNAs (Supplementary Fig. 4f). Importantly, cells expressing a methyltransferase-deficient Tgs1 mutant (Tgs1^G154D^) showed increased expression of *Tf2* elements and target mRNAs at levels comparable to those observed in *tgs1Δ* cells (Supplementary Fig. 4g). These results indicate that the catalytic activity of Tgs1, and TMG capping, are required for repression of retrotransposons and genes.

### Cryptic introns are transferable, dosage-dependent gene-repressive elements

We investigated whether cryptic introns alone carry all the information needed for gene repression and whether this repression is Tgs1-dependent. To test this, we used strains containing either one or two cryptic introns in the *ura4* reporter. A single cryptic intron (*1xCI*) reduced *ura4* transcript levels, while two cryptic introns (*2xCI*) caused stronger repression (Fig. 3f). Consistently, deletion of *tgs1* substantially restored expression of the *ura4 2xCI* reporter (Fig. 3g). These results demonstrate that cryptic introns are transferable gene-repressive elements whose effects are dosage-dependent and require Tgs1.

### TEaM promotes gametogenic gene silencing

**Fig. 4:**
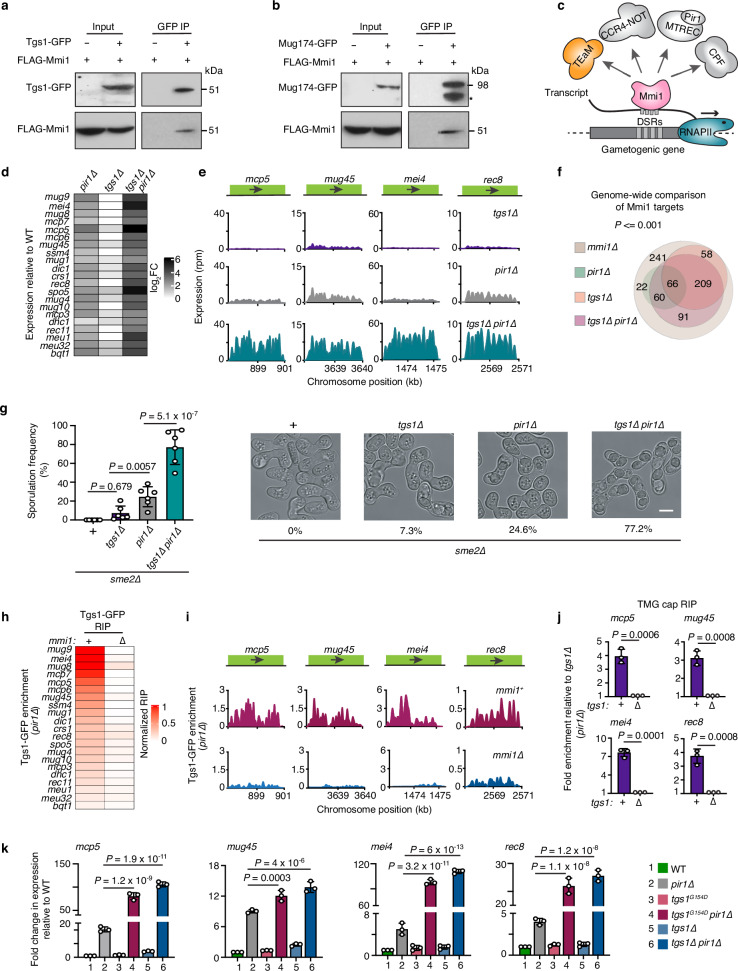
Mmi1 recruits Tgs1 to promote TMG capping and silencing of gametogenic genes. **a**,**b** Co-IP of FLAG-Mmi1 with Tgs1-GFP (**a**) and Mug174-GFP (**b**). Asterisk in (**b**) indicates a non-specific band. Data are representative of two independent experiments with similar results. **c** Schematic model of Mmi1-mediated recruitment of the TEaM, together with other complexes to gametogenic transcripts. **d** Heatmap of gametogenic transcripts in *pir1∆*, *tgs1∆* and *tgs1∆ pir1∆* double mutant strains. The log_2_FC values are calculated relative to WT. **e** Normalized expression profiles of representative gametogenic transcripts in the indicated strains. **f** Euler diagram showing overlap between the Mmi1 regulon and transcripts upregulated in *tgs1∆*, *pir1∆* and *tgs1∆ pir1∆* mutant (fold change, FC > = 2), relative to the corresponding WT. In total, 426 of 753 Mmi1-regulon transcripts are upregulated in the *tgs1∆ pir1∆* double mutant. *P* value for 4-way overlap was calculated from one-sided distribution of counts derived from random sampling of N-way intersection as described in Methods. **g** Sporulation frequency (%) in indicated *sme2∆* zygotes was measured. Only mated cells ( > 100) were included in the analysis. Each data point represents individual results from three independent experiments with two strains per relevant genotype. Bars and error bars represent mean ± SD (left). Representative images of *sme2∆* zygotes in the indicated strain background. Scale bar, 5 µm. (right). **h** Heatmap of Tgs1-GFP enrichment (RIP-seq) on gametogenic transcripts in the presence or absence of Mmi1. All experiments using *mmi1∆* deletion strain also carried a truncation of *mei4* (*mei4∆C*). *pir1∆* is included in both genetic backgrounds to enable detection of gametogenic transcripts. Accordingly, Tgs1 association is detected mainly on gametogenic transcripts derepressed in *pir1∆* and does not encompass the entire Mmi1 regulon. **i** Tgs1 enrichment on representative gametogenic transcripts in the presence or absence of Mmi1. For panels (**d–f**), **h** data are representative of two independent experiments. **j** RT-qPCR analysis showing normalized fold enrichment of TMG-capped transcripts relative to *tgs1∆*. The *pir1∆* background is used to enable detection of gametogenic transcripts. Data are mean ± SD; *n* = 3 independent experiments; *P* values were determined by two-tailed unpaired t test. **k** Fold upregulation of gametogenic transcripts over WT in the indicated strains as determined by RT-qPCR, normalized to *act1*^*+*^. Data are mean ± SD; *n* = 3 independent experiments. *P* values were determined by one-way ANOVA followed by Dunnett’s multiple comparisons test. Source data are provided as a Source Data file.

As described above, TEaM co-purifies with Mmi1 (Fig. 2a, b). Mmi1 binds DSR elements within gametogenic transcripts and recruits multiple effector complexes, including MTREC–Pir1^19^, CCR4–NOT^25,26^, and CPF^37^, which promote premature transcription termination and transcript degradation^1^. We confirmed the interaction between TEaM and Mmi1 by co-IP experiments (Fig. 4a, b).

We next asked whether TEaM contributes to silencing of Mmi1-regulated gametogenic genes (the Mmi1 regulon) (Fig. 4c). Compared to loss of Mmi1, which disrupts multiple silencing effectors, *tgs1∆* resulted in only modest changes in gametogenic transcript levels, as measured by RNA-seq (Supplementary Fig. 5a). Combining *mmi1∆* with *tgs1∆* did not further increase expression of Mmi1 regulon transcripts (Supplementary Fig. 5a, b). We reasoned that the contribution of Tgs1 to gametogenic gene silencing might be masked by other Mmi1-associated effectors. Consistent with this, deletion of *tgs1* in cells lacking Pir1, an adapter linking MTREC to Mmi1, led to a cumulative increase in gametogenic transcripts compared to either single mutant (Fig. 4d, e; Supplementary Fig. 5c). Moreover, transcripts upregulated in the *tgs1∆ pir1∆* double mutant significantly overlapped with those upregulated in *mmi1∆* cells (Fig. 4f). The derepression of gametogenic genes upon loss of Tgs1 is physiologically relevant, as assessed using a functional assay based on rescue of the meiotic arrest observed upon deletion of the *sme2* locus (encoding a noncoding RNA)^23^. *sme2Δ* cells fail to undergo proper meiosis due to an inability to induce meiotic gene expression; however, this defect can be alleviated by disrupting factors involved in gametogenic gene silencing^48^. Deletion of *tgs1* similarly suppressed the meiotic defect in *sme2∆* cells, correlating with derepression of meiotic genes (Fig. 4g). Strikingly, simultaneous loss of Tgs1 and Pir1 rescued meiotic defects in 77.2% of *sme2∆* cells (Fig. 4g). These results demonstrate that Tgs1 contributes to gametogenic gene silencing and plays an important role in preventing untimely activation of the meiotic program.

Cumulative derepression of the Mmi1 regulon was also observed when *tgs1∆* was combined with the deletion of the CPF subunit *ssu72*, although this effect was weaker than that observed in *tgs1∆ pir1∆* cells (Supplementary Fig. 5d, e). In contrast, no additional increase in gametogenic transcripts was detected in the *tgs1∆ ccr4∆* double mutant (Supplementary Fig. 5f), indicating an epistatic relationship between TEaM and CCR4–NOT. Conversely, introducing *ccr4∆* into *pir1∆* cells led to a cumulative accumulation of gametogenic transcripts, closely resembling the phenotype of *tgs1∆ pir1∆* cells (Supplementary Fig. 5f, g). Consistent with this, transcripts upregulated in the *ccr4∆ pir1∆* double mutant significantly overlapped with those upregulated in *mmi1∆* cells, as well as with those upregulated in *tgs1∆ pir1∆* cells (Supplementary Fig. 5h, i). Further supporting the epistatic relationship between TEaM and CCR4–NOT, addition of *tgs1∆* to *ccr4∆ pir1∆* cells did not result in further upregulation; the triple mutant exhibited transcript levels comparable to either *ccr4∆ pir1∆* or *tgs1∆ pir1∆* double mutants (Supplementary Fig. 5f). These analyses demonstrate that Tgs1/TEaM promotes silencing of Mmi1 regulon genes, functioning in parallel with MTREC and CPF, but acting epistatically with CCR4–NOT in this process.

### Mmi1 targets Tgs1 to promote TMG capping

To test if Mmi1 recruits Tgs1 to gametogenic mRNAs, we performed RIP-seq using Tgs1-GFP in *pir1Δ* mutant cells. The *pir1Δ* background provides a sensitized system for detecting factors that bind gametogenic transcripts, as disruption of the MTREC-Pir1 pathway prevents their normal degradation. Our analyses revealed that Tgs1 strongly associates with Mmi1-regulated gametogenic transcripts (Fig. 4h, i), and this association is lost in *mmi1Δ* cells (Fig. 4h, i). This effect is not attributable to reduced Tgs1 levels in *mmi1Δ* cells (Supplementary Fig. 5j). Together with our finding that TEaM co-purifies with Mmi1 (Fig. 4a, b), these results indicate that Mmi1 recruits Tgs1/TEaM to gametogenic transcripts. Targeting of Tgs1 to gametogenic transcripts promotes their TMG capping (Fig. 4j). This activity is functionally important, as combining the catalytic mutant allele *tgs1*^*G154D*^ with *pir1Δ* results in accumulation of gametogenic transcripts to levels comparable to those observed in the *tgs1Δ pir1Δ* double mutant (Fig. 4k). Thus, TMG capping of gametogenic transcripts by Tgs1/TEaM, which is recruited by Mmi1, is crucial for silencing these genes.

Although Mmi1 is required for TEaM association with gametogenic transcripts, *mmi1Δ* cells show increased Tgs1 binding to non-gametogenic transcripts (Supplementary Fig. 5k), suggesting redistribution of Tgs1 upon release from its canonical targets. This binding appears functionally relevant, as these transcripts are upregulated in *tgs1Δ* cells (Supplementary Fig. 5k). Together, these findings raise the possibility that Tgs1 modulates a distinct set of transcripts when Mmi1-dependent control of gametogenic genes is relieved, such as during meiotic induction.

### Tgs1 promotes RNAi-mediated silencing

**Fig. 5:**
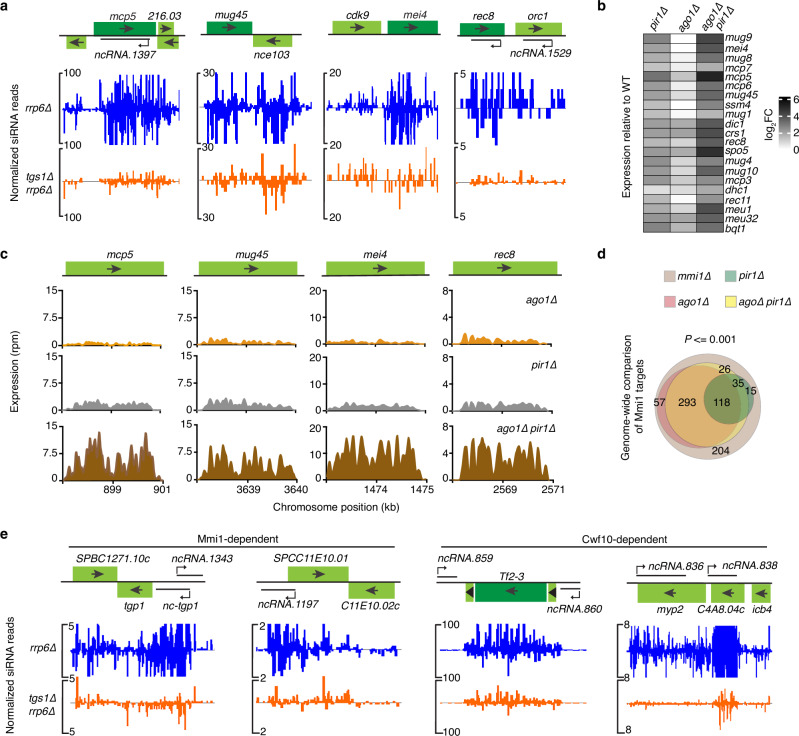
Tgs1 mediates RNAi-dependent silencing of gametogenic gene transcripts. **a** Normalized siRNA reads uniquely mapping to the gametogenic genes *mcp5*, *mug45*, *mei4* and *rec8* in the presence or absence of *tgs1*. *rrp6* deletion is included in both strain backgrounds to enable detection of RNAi-derived siRNAs. **b** Heatmap of RNA-seq data showing gametogenic transcript levels in the indicated strains relative to WT. The log_2_FC values are calculated relative to WT. **c** Normalized RNA-seq expression profiles of representative gametogenic transcripts in the indicated strains. **d** Euler diagram showing overlap between the Mmi1 regulon and transcripts upregulated in *pir1∆*, *ago1∆*, and *ago1∆ pir1∆* double-mutant strains (fold change, FC > = 1.5). In total, 472 of 753 Mmi1-regulon transcripts are upregulated in the *ago1∆ pir1∆* double mutant. *P* value for 4-way overlap was calculated from one-sided distribution of counts derived from random sampling of N-way intersection as described in Methods. **e** Normalized profiles of siRNAs mapping to regions identified as Mmi1-dependent and Cwf10-dependent heterochromatin domains. **a–e** Data are representative of two independent experiments. Source data are provided as a Source Data file.

A key question is how Tgs1 silences target transcripts. Given that Tgs1 shares an epistatic relationship with CCR4-NOT, which is implicated in RNAi^25^, we investigated whether Tgs1 mediates transcript processing via RNAi. To study this, we utilized cells lacking the exosome subunit Rrp6, which can mask the effects of RNAi^14,19^. We detected siRNAs in *rrp6∆* cells mapping to Mmi1 regulon gametogenic genes (Fig. 5a). Strikingly, *tgs1∆* caused a dramatic reduction in siRNAs mapping to these loci (Fig. 5a). Further analyses suggested an epistatic relationship between Tgs1 and Ago1. Loss of Ago1 in *pir1∆* cells led to additive accumulation of Mmi1 regulon transcripts (Fig. 5b–d, Supplementary Fig. 6a), similar to that observed in *tgs1∆ pir1∆* cells (Fig. 4d–f). Moreover, we detected significant overlap in transcripts upregulated in *ago1∆ pir1∆* and *tgs1∆ pir1∆* cells (Supplementary Fig. 6b). Introduction of *tgs1∆* into *ago1∆ pir1∆* cells did not result in further upregulation of gametogenic transcripts compared to either *ago1∆ pir1∆* or *tgs1∆ pir1∆* double mutants (Supplementary Fig. 6c, d). These results implicate Tgs1 in RNAi-mediated silencing of gametogenic genes and suggest that Tgs1/TEaM and MTREC-Pir1, each recruited by Mmi1, act in parallel to silence these genes. While MTREC-Pir1 primarily facilitates exosome-mediated RNA degradation, Tgs1/TEaM enables the RNAi machinery to process gametogenic gene transcripts.

In addition to gametogenic genes, Tgs1 is also required for siRNA production from other transcripts targeted by Mmi1, including *cis*-acting long non-coding RNAs (Fig. 5e and Supplementary Data 3) that regulate the expression of nearby genes^19,20,49,50^. We also examined the requirement for Tgs1 in siRNA production from transcripts containing cryptic introns targeted by spliceosomal proteins. Cells lacking Tgs1 showed a pronounced reduction in siRNAs mapping to loci with cryptic introns, such as *Tf2* retrotransposons and genic loci (Fig. 5e and Supplementary Data 3). The requirement for Tgs1 in processing cryptic intron-containing transcripts into siRNAs was confirmed by northern blot analysis (Supplementary Fig. 6e).

The reduction in siRNA production in *tgs1Δ* cells is not attributable to general RNAi defects, as siRNA production from transcripts lacking Mmi1-binding DSR elements or cryptic introns occurred normally in *tgs1∆* cells (Supplementary Fig. 6f). Collectively, these results demonstrate that recruitment of Tgs1, either through Mmi1 binding to RNAs containing DSR elements or through splicing factors associated with cryptic introns, is crucial for RNAi-mediated processing of target transcripts.

### TEaM mediates heterochromatin nucleation

**Fig. 6:**
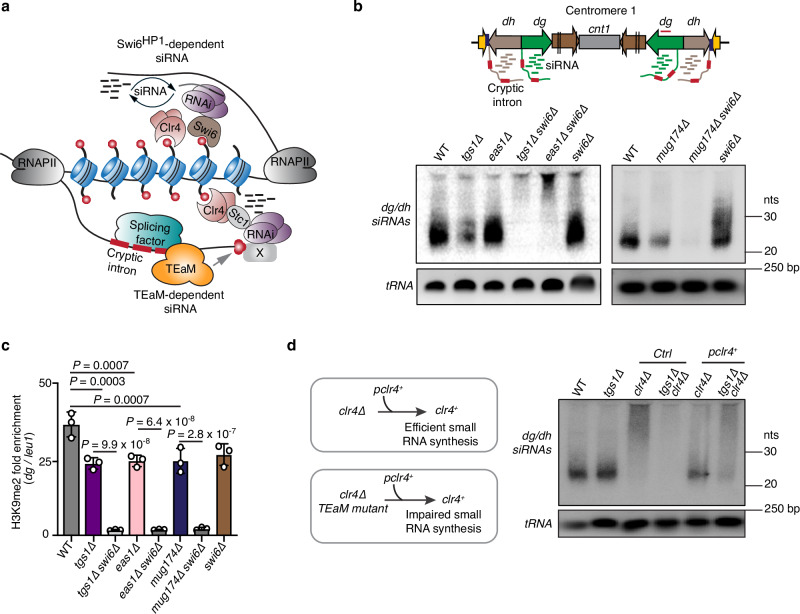
Tgs1 is required to process cryptic intron-containing centromeric transcripts into siRNAs and nucleate heterochromatin. **a** Schematic illustrating cryptic-intron-dependent and Swi6^HP1^-dependent siRNA production pathways. The model depicts cryptic-intron-dependent siRNA production and Swi6-dependent siRNA biogenesis as parallel pathways. **b** Northern blot analysis of centromeric siRNAs in the indicated strains. tRNA levels serve as a loading control and were assessed by agarose gel electrophoresis of 10% of the input RNA from each sample. The schematic shows siRNAs mapping to cryptic intron-containing *dg* and *dh* pericentromeric transcripts. **c** ChIP-qPCR analysis of H3K9me2 enrichment at pericentromeric *dg* repeats. *leu1*^*+*^ was used as the internal qPCR control. Data are mean ± SD; *n* = 3 independent experiments. *P* values were determined by one-way ANOVA followed by Tukey’s multiple comparisons test. The position of the oligo is indicated by the red bar in the schematic in (**b**). **d** Northern blot analysis of centromeric siRNAs in the indicated strains. De novo siRNA production was assessed following the reintroduction of a *clr4*^*+*^-expressing plasmid (*pclr4*^+^) into both *clr4∆* and *tgs1∆ clr4∆* mutant cells. Empty vector (Ctrl) is used as the negative control. tRNA levels serve as a loading control and were assessed by agarose gel electrophoresis of 10% of the input RNA from each sample. **b**,**d** Data are representative of two independent experiments with similar results. Source data are provided as a Source Data file.

RNAi processes centromeric repeat transcripts into siRNAs, which is critical for heterochromatin assembly^1^. Two distinct mechanisms engage the RNAi machinery with centromeric transcripts. In addition to recruitment of RITS and RNA-dependent RNA polymerase by H3K9me and Swi6^HP1^ (refs. ^11,51–53^), the RNAi machinery can be independently targeted through a mechanism involving cryptic introns present in repeat-derived RNAs (Fig. 6a)^19,20,22^. Consistent with these pathways acting in concert, combining *swi6∆* with mutations in either cryptic intron splice sites or the splicing factor Cwf10 results in severe cumulative loss of siRNAs compared to single mutants^22^.

We tested whether TEaM contributes to processing of centromeric transcripts into siRNAs. Cells lacking TEaM components showed reduced siRNA production from pericentromeric repeats (*dg/dh*), subtelomeric regions (*tlh1*), and the silent *mat* region (*cenH*) (Supplementary Fig. 7a–c). Importantly, loss of any TEaM subunit in a *swi6∆* background caused a near-complete loss of *dg/dh*-derived siRNAs. In particular, *eas1∆ swi6∆* and *mug174∆ swi6∆* cells displayed severe defects in siRNA production (Fig. 6b), comparable to those observed in *tgs1∆ swi6∆* mutants (Fig. 6b)^54^. This reduction in siRNA production correlated with impaired heterochromatin assembly at pericentromeric repeats, as indicated by decreased H3K9me2 levels (Fig. 6c).

We next tested whether TEaM is required for de novo siRNA production. Cells lacking the H3K9 methyltransferase Clr4 are defective in siRNA generation, as previously reported (Fig. 6d)^11^. Reintroduction of functional *clr4⁺* restored efficient production of centromeric siRNAs in *clr4∆* cells (Fig. 6d). In contrast, siRNA levels remained severely reduced when *clr4⁺* was reintroduced into *tgs1∆ clr4∆* double mutants (Fig. 6d), indicating that Tgs1 is essential for de novo siRNA generation. Further analyses revealed that the catalytic activity of Tgs1, required for TMG capping of *dg/dh* transcripts (Supplementary Fig. 7d)^54^, is critical for siRNA production. The catalytic mutant *tgs1*^*G154*D^, when combined with *swi6∆*, resulted in a near-complete loss of *dg/*dh siRNAs (Supplementary Fig. 7e), accompanied by increased levels of both forward and reverse centromeric repeat transcripts (Supplementary Fig. 7f).

Together, these findings support a model in which TEaM, recruited via spliceosomal components, promotes TMG capping of cryptic intron-containing centromeric transcripts. This modification facilitates engagement of the RNAi machinery, enabling initial siRNA production and promoting heterochromatin nucleation.

### Tgs1 mediates Pir2^ARS2^ recruitment

Our findings prompted us to investigate how Tgs1 promotes RNAi-mediated silencing of target transcripts. The conserved protein Pir2 (ARS2 in mammals, SERRATE in plants)^55,56^ has been implicated in RNAi-mediated processing of transcripts containing cryptic introns^20^. Pir2^ARS2^ co-purifies with components of the nuclear cap-binding complex (CBC) that recognizes the 7-methylguanosine (7mG) cap, and also interacts with RNA elimination factors targeting gametogenic transcripts^20,27,28^.

**Fig. 7:**
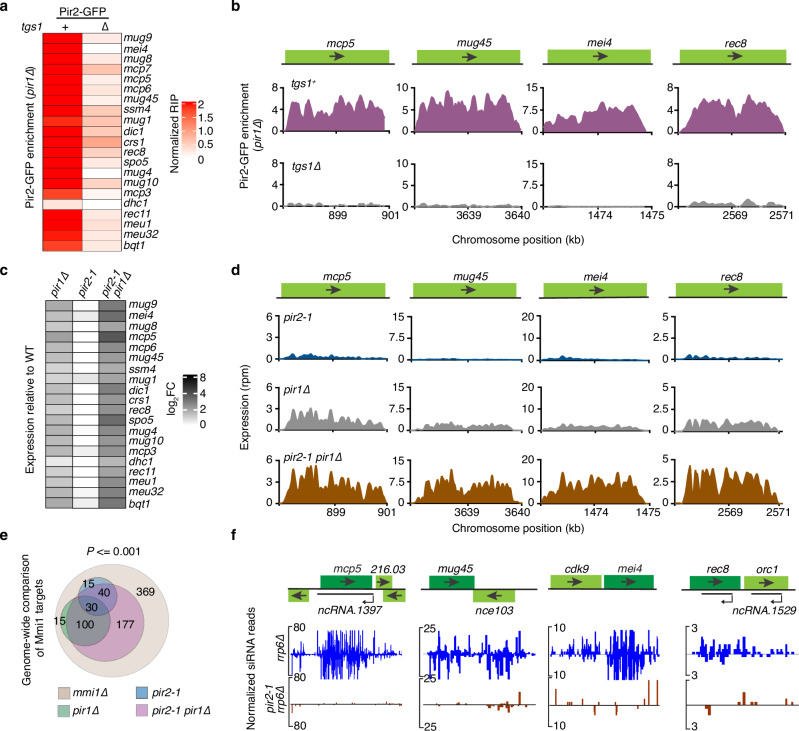
Tgs1 mediates Pir2 recruitment to promote RNAi-mediated silencing of gametogenic genes. **a** Heatmap of Pir2 enrichment (RIP-seq) on gametogenic transcripts in the presence or absence of Tgs1. Enrichment is shown after subtraction of the untagged control. **b** Pir2 enrichment profiles (RIP-seq) on representative gametogenic transcripts in the presence or absence of Tgs1. **c** Heatmap of gametogenic transcript levels in the indicated mutant strains relative to WT. **d** Normalized RNA-seq expression profiles of gametogenic transcripts in the indicated strains. **e** Euler diagram showing overlap between the Mmi1 regulon and transcripts upregulated in *pir1∆, pir2-1* and *pir2-1 pir1∆* double-mutant strains (fold change, FC > = 1.5). In total, 347 of 753 Mmi1-regulon transcripts are upregulated in the *pir2-1 pir1∆* double mutant. *P* value for 4-way overlap was calculated from one-sided distribution of counts derived from random sampling of N-way intersection as described in Methods. **a–e** Data are representative of two independent experiments. **f** Normalized siRNA profiles showing reads uniquely mapping to gametogenic transcripts in *rrp6∆* and *pir2-1 rrp6∆* mutant cells. Source data are provided as a Source Data file.

To determine whether TMG capping by Tgs1 facilitates Pir2^ARS2^ recruitment to gametogenic transcripts, we performed RIP-seq analyses of GFP-tagged Pir2^ARS2^ (Pir2-GFP) in the *pir1∆* background, which allows easier detection of gametogenic transcripts. Pir2^ARS2^ preferentially associated with gametogenic transcripts silenced by Tgs1 (Fig. 7a, b). Strikingly, *tgs1∆* reduced Pir2^ARS2^ binding to gametogenic RNAs (Fig. 7a, b). This change was not due to reduced Pir2^ARS2^ levels in *tgs1∆* cells (Supplementary Fig. 8a). Thus, Tgs1 RNA methyltransferase, which mediates TMG capping, is required for recruitment of Pir2^ARS2^ to gametogenic transcripts.

Functional analyses further showed that Pir2^ARS2^ acts in the same pathway as Tgs1 to silence gametogenic genes. A hypomorphic *pir2* mutant allele (*pir2-1)* alone did not cause derepression (Fig. 7c), but the *pir2-1 pir1Δ* double mutant exhibited increased accumulation of gametogenic transcripts (Fig. 7c, d; Supplementary Fig. 8b). A substantial fraction of Mmi1 regulon genes was upregulated in these cells (Fig. 7e), with significant overlap between transcripts upregulated in *pir2-1 pir1Δ* and *tgs1Δ pir1Δ* mutants (Supplementary Fig. 8c). The *pir2-1 tgs1Δ pir1Δ* triple mutant did not show further increases, consistent with an epistatic relationship between Pir2 and Tgs1 (Supplementary Fig. 8d, e). We next asked whether *pir2-1* affects RNAi-mediated processing of gametogenic transcripts. Introduction of *pir2-1* into *rrp6Δ* cells led to loss of siRNAs mapping to gametogenic genes, similar to *tgs1Δ rrp6Δ* cells (Fig. 7f). Together, these data indicate that Tgs1 promotes recruitment of Pir2^ARS2^ to gametogenic transcripts, enabling RNAi-mediated processing and preventing inappropriate gene expression.

**Fig. 8:**
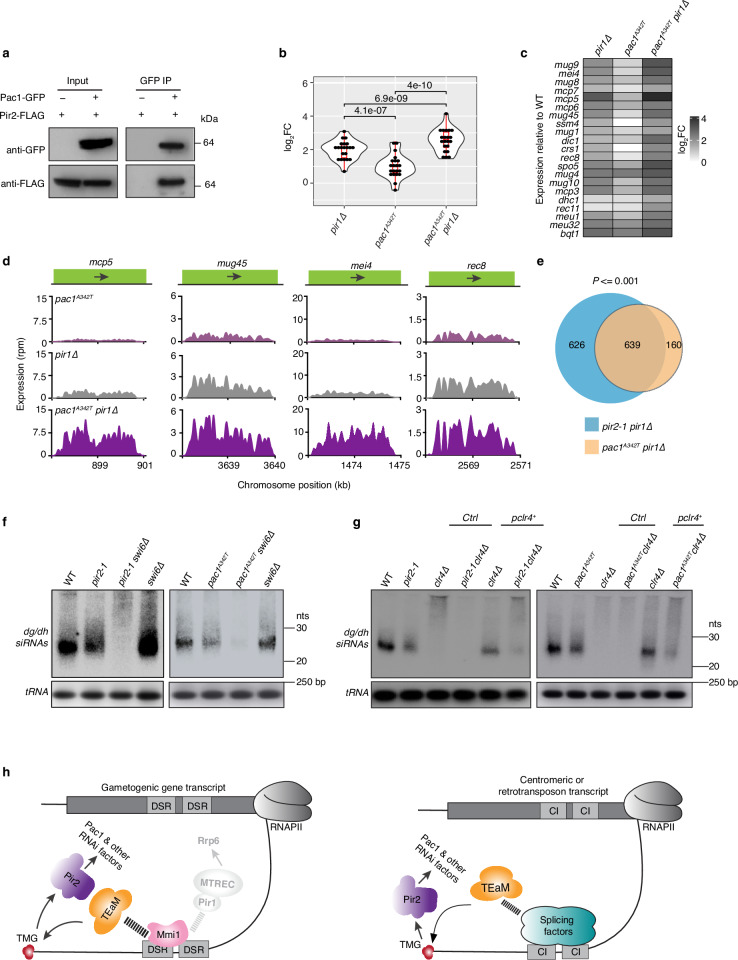
Pir2^ARS2^ acts with Pac1^DROSHA^ to promote gametogenic gene silencing and initiate centromeric siRNA production. **a** Immunoblot showing the co-IP of Pac1-GFP with Pir2-FLAG. Data are representative of two independent experiments with similar results. **b** Violin plots showing the log2FC expression of gametogenic genes (*n* = 21) in the indicated strains normalized to WT. The box-plot in red gives the inter-quartile ranges (25–75th percentile) with the median marked by a horizontal line in the middle. *P* values are estimated using paired 2-tailed t-tests. Data are representative of three biological replicates of RNA-seq experiments. **c** Heatmaps of gametogenic transcripts in the indicated strains. The log_2_FC values are calculated relative to WT. **d** Normalized expression profiles of representative gametogenic transcripts in *pac1*^*A342T*^, *pir1∆* and *pac1*^*A342T*^ *pir1∆* double-mutant strains. **e** Euler-diagram showing overlap in total upregulated transcripts (fold change >=2) in *pir2-1 pir1∆* and *pac1*^*A342T*^ *pir1∆* cells. *P* < = 0.001 indicates significance of overlap calculated from one-sided distribution of counts derived from random sampling of N-way intersection as described in Methods. **f** Northern blot analysis of centromeric siRNAs in the indicated strains. tRNA levels serve as a loading control and were assessed by agarose gel electrophoresis of 10% of the input RNA from each sample. **g** Northern blot analysis of centromeric siRNAs in the indicated strains. De novo siRNA production was assessed following the reintroduction of a *clr4*^*+*^-expressing plasmid (*pclr4*^+^) into both *clr4∆*, *pir2-1 clr4∆* and *pac1*^*A342T*^ *clr4∆* mutant cells. Empty vector (Ctrl) is used as the negative control. tRNA levels serve as a loading control and were assessed by agarose gel electrophoresis of 10% of the input RNA from each sample. **f**,**g** Data are representative of two independent experiments with similar results. **h** Schematic model illustrating two pathways for Tgs1 recruitment: Mmi1-mediated recruitment to gametogenic transcripts (left) and recruitment via spliceosomal proteins to cryptic intron-containing transcripts (right). Trimethylguanosine (TMG) capping of target RNAs by Tgs1 promotes their subsequent association with Pir2, which interacts with RNAi factors, including the RNA-dependent RNA polymerase^20^, and Pac1. (this study), ultimately leading to RNAi-dependent silencing of these transcripts. Source data are provided as a Source Data file.

In metazoans, ARS2 facilitates processing of primary miRNAs by DROSHA, a key step in microRNA biogenesis^57,58^. This prompted us to examine whether the *S. pombe* DROSHA ortholog, Pac1, associates with Pir2^ARS2^. Indeed, Pac1 co-immunoprecipitated with Pir2^ARS2^ (Fig. 8a). Furthermore, combining a partial loss-of-function *pac1* allele (*pac1*^*A342T*^) with *pir1Δ* resulted in enhanced derepression of gametogenic transcripts compared to single mutants (Fig. 8b–d). The upregulated transcripts in *pac1*^*A342T*^ *pir1Δ* cells significantly overlapped with those in *pir2-1 pir1Δ* (Fig. 8e) and *tgs1Δ pir1Δ* mutants (Supplementary Fig. 8f). Importantly, like Tgs1 and Pir2^20^, we find Pac1^DROSHA^ to be required for repressing retrotransposon levels (Supplementary Fig. 9a). These findings suggest that Pir2^ARS2^ likely collaborates with Pac1^DROSHA^ to silence gametogenic genes and retrotransposons.

### Pir2^ARS2^ mimics Tgs1 to nucleate heterochromatin

To determine whether Pir2^ARS2^ and its interacting ribonuclease Pac1^DROSHA^ are required for centromeric siRNA production and heterochromatin nucleation, similar to Tgs1, we analyzed the effects of the *pir2-1* and *pac1*^*A342T*^ mutations individually and in combination with *swi6Δ*. The *pir2-1* and *pac1*^*A342T*^ single mutants showed only a modest reduction in *dg/dh* repeat–derived siRNAs (Fig. 8f). In contrast, the *pir2-1 swi6Δ* and *pac1*^*A342T*^ *swi6Δ* double mutants exhibited severe defects in centromeric siRNA production (Fig. 8f). These results indicate that Pir2 and Pac1, like Tgs1, are required for the generation of Swi6^HP1^-independent siRNAs that drive heterochromatin nucleation.

Factors involved in Swi6^HP1^-independent siRNA production are thought to be critical for initiating the primary cascade of RNAi-mediated heterochromatin assembly. Once nucleated, heterochromatin marks, such as H3K9me and Swi6^HP1^ further promote the targeting of the RNAi machinery, thereby amplifying siRNA production and reinforcing heterochromatin formation^11,51–53^. Consistent with this model, *pir2-1* and *pac1*^*A342T*^ cells maintained pericentromeric heterochromatin, as evidenced by H3K9me2 levels comparable to WT (Supplementary Fig. 9b, c). To test whether Pir2 and Pac1 are required for de novo siRNA production and heterochromatin nucleation, we used *pir2-1 clr4Δ* and *pac1*^*A342T*^ *clr4Δ* double mutants. Upon reintroduction of a plasmid expressing *clr4*^*+*^, siRNAs and H3K9me2 at pericentromeric regions were efficiently restored in the control *clr4Δ* single mutant but not in the *pir2-1 clr4Δ* or *pac1*^*A342T*^ *clr4Δ* double mutants (Fig. 8g; Supplementary Fig. 9b, c). The low levels of siRNAs and H3K9me2 detected in the double mutants likely reflect residual function of the hypomorphic *pir2-1* and *pac1*^*A342T*^ alleles. Together, these findings identify Pir2 and Pac1 as key factors required to initiate the early steps of siRNA production and heterochromatin nucleation at centromeric repeats.

Collectively, our work identifies a function for the conserved RNA methyltransferase Tgs1, which is targeted by the splicing machinery to retrotransposons, centromeric repeats and stress-responsive genes containing cryptic introns. Tgs1 also regulates the expression of gametogenic genes targeted by the YTH family protein Mmi1. This regulation occurs through a mechanism involving Pir2^ARS2^, which is required for RNAi-mediated processing of target transcripts (Fig. 8h).

### TGS1 represses endogenous retrotransposons in human cells

To investigate the function of TGS1 in human cells, we performed siRNA-mediated knockdown in BJ fibroblasts. In cells with validated TGS1 knockdown (Supplementary Fig. 10a, b), we observed increased expression of genes associated with antiviral response pathways (Supplementary Fig. 10b). These included components of the RIG-I (retinoic acid–inducible gene I) and OAS (oligoadenylate synthetase) pathways, which encode sensors of dsRNA^59^. RIG-I recognizes viral RNA intermediates and RNA derived from retrotransposons, triggering an interferon signaling cascade^60^. Similarly, the OAS pathway also restricts endogenous retrotransposons by promoting degradation of RNA derived from these elements^61^. Consistent with activation of RIG-I and OAS pathway genes, TGS1 knockdown led to upregulation of interferon and innate immune response genes (Supplementary Fig. 10b–d). This upregulation of antiviral response pathways prompted us to assess whether retroelements are derepressed under TGS1 knockdown conditions. We detected increased expression of several families of repeat elements, approximately 50% of which correspond to retrotransposons (Supplementary Fig. 10e). In addition, a class of DNA repeat elements (MERs) and satellite repeats were also derepressed in TGS1 knockdown cells (Supplementary Fig. 10e, f). Collectively, these results suggest that the role of TGS1 in silencing retroelements is likely evolutionarily conserved.

## Discussion

RNA processing factors, including RNAi machinery, play a crucial role in preventing untimely gene expression and in silencing repetitive DNA elements, such as retrotransposons. However, the mechanisms by which specific transcripts are preferentially targeted remain poorly understood. Our study uncovers a mechanism in which spliceosomal proteins engaged by cryptic introns, or YTH family proteins, recruit the Tgs1 RNA methyltransferase, which catalyzes TMG capping of target transcripts, thereby marking them as preferred substrates for the RNAi machinery.

Our data support the existence of a Tgs1-containing protein assembly, termed TEaM, which also comprises Mug174 and Eas1. This is supported by the co-purification of these factors and by the observation that the disruption of any single component destabilizes the association among the others. Functionally, all three components similarly contribute to de novo centromeric siRNA production and influence cryptic intron splicing. Indeed, our genetic screen for increased splicing of a cryptic intron identified multiple alleles in Tgs1, Mug174, and Eas1, including mutations mapping to the amino-terminal region of Mug174 that is required for its association with Tgs1^43^. Together, these findings support a model in which physical association among TEaM components is functionally important. However, unlike *tgs1*∆ and *eas1∆* mutants, cells lacking Mug174 display growth defects^43^, and Mug174 and Eas1 appear partially redundant in certain contexts. These differences suggest that, in addition to their shared role within TEaM, individual components likely perform distinct functions outside this assembly.

We further show that inefficiently spliced cryptic introns, which are widespread across eukaryotic genomes^19,62^, can act as transferable regulatory elements that repress gene expression. Our finding that 45% of transcripts upregulated in *tgs1∆* cells contain cryptic introns implies a broader role of Tgs1 in cryptic intron-mediated gene regulation. Introducing a cryptic intron with a weak branch point into a reporter gene is sufficient to trigger Tgs1- and TMG-mediated gene repression, whereas restoring proper splicing reverses this effect. These findings indicate that prolonged spliceosome association with cryptic introns promotes Tgs1 recruitment, a pathway that may have evolved to recognize and silence retrotransposons lacking host-specific splice signals^63^ before being adapted more broadly. As mentioned above, mutations in TEaM components not only affect gene expression but also increase cryptic intron splicing. This is unexpected, given that Tgs1, Mug174, and Eas1, which are important for snRNA processing^64^, are required for the splicing of certain introns^43,65,66^. Suppression of cryptic intron splicing requires Tgs1 catalytic activity and TMG capping, which may inhibit splicing by blocking factors that bind the monomethyl cap or by altering the balance between splicing and RNA degradation. Thus, increased cryptic intron splicing in TEaM mutants may result from reduced RNA degradation, providing more opportunity for splicing. Collectively, these analyses establish that recruitment of Tgs1 by spliceosomal proteins is broadly utilized to control expression of diverse loci, including genes and retrotransposons.

In addition to recruitment by spliceosomal proteins, we found that TEaM is recruited by Mmi1, a member of the YTH family of RNA-binding proteins involved in diverse cellular processes^67^. Mmi1 directs TEaM to developmentally regulated gametogenic genes that are active during meiosis but repressed during mitosis. Unlike mammalian YTH proteins, which typically recognize N6-methyladenosine (m6A)–modified transcripts^6,8^, Mmi1 binds directly to a hexanucleotide RNA sequence motif known as DSR^23,48^. DSR elements and cryptic introns thus represent alternative pathways for TEaM recruitment. Given that YTH domains are also found in splicing factors^67^, the DSR–Mmi1 system may have evolved from intronic elements and splicing machinery to enforce gametogenic gene silencing. Once recruited, TEaM/Tgs1 acts as a silencing effector alongside other Mmi1-associated complexes, such as MTREC–Pir1, which mediate exosomal RNA degradation. This silencing function depends on TMG capping, as cells lacking Tgs1 catalytic activity show aberrant expression of Mmi1 regulon genes, particularly when MTREC–Pir1–mediated decay is impaired. Together, these findings reveal a connection between TMG modification and developmental gene regulation, masked by functional redundancy among Mmi1-associated silencing effectors.

Our study broadens the regulatory scope of TMG modification and defines how RNAi identifies target RNAs. Prior work demonstrated that stalled spliceosomes recruit RNA-dependent RNA polymerase and Argonaute to process transposon-derived transcripts^68^; here, we identify a distinct pathway in which specific spliceosomal proteins promote TMG capping to facilitate RNAi recognition. Consistent with this model, both Cwf10 and Prp5, which we show are required for Tgs1 recruitment and TMG capping of transcripts containing cryptic introns, have previously been implicated in siRNA production and RNAi-dependent heterochromatin formation at centromeric repeats^18^. Importantly, this function appears to be restricted to a subset of splicing factors rather than representing a general property of the spliceosome.

Our findings also suggest that this mechanism is evolutionarily conserved. In human cells, TGS1 depletion results in the accumulation of retrotransposon transcripts and activation of antiviral responses, raising the possibility that TGS1 cooperates with RNA-processing factors involved in silencing repetitive elements and gametogenic genes, such as ERH^69^. Unlike in *S. pombe*, however, TGS1 knockdown did not induce gametogenic gene expression in BJ fibroblasts, likely reflecting the low basal expression of these genes in this cell type. Future studies in appropriate developmental and disease models will be required to determine whether TGS1 similarly suppresses inappropriate activation of gametogenic genes in mammals, including cancer-testis antigens^41^.

In plants, Cajal bodies are thought to concentrate RNAi components to promote RNA-directed DNA methylation^70,71^. Because Tgs1 associates with Mug174, a Cajal body-like condensate component (this study)^43^, such compartments may enrich this RNA methyltransferase activity to facilitate efficient TMG capping and thereby designate transcripts as preferred RNAi substrates. Given that introns and spliceosomal factors contribute to miRNA biogenesis^72–74^, a similar mechanism may help recruit ARS2 to enhance miRNA maturation. In contrast, canonical Tgs1 targets, such as snRNAs and TER1, are protected from RNAi, likely through binding of Sm or Lsm ring complexes to U-rich sites^45,75^, which may preclude their entry into RNAi pathways. Together, these findings support a model in which Tgs1-mediated TMG capping, coordinated by specific spliceosomal factors, helps discriminate RNA substrates for RNAi. More broadly, this model provides a framework for future studies investigating the interplay between splicing, TMG capping, and RNA silencing pathways in genome defense, including potential links to the piRNA pathway^76–78^.

## Methods

### Strains, media, and growth conditions

The *Schizosaccharomyces pombe* strains used in this study are listed in Supplementary Data 4. Gene deletions and epitope-tagged strains were generated using PCR-based module integration and were confirmed by PCR and sequencing. Newly generated strains and plasmids are available from the corresponding author upon request. Standard media, culture methods, and genetic manipulation techniques were employed throughout. Unless otherwise specified, the majority of experiments were performed using logarithmically growing cells cultured at 30 °C in rich yeast extract with adenine (YEA) medium. For experiments involving temperature-sensitive alleles, cells were initially grown at 26 °C and subsequently shifted to 36 °C for 5 h. For RNA-seq and RIP-seq analyses of gametogenic genes, strains were cultured at 18 °C in rich YEA medium. Epitope tagging and gene deletion were performed using PCR module-based techniques. Genetic crosses and tetrad dissections were performed to generate strains with the desired allele combinations. Experiments using the *mmi1Δ* deletion strain also included a truncation of *mei4* (*mei4ΔC*). Sporulation frequency of *sme2∆* zygotes was determined following growth in SPAS medium at 30 °C for 2–3 days. Unmated cells were excluded from the analysis. Measurements were obtained from three independent biological experiments, each including two strains per relevant genotype.

### Cell culture and siRNA transfection

BJ human fibroblast cells (ATCC CRL-2522) were cultured in Eagle’s Minimum Essential Medium (Sigma-Aldrich, M2279) supplemented with 10% fetal bovine serum (FBS; Gibco, A5256801) and 2 mM L-glutamine (Gibco, 25030149) at 37 °C in a humidified atmosphere containing 5% CO₂.

siRNA transfections were performed at 40–50% confluency using Opti-MEM Reduced Serum Medium (Gibco, 31985070). siRNAs were resuspended and complexed with DharmaFECT 1 Transfection Reagent (Dharmacon, T-2002-02) according to the manufacturer’s instructions. A non-targeting control siRNA (Dharmacon ON-TARGETplus Non-targeting Control Pool, D-001810-10-05) and siRNA targeting human *TGS1* (Dharmacon ON-TARGETplus SMARTpool, L-017151-00-0050) were used.

### Genetic screen to identify factors involved in suppression of cryptic intron splicing

Genetic screens were performed using a wild-type heterothallic strain (*mat1M-smt0*) harboring the *ura4CI* reporter at the endogenous *ura4* locus. For chemical mutagenesis, exponentially growing wild-type reporter strains were treated with N-methyl-N’-nitro-N-nitrosoguanidine (MNNG) at a concentration of 0.5 mg/mL for 60 min at room temperature and plated on rich medium. Colonies formed on rich medium were replica-plated onto uracil-deficient minimal medium and counter-selective medium containing 5-fluoroorotic acid (FOA). For UV mutagenesis, exponentially growing cells were plated on rich medium and irradiated with UV light at 150 J/m² using a UV crosslinker (Fisher Scientific). After growth for 2 days at 30 °C, colonies were replica-plated onto uracil-deficient medium and FOA-containing medium. Colonies exhibiting reduced growth on FOA-containing medium were selected as candidate mutants. These candidates were backcrossed twice with the non-mutagenized parental strain. Genomic DNA was isolated from four wild-type and four mutant segregants from the final backcross. Sequencing libraries were prepared using the Illumina Nextera XT DNA Library Preparation Kit (Illumina, 15032354) and sequenced on an Illumina NextSeq 500 platform (see below for details on analysis and identification of mutations).

### RNA Isolation and RT-qPCR

Total RNA was isolated from 5 OD_600_ units of cells harvested at mid-log phase (OD_600_ = 0.4–0.5) using the MasterPure Yeast RNA Purification Kit (Biosearch Technologies, MPY03100) according to the manufacturer’s instructions. For BJ fibroblasts (CRL-2522), RNA extraction was performed using TRIzol reagent followed by purification using the Direct-zol RNA Miniprep Kit (Zymo Research, R2053-A) according to the manufacturer’s instructions. cDNA synthesis was performed using DNase-treated RNA and SuperScript III Reverse Transcriptase (Thermo Fisher Scientific, 18080-044) with a mixture of oligo(dT) (Thermo Fisher Scientific, 18418020) and random hexamer primers (Thermo Fisher Scientific, N8080127). Gene expression was quantified by real-time quantitative PCR (qPCR) using iTaq Universal SYBR Green Supermix (Bio-Rad, 1725120). Relative expression levels were calculated using the ΔΔCt method with *act1*^*+*^ as the reference gene. Oligonucleotide sequences are listed in Supplementary Data 5. For RIP-qPCR, DNase-treated RNA from RIP samples was reverse transcribed as described above. Fold enrichment was calculated relative to the corresponding untagged control using *act1*^*+*^ normalization. Source data are included in the Source Data file.

For lariat PCR and sequencing to identify the *Tf2* intron branch site, 2 µg of DNase I-treated RNA was heat-denatured at 65 °C for 5 min. cDNA synthesis was performed using SuperScript III Reverse Transcriptase in the presence of 3 mM MgCl₂ and 3 mM MnCl₂ at 50 °C for 50 min with a reverse primer specific to the *Tf2* intron. PCR amplification was performed using Tf2 intron-specific primers. Amplicons were excised from ethidium bromide-stained gels, cloned into the pJET1.2 vector (CloneJET PCR Cloning Kit, Thermo Fisher Scientific, K1231), and subjected to Sanger sequencing using vector-specific primers.

### RNA sequencing

Total RNA was isolated as described above. Ribosomal RNA was depleted using the Ribo-Zero Gold Plus kit for human samples (Illumina, 20040892), supplemented with probes targeting *S. pombe* ribosomal DNA (Integrated DNA Technologies). Sequencing libraries were prepared using the NEBNext Ultra Directional RNA Library Prep Kit for Illumina (NEB, E7760S) according to the manufacturer’s instructions. Library quality and concentration were assessed using the Agilent High Sensitivity D1000 ScreenTape system. Libraries were sequenced on an Illumina NextSeq 500 platform using single-end reads.

### siRNA sequencing

Total RNA was isolated from 4 OD_600_ units of logarithmically growing cells using the MasterPure Yeast RNA Purification Kit (Biosearch Technologies, MPY03100) according to the manufacturer’s instructions. RNA samples were resolved on 15% urea–polyacrylamide gels, and small RNAs (15–30 nucleotides) were excised following staining with SYBR Gold nucleic acid gel stain (Thermo Fisher Scientific, S11494) under UV transillumination. Gel fragments were crushed, and RNA was eluted in the buffer provided with the NEBNext Multiplex Small RNA Library Prep Set for Illumina (NEB, E7300S) at 4 °C overnight with gentle rotation. Eluates were separated using Corning Costar Spin-X columns (Sigma-Aldrich, S11494) and precipitated with ethanol overnight. Pellets were resuspended in DEPC-treated water. Libraries were prepared using the NEBNext Multiplex Small RNA Library Prep Set according to the manufacturer’s protocol. Libraries were purified using Qiagen MinElute PCR purification columns, further size-selected on 6% PAGE gels, eluted, ethanol precipitated and resuspended in 1× TE buffer. Library quality was assessed using the Agilent High Sensitivity D1000 ScreenTape system. Sequencing was performed on an Illumina NextSeq 500 platform using single-end reads.

### RNA immunoprecipitation (RIP)

*S. pombe* cells expressing epitope-tagged proteins were grown in 100 ml YEA medium at 30 °C for 14 h or at 18 °C for 24 h (for gametogenic RNA pull-down experiments) to an OD_600_ of 0.3–0.5. Corresponding strains lacking the epitope tag were used as controls. Cells were crosslinked by the addition of paraformaldehyde to a final concentration of 3% and incubated for 30 min with gentle shaking. Crosslinked cells were pelleted by centrifugation at 900 × g for 5 min at 4 °C and washed with 1× PBS. Cells were subjected to secondary crosslinking using 10 mM dimethyl adipimidate (Sigma-Aldrich, 285625) for 45 min at room temperature to stabilize transient interactions. Cells were washed again with 1× PBS and resuspended in lysis buffer (50 mM HEPES, pH 7.9; 140 mM NaCl; 1 mM EDTA; 10% glycerol; 0.5% NP-40; 0.25% Triton X-100) supplemented with EDTA-free protease inhibitor cocktail (Roche, 4693132001) and 40 U RNase inhibitor (Thermo Fisher Scientific, AM2694). Cells were lysed by bead beating (Biospec Mini-Beadbeater-16) followed by sonication using a Bioruptor-300 (Diagenode) for 14 cycles (30 s ON, 30 s OFF) at 4 °C. A 20 µL aliquot of lysate was reserved as input. The remaining lysate was pre-cleared with 0.9 mg pre-washed Protein G Dynabeads (Invitrogen, 10004D) for 1 h at 4 °C. 2.5 µg of Anti-GFP antibody (Abcam, ab290) was added and incubated overnight at 4 °C with rotation. Immune complexes were captured using 1.2 mg Protein G Dynabeads for 2 h at 4 °C. Beads were washed sequentially (5 min each) with: lysis buffer; high-salt lysis buffer (300 mM NaCl); LiCl buffer (50 mM HEPES pH 7.5, 250 mM LiCl, 0.5% NP-40, 0.1% sodium deoxycholate); and 1× TE buffer.

RNA was eluted twice in 75 µL RIP elution buffer (50 mM Tris pH 8.0, 10 mM EDTA, 300 mM NaCl, 1% SDS) at 37 °C for 10 min. Input samples were adjusted to 150 µL with elution buffer. Samples were treated with 20 µg proteinase K (Thermo Fisher Scientific, AM2548) for 1 h at 37 °C and de-crosslinked at 65 °C for 1 h. RNA was extracted using phenol–chloroform, precipitated with ethanol, and resuspended in DEPC-treated water. Samples were treated with 20 U RNase-free DNase I (Thermo Fisher Scientific, AM2222) at 37 °C for 1 h, followed by purification as above. RNA concentrations were measured using a Qubit fluorometer (Thermo Fisher Scientific). Libraries were prepared using the NEBNext Ultra Directional RNA Library Prep Kit (NEB) and sequenced on an Illumina NextSeq 500 platform.

For TMG-RIP, Protein G Dynabeads (1.2 mg) were incubated with 10 µg anti-2,2,7-trimethylguanosine (m³G/TMG, MBL Life Science, RN019M, clone 235-1) monoclonal antibody overnight at 4 °C in IP buffer (50 mM Tris-HCl pH 7.4, 150 mM NaCl, 0.05% NP-40). Beads were washed three times and resuspended in fresh IP buffer containing RNase inhibitor. Total RNA (40 µg), heat-denatured at 65 °C for 5 min, was incubated with antibody-conjugated beads for 4 h at 4 °C. Beads were washed three times and RNA was eluted in 150 µL elution buffer (20 mM Tris-HCl pH 7.4, 300 mM sodium acetate pH 5.3, 2 mM EDTA, 0.25% SDS), followed by phenol–chloroform extraction and ethanol precipitation.

For RIP-qPCR, immunoprecipitated and input RNA samples were DNase-treated and reverse transcribed using SuperScript III Reverse Transcriptase with oligo(dT) and random hexamer primers. qPCR was performed using iTaq Universal SYBR Green Supermix (Bio-Rad). Fold enrichment was calculated using the ΔΔCt method with *act1*^*+*^ as the reference gene and normalized to untagged controls. The sequences of the oligonucleotides used for qPCR are listed in the Supplementary Data 5.

### Northern blot analysis

Northern blot analysis for centromeric siRNAs was performed as follows. Small RNAs ( < 200 nt) were isolated using the mirVana miRNA Isolation Kit (Thermo Fisher Scientific, AM1561). Twenty micrograms of RNA were resolved on a 15% denaturing polyacrylamide gel and transferred to Hybond-N+ membranes (Thermo Fisher Scientific, 11708308) in 0.5× TBE at 100 V for 1 h. Membranes were UV crosslinked and hybridized with α-³²P-UTP-labeled RNA probes (50 nt) corresponding to *dg/dh* sequences in UltraHyb-Oligo hybridization buffer (Thermo Fisher Scientific, AM8663).

For TER1 RNA analysis, 10 µg total RNA was treated with RNase H in the presence of target-specific oligonucleotides, resolved on a 6% TBE–urea gel, transferred to membranes, and hybridized overnight at 65 °C using ULTRAhyb ultrasensitive hybridization buffer (Thermo Fisher Scientific, AM8670) buffer.

### Chromatin immunoprecipitation and sequencing (ChIP-seq)

Cells were grown to OD_600_ = 0.4–0.5 and crosslinked with 3% paraformaldehyde for 30 min at room temperature. Crosslinking was quenched with 125 mM glycine. Cells were lysed in ChIP lysis buffer (50 mM HEPES-KOH pH 7.5, 140 mM NaCl, 1% Triton X-100 and 0.1% sodium deoxycholate, 1 mM PMSF, Roche cOmplete Mini Protease inhibitors cocktail) and chromatin was sheared to 0.4–0.6 kb fragments by sonication (Bioruptor; 14 cycles, 30 s ON/OFF). After centrifugation (1500 × g, 10 min, 4 °C), 2% of lysate was reserved as input. The remaining lysate was incubated with 2.5 µg antibody overnight at 4 °C. Chromatin complexes were captured using Protein A Sepharose 4B and Protein G Plus Agarose (1:1 slurry; Thermo Fisher Scientific) for 4 h. Beads were washed sequentially with lysis buffer, high-salt buffer (500 mM NaCl), wash buffer III (10 mM Tris-HCl pH 8, 250 mM LiCl, 0.5% IGEPAL, 0.5% sodium deoxycholate and 1 mM EDTA) and TE buffer. Chromatin was eluted twice by incubating in TE supplemented with 1% SDS for 30 min each at 65 °C, reverse crosslinked (16 h, 65 °C), treated for 90 min each with 5 µg RNase A (Thermo Fisher Scientific, 12091021) and 40 µg proteinase K (Thermo Fisher Scientific, 26160), and purified using QIAquick columns (QIAGEN, 28104). DNA was analyzed by qPCR or Illumina sequencing. Immunoprecipitated DNA and input DNA were analyzed by performing real-time PCR using iTaq Universal SYBR Green Supermix (BioRad). Fold enrichment was calculated by the ∆∆Ct method using *leu1*^*+*^ as the reference gene for normalization in all cases unless indicated otherwise. The sequences of the oligonucleotides used for ChIP–qPCR are listed in the Supplementary Data 5.

### Protein purification and mass spectrometry

Briefly, 2 L cultures each expressing the epitope-tagged Mug174 or Eas1 with corresponding untagged control cells were grown at 30 °C (*n* = 1 for Mug174 and Eas1 each). Cells were harvested at OD_600_ = 1 by centrifugation and flash-frozen in liquid nitrogen. Frozen cell pellets were lysed using a CryoMill (Retsch) through six cycles of pulverization (3 min per cycle at 30 Hz). The resulting frozen cell powder was thoroughly resuspended in lysis buffer (50 mM Tris-HCl, pH 7.5, 100 mM NaCl, 1.5 mM MgCl₂, 0.15% (v/v) IGEPAL, 0.5 mM DTT, 1 mM PMSF, and Roche cOmplete protease inhibitor cocktail). Cell lysates were cleared by centrifugation at 27,000 × g for 1 h at 4 °C. Cleared lysates were subjected to affinity purification using anti-GFP agarose beads (GFP-Trap, Chromotek, gta-20) for 2 h at 4 °C. Beads were washed extensively with BAC150 buffer (20 mM HEPES-KOH, pH 7.6, 20% glycerol, 2 mM MgCl₂, 1 mM EDTA, 150 mM KCl, 0.1% IGEPAL, 1 mM PMSF, and Roche cOmplete protease inhibitor cocktail). Bound proteins were eluted three times with 0.2 M glycine (pH 2.0). Eluted proteins were precipitated with trichloroacetic acid (TCA) and resolved on 4–12% Bis-Tris gels (Thermo Fisher Scientific). Protein bands were visualized using SimplyBlue SafeStain (Thermo Fisher Scientific, LC6060), excised, and subjected to mass spectrometry analysis.

Mass spectrometry sample preparation and analysis were performed as follows. Briefly, excised gel bands were subjected to in-gel trypsin (20 ng/µl) digestion to extract the peptides. Samples were desalted using the Pierce C18 spin columns (ThermoFisher Scientific), dried and resuspended in 0.1% trifluoroacetic acid prior to loading onto an Acclaim PepMap 100C18 LC column (ThermoFisher Scientific) utilizing a Thermo Easy nLC 1000 LC system (ThermoFisher Scientific) connected to the Q Exactive HF mass spectrometer (ThermoFisher Scientific). The peptides were eluted with a 5 to 36% gradient of acetonitrile with 0.1% formic acid over 56 min with a flow rate of 300 nL/min. The QEHF was operated with each MS1 scan in the orbitrap at 60,000 resolution with a maximum injection time of 120 ms and an AGC target of 1e6. The MS2 scans with a normalized collision energy of 27 were run at 15,000 resolution with a maximum injection time of 50 ms and an AGC target of 2e5. The raw MS data were analyzed in Proteome Discoverer 2.2 (Thermo Fisher Scientific) with SEQUEST HT software and was searched against the UniProt *S. pombe* proteome database from the European Bioinformatics Institute (https://www.uniprot.org/proteomes/UP000002485). The parent ion mass tolerance was set to 10 ppm and the fragment ion mass was 0.02 Da. A maximum of two missed cleavages and a minimal peptide length of six amino acids were allowed.

### Immunoprecipitation analyses

Cells expressing epitope-tagged proteins under the control of their native gene promoters were used for immunoprecipitation (IP) and immunoblot analyses as follows. For GFP pull-down assays, cells from 2 L cultures grown to OD_600_ = 1 were harvested, flash-frozen in liquid nitrogen, mixed with dry ice, and pulverized using a household blender. The resulting cell powder was resuspended in lysis buffer (as described in the protein purification protocol), and lysates were cleared by centrifugation at 27,000 × g for 1 h at 4 °C. Cleared lysates were subjected to affinity purification using anti-GFP agarose beads for 2 h at 4 °C. Beads were washed extensively with BAC150 buffer, and bound proteins were eluted with 0.2 M glycine (pH 2.0). Eluted proteins were precipitated with trichloroacetic acid (TCA), resuspended in SDS–PAGE sample buffer, and resolved on 4–12% Bis-Tris gels for western blot analysis. Primary antibodies used were anti-GFP (Roche), anti-Myc (Santa Cruz Biotechnology), and anti-FLAG M2 (Sigma-Aldrich) at 1:1000 dilution. HRP-conjugated secondary antibodies (anti-mouse or anti-rabbit; GE Healthcare) at 1:2500 dilution were used for detection (see below).

### Immunoblot analysis

Total cell lysates from *S. pombe* were prepared by lysing 10 OD_600_ units of freshly grown cells in 20% (w/v) trichloroacetic acid (TCA), followed by bead beating using a Biospec Mini-Beadbeater-16. TCA-precipitated proteins were washed with ethanol and resuspended in SDS–PAGE sample buffer. Equal amounts of protein were resolved on NuPAGE Bis-Tris polyacrylamide gels (Thermo Fisher Scientific) and transferred to PVDF membranes. Primary antibodies used were anti-GFP (Roche, Catalog#11814460001; 1:1000 dilution), anti-HA (Santa Cruz, Catalog# Sc-7392; 1:1000 dilution), anti-c-Myc 9E10 (Santa Cruz, Catalog#sc-40; 1:1000 dilution), anti-FLAG M2 (Sigma Aldrich, Catalog#F3165; 1:1000 dilution) or anti-Cdc2 (Santa cruz, Catalog#sc-53217) as indicated in the experiment. 1:2500 dilution of secondary anti-mouse IgG1 (GE Healthcare, Catalog#NA931) HRP linked antibody was used. Uncropped blots are shown in the Source Data file. For human cell lines, whole-cell extracts were prepared by resuspending cells in RIPA buffer (25 mM Tris-HCl, pH 7.5, 150 mM NaCl, 1% sodium deoxycholate, 1% IGEPAL CA-630, 0.1% SDS) supplemented with protease inhibitor cocktail (Roche, 11873580001). Samples were incubated on ice for 10 min and sonicated for 15 s on high power using a Diagenode Bioruptor. Lysates were clarified by centrifugation at 21,000 × g for 10 min at 4 °C, and the supernatant was transferred to fresh tubes.

Equal amounts of protein were mixed with 4× NuPAGE LDS Sample Buffer (Thermo Fisher Scientific, NP0007) and 1× NuPAGE Sample Reducing Agent (Thermo Fisher Scientific, NP0009) and denatured at 95 °C for 3 min prior to electrophoresis. Primary antibodies were diluted in 1% (w/v) milk in TBS-T and used at 1:1000 dilution: anti-PIMT/TGS1 (Bethyl Laboratories, A300-814A) and anti-GAPDH (Abcam, ab8245). HRP-conjugated secondary antibodies (anti-mouse IgG1, GE Healthcare, NA931; anti-rabbit IgG, GE Healthcare, NA934) were used at a dilution of 1:2500.

### Microscopy and image analysis

Cells were grown overnight at 30 °C in minimal PMG medium until they reached logarithmic phase. Cells were then mounted in 2% agarose. Eighteen 0.2 µm z-sections were acquired on a Delta Vision Elite microscope (Applied Precision, GE Healthcare) with a 100× 1.35 NA oil lens (Olympus). Images were subject to deconvolution and processed using ImageJ (ImageJ2 1.53 v).

### Recombinant expression and purification of Tgs1 and its mutant derivatives

*S. pombe* Tgs1 and its mutant tgs1^G154D^, were synthesized and cloned into *E. coli* expression vector pET28 (GenScript). The pET28-(His)_6_-Tgs1 plasmids were transformed into *E. coli* BL21(DE3) CodonPlus cells, and the transformation mixture was plated onto LB plates containing 50 µg/ml kanamycin. After overnight incubation of the plates at 37 °C, a mixture of colonies was grown in 50 ml of LB medium containing 50 µg/ml kanamycin and 25 µg/ml chloramphenicol for two hours at 37 °C, and then the culture was diluted into 500 ml of LB medium containing 50 µg/ml kanamycin and 25 µg/ml chloramphenicol and grown until the A_600_ reached about 0.6. The culture was cooled down briefly on ice and adjusted to 2% ethanol and 0.25 mM isopropyl-1-thio-D-galactopyranoside. The culture was then incubated at 16 °C for 20 h with constant shaking. Cells were harvested by centrifugation, and the pellet was stored at −80 °C. All subsequent procedures were performed at 4 °C. Thawed bacteria were resuspended in 25 ml of buffer A (50 mM Tris-HCl, pH 8.0, 300 mM NaCl, 10% glycerol) containing 1× Complete proteinase inhibitors (Roche), sonicated, and insoluble material was removed by centrifugation at 16,000 × g for 20 min in a JA20 rotor at 4 °C. The soluble extract was applied to 0.8 ml of PureCube 100 Ni- INDIGO agarose resin (Cube Biotech) equilibrated with buffer A. The beads/extract mixture was incubated with gentle rotation for 60 min at 4 °C, and beads were washed with 25 ml of buffer A containing 5–10 mM imidazole. The beads were then transferred to a Poly-Prep Chromatography column (Bio-Rad) and washed with 10 ml of the same buffer. Bound proteins were eluted with 1 ml aliquots of buffer A containing 250 mM imidazole. The eluted fractions were analyzed by SDS-PAGE. Fractions predominantly containing the (His)_6_-Tgs1 WT or mutant polypeptide were combined and dialyzed against buffer containing 50 mM Tris-HCl, pH 8.0, 100 mM NaCl, 2 mM DTT, 1 mM EDTA, 10% glycerol, and aliquots were quick-frozen in liquid nitrogen and stored at 80 °C. The protein concentration of the band corresponding in size to (His)_6_-Tgs1 was determined by SDS-PAGE analysis of serial dilutions of the (His)_6_-Tgs1 preparations and a BSA standard. Gels were stained with SYPRO Orange and scanned using Typhoon FLA9500 (GE), and the staining intensities of the (His)_6_-Tgs1 and BSA polypeptides were quantified using ImageQuant (GE Healthcare).

### Methyltransferase assay

Reaction mixtures (20 µl) containing 50 mM Tris-HCl (pH 8.0), 5 mM DTT, 50 µM [3H-CH_3_] AdoMet, 5 mM m7GTP, and 100 ng of purified WT or tgs1^G154D^ recombinant Tgs1 enzyme were incubated for 60 min at 37 °C. The mixtures were spotted on DEAE-cellulose filters (25 mm, Whatman DE81), which were washed four times with 20 mM ammonium bicarbonate. The filters were dried, and the radioactivity adsorbed to the filter was quantified by liquid scintillation counting. Methyltransferase assays were performed at least three times to ensure reproducibility and for signal quantification.

### Statistics and reproducibility

Statistical parameters are reported in the figures and figure legends. Three or more independent biological replicates (n), indicated as individual data points on each graph, were performed, and the mean ± SD is presented in each case. Statistical analyses were performed using GraphPad Prism (v10.2.3). One-way Anova followed by Dunnett’s or Tukey’s multiple comparisons, or a two-tailed unpaired t-test was used as appropriate. Statistical significance and *P*-values are as indicated in the figures. For Euler diagrams, the expected distribution of the counts of transcripts common to multiple, uncorrelated, transcript sets (N-way counts) were estimated by sampling. For each transcript set in the diagram, N_set_ random transcripts were sampled from the set of all transcripts (N_all_=7015), where N_set_ is the number of transcripts in a set. This procedure was performed 10000 times and the resulting 10,000 random N-way counts were recorded. The *P*-value for an N-way intersection with observed count O was subsequently computed as: sum (C_i_ > O)/10,000; where C_i_ is the ith random N-way count computed as above, and O is the observed N-way count to be evaluated. For all qRT-PCR and qPCR experiments three biological replicates were performed, and the data points are shown. For immunoblots and co-Ips at least 2 independent biological replicates were performed with similar results. For RNA-seq, small RNA -seq and RIP-seq two or more independent biological replicates were performed with similar results.

### Processing of ChIP-seq data

Reads were quality trimmed with fastp^79^ and aligned using the BWA aligner^80^ to the *S. pombe* V2 reference sequence^81^ in which the Pombase provided 20 kb *mat* contig was replaced with the sequence of the full 40 kb *mat* region. In addition, both the *mat* region on chromosome 2 (chrII 2,109,748–2,138,781) and the *mat1M* locus on the 40 kb *mat* contig (MAT 4,489–5,615) were masked from alignment. Bedgraphs of ChIP enrichment over input were produced using the MACS2^82^ ‘callpeak’ function to make broad calls with options ‘–nomodel–extsize 147*’*, followed by the MACS2 ‘bdgcmp’ function to compute fold enrichment over the input background. The standard variable interval bedgraphs were further processed to produce 10 bp fixed interval versions to facilitate precise alignment during figure construction.

### Alphafold predictions and structural alignments

Predicted structures for Eas1 (SPAC18G6.13) and the N-terminal portion of Mug174 (SPCC1682.03c) were taken from the AlphaFold Protein Structure Database^83^. Structural alignments were computed using the ‘matchmaker’ function of ChimeraX, version 1.7.

### Splicing efficiency

Splicing efficiency was determined for sites of canonical intron splicing taken from Pombase annotations^81^ as well as for published non-canonical, ‘cryptic’, splice sites^19^. Splicing efficiency was measured from RNA-seq read alignments in BAM format using the pysam Python library. Introns were first identified using the pysam ‘find introns’ method. For each detected intron with junctions at *j5* and *j3* bp, counts for spliced reads supporting the intron were obtained as cnt0. The sums of the counts of spliced reads (supporting splicing success) and unspliced reads (supporting splicing failure) spanning each of the intervals (*j5*-1, *j5* + 1) and (*j3*-1, *j3* + 1) were then obtained as *cnt1* and *cnt2*, respectively. Counts *cnt1* and *cnt2* are independent estimates of the total transcript number and the minimum of the two was used to compute splicing efficiency for each intron as cnt0/min(cnt1, cnt2)*100%.

### Processing of RNA-seq data

Single-ended short reads from RNA-seq experiments were quality trimmed using fastp^79^ and aligned to *S. pombe* using the STAR aligner^84^. Variable interval bedgraphs, normalized to counts per million mapped reads and including both uniquely and multi-mapping reads, were generated by STAR and were further processed to produce 10 bp fixed interval versions for purposes of figure construction. Read counts for transcripts were computed from BAMs using Rsubread^85^, and differential expression with respect to WT controls was computed using DEGseq^86^.

### Alignment-based expression estimates for mRNA and ncRNA transcripts in human siRNA KDs

Short reads were quality trimmed using fastp^79^ and aligned to the T2T.CHM13v2.0 human genome sequence^87^ using the STAR aligner. Aligned reads were assigned to transcripts included in the T2T.CHM13v2.0 gene annotations using Rsubread^85^ with default parameters. Transcript expression changes were estimated from the resulting feature counts independently for each of two replicates using DEGseq.

### Salmon expression estimates for mRNA and ncRNA transcripts

T2T.CHM13v2.0 transcript sequences were generated from the T2T genomic sequence using the coordinates from T2T.CHM13v2.0 mRNA and ncRNA transcript annotations. A Salmon database was constructed from these transcript sequences with the full T2T genomic sequence included as “decoy”. Salmon^88^ ‘quant’ was subsequently run using standard parameters to assign short reads to transcript sequences in the database. Transcript expression changes were estimated from the resulting Salmon counts using DESeq2^89^ with two replicates.

### Salmon expression estimates for repetitive element transcripts

Coordinates on the hg19 genomic reference^90^ of repetitive elements were taken from the Repeatmasker provided ‘hg19.fa.out.gz’ file (v 4.0.5). After removal of simple, low-complexity repeats, the sequence for each remaining repetitive element was extracted from the hg19 genomic sequence using the coordinates provided. A Salmon^88^ database was constructed from ~4.6 M such repetitive element sequences with the full hg19 genomic sequence included as the ‘decoy’. Salmon ‘quant’ was subsequently run using standard parameters to assign short reads to repetitive element sequences in the database. Repetitive element expression changes were estimated from the resulting Salmon counts using DESeq2^89^ with 2 replicates.

### Processing of RIP-seq data

Single-ended short reads from RIP-seq experiments were aligned as described for RNA-seq data. Read counts for transcripts were computed from the resulting BAM files using Rsubread^85^ for both Rips and matched untagged controls. Total counts per transcript were normalized to counts per million mapped reads per 1 Kb after which the normalized untagged counts were subtracted from the corresponding normalized Rip counts. Negative values were converted to zero.

### Processing of small RNA-seq data

Single-ended short reads were quality trimmed with fastp^79^ and aligned to the *S. pombe* reference using Novoalign (www.novocraft.com/products/novoalign). SGR files, whose format allows the simultaneous display of read coverage from both strands, were subsequently created from the Novoalign-generated BAM files using custom scripts (available on request). SGRs were either filtered to remove multi-mapping reads or left unfiltered to include multi-mapping reads and subsequently normalized to counts per 10 million mapped reads (CP10M).

### Identification of mutations by whole genome sequencing

NextSeq 500 sequencing generated an average of 25 million single-end reads per mutant or reference sample. NGS reads were first quality trimmed using fastp^79^ and aligned using the BWA Aligner^80^ to the *S. pombe* reference genome using default parameters with the addition of the “-M” option for compatibility with Picard Tools. Alignments yielded a mean coverage of ~150x per sample, a mean mapping quality >=55, and >=30x coverage over 95% of the genome. Duplicate reads were removed using Picard Tools ‘markduplicates’ (http://broadinstitute.github.io/picard) after which SAMtools^80^ ‘mpileup’ was used to compute genotype likelihoods from the alignments. Single nucleotide polymorphisms (SNPs), insertions, and deletions were called from the genotype likelihoods using VCFtools^91^ to produce Variant Call Format (VCF) files after which the variants were annotated using SnpEff^92^. Variant sites were supported by >=30x coverage in both reference and mutant alignments. The annotations produced by SnpEff include the name of the gene affected, the expected amino acid change in the case of variants in coding regions, and an assessment of the probable impact of the variant (HIGH, MODERATE, LOW, MODIFIER). Variants with either HIGH or MODERATE impact include those that introduce premature stop codons, frameshifts or single amino acid changes, and these were selected for further consideration if they were found in the mutant strain but not in the matched WT.

### Visualization

The Integrative Genomics Viewer^93^ (v.2.13.1) was used to visualize bedgraphs of NGS alignments. The Integrated Genome Browser was used to visualize small RNA SGR alignments. Heatmaps were produced using ComplexHeatmap^94^. Euler Diagrams were produced using eulerR (https://jolars.github.io/eulerr/). This work utilized the computational resources of the NIH HPC Biowulf cluster (http://hpc.nih.gov).

### Reporting summary

Further information on research design is available in the Nature Portfolio Reporting Summary linked to this article.

## Supplementary information

Supplementary Information

Description of Additional Supplementary Files

Supplementary Data 1

Supplementary Data 2

Supplementary Data 3

Supplementary Data 4

Supplementary Data 5

Reporting Summary

Transparent Peer Review file

## Source data

Source Data

## Data Availability

RNA-seq, RIP-seq, small RNA-seq and ChIP-seq datasets generated in this paper are deposited in the Gene Expression Omnibus (GEO). The RNA-seq data are available with accession GSE300777, RIP-seq with the accession number GSE300779, small RNA-seq with the accession GSE300776, and ChIP seq with the accession GSE300775. The mass spectrometry data are deposited in the MassIVE database with accession number MSV000100022. Source data are provided with this paper.

## Code availability

The softwares used to analyze the data is listed in Methods and is publicly available. The custom code used in this study is publicly available at Zenodo (10.5281/zenodo.14285969).
