# Supplementary material for: Spliceosomal proteins direct RNA methylation to modulate gene expression and silence retrotransposons
Source: Nat Commun. 2026 Jul 8;17:8405. doi: 10.1038/s41467-026-75362-5 (PMC13473130; doi:10.1038/s41467-026-75362-5)

Supplementary Information

Spliceosomal proteins direct RNA methylation to modulate gene expression and silence retrotransposons

Drisya Vijayakumari, Xander Gottfried, Brent Groubert, Jothy Dhakshnamoorthy, Shweta Jain, Martin Zofall, Hernan Diego Folco, Hua Xiao, Anupa T Anil, Thorkell Andresson, David Wheeler, Shiv I.S. Grewal *

Supplementary Figures 1-10

*Corresponding author

Email: grewals@mail.nih.gov

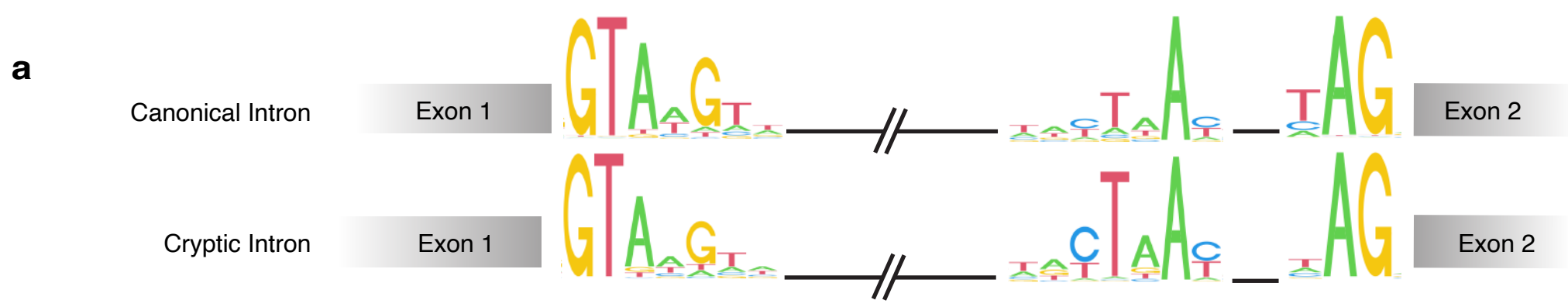

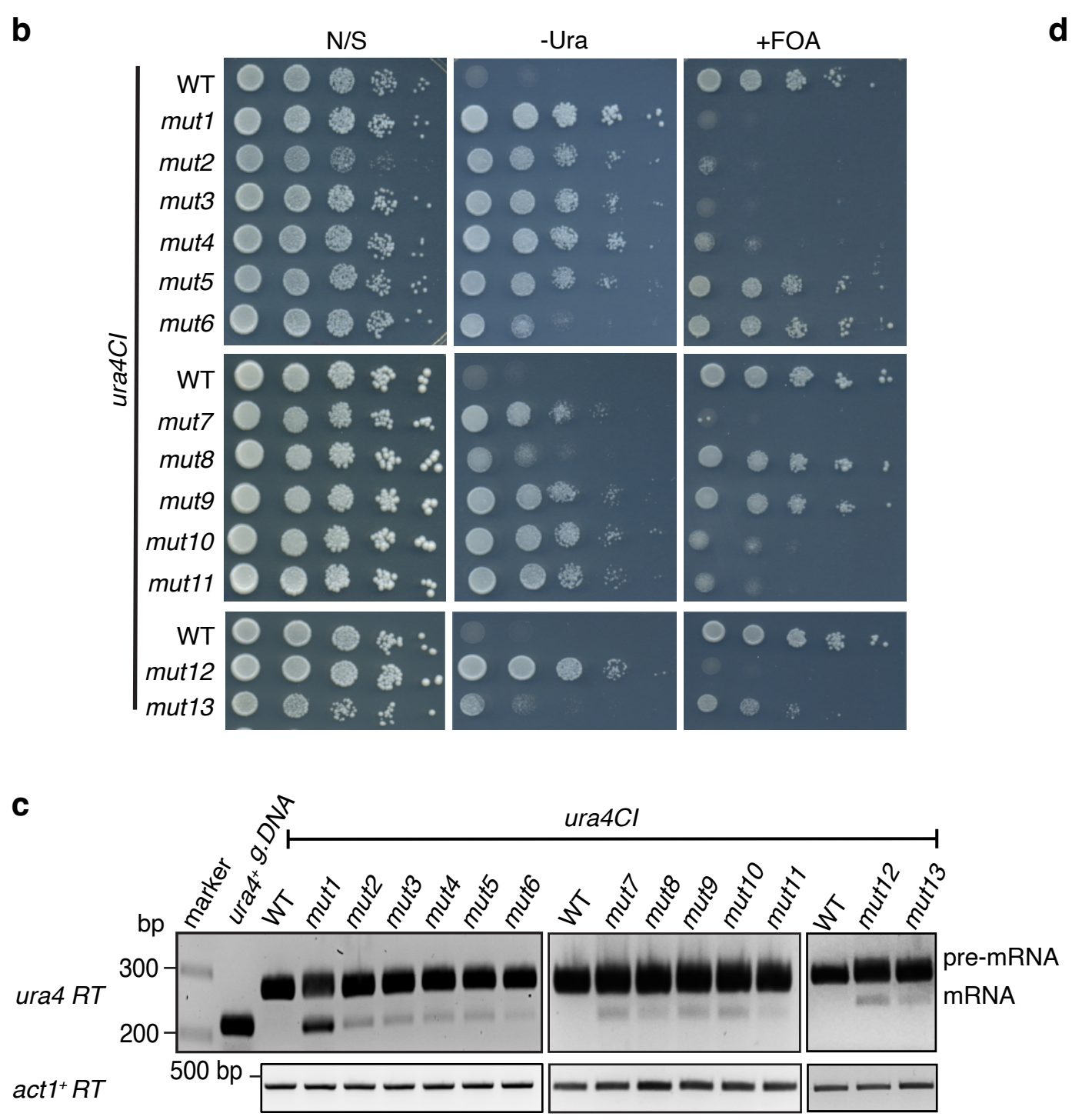

d

Mutant	Mutation	Protein
mut1	CI^{BSmut}	Cryptic intron
mut2	tgs1R39*	Tgs1
mut3	tgs1C83Y	Tgs1
mut4	tgs1T55I P152S	Tgs1
mut5	mug174V32M	Mug174
mut6	luc7R205H	Luc7
mut7	mug174W17*	Mug174
mut8	SPAC18G6.13 W107*	SPAC18G6.13
mut9	SPAC18G6.13 W38*	SPAC18G6.13
mut10	SPAC18G6.13 Q50*	SPAC18G6.13
mut11	SPAC18G6.13 I74N	SPAC18G6.13
mut12	tgs1R122*	Tgs1
mut13	bpb1R228C	Bpb1
mut14	SPAC18G6.13 M1I	SPAC18G6.13
mut15	SPAC18G6.13 V105G E106*	SPAC18G6.13
mut16	CI^{BSmut}	Cryptic intron
mut17	mug174Q185*	Mug174
mut18	mug174W131*	Mug174
mut19	mug174V32M	Mug174
mut20	tgs1G82E	Tgs1
mut21	tgs1D78N	Tgs1
mut22	tgs1G154D	Tgs1

Supplementary Fig. 1 | Genetic screen identifies Tgs1, SPAC18G6.13 and Mug174 as factors affecting cryptic intron splicing. a, Composite diagrams of sequence logos for the 5' splice site (5'SS), branch site (BS) and 3' splice site (3'SS) in *S. pombe* canonical and cryptic introns. The list of cryptic introns used is compiled from RNA-seq data of Lee et al.,[19] Thillainadesan et al.,[20] and this study. **b**, Ten-fold serial dilution of the *ura4CI* containing WT and mutant strains spotted on non-selective (N/S), uracil lacking (-Ura) and the counter-selective FOA (+FOA) containing media. **c**, RT-PCR analysis to assess splicing of the *ura4* cryptic intron in the indicated strains. *act1$^+$* served as the control. **d**, Table showing the list of mutants recovered from the screen with the identified mutations. (*) indicates stop codon.

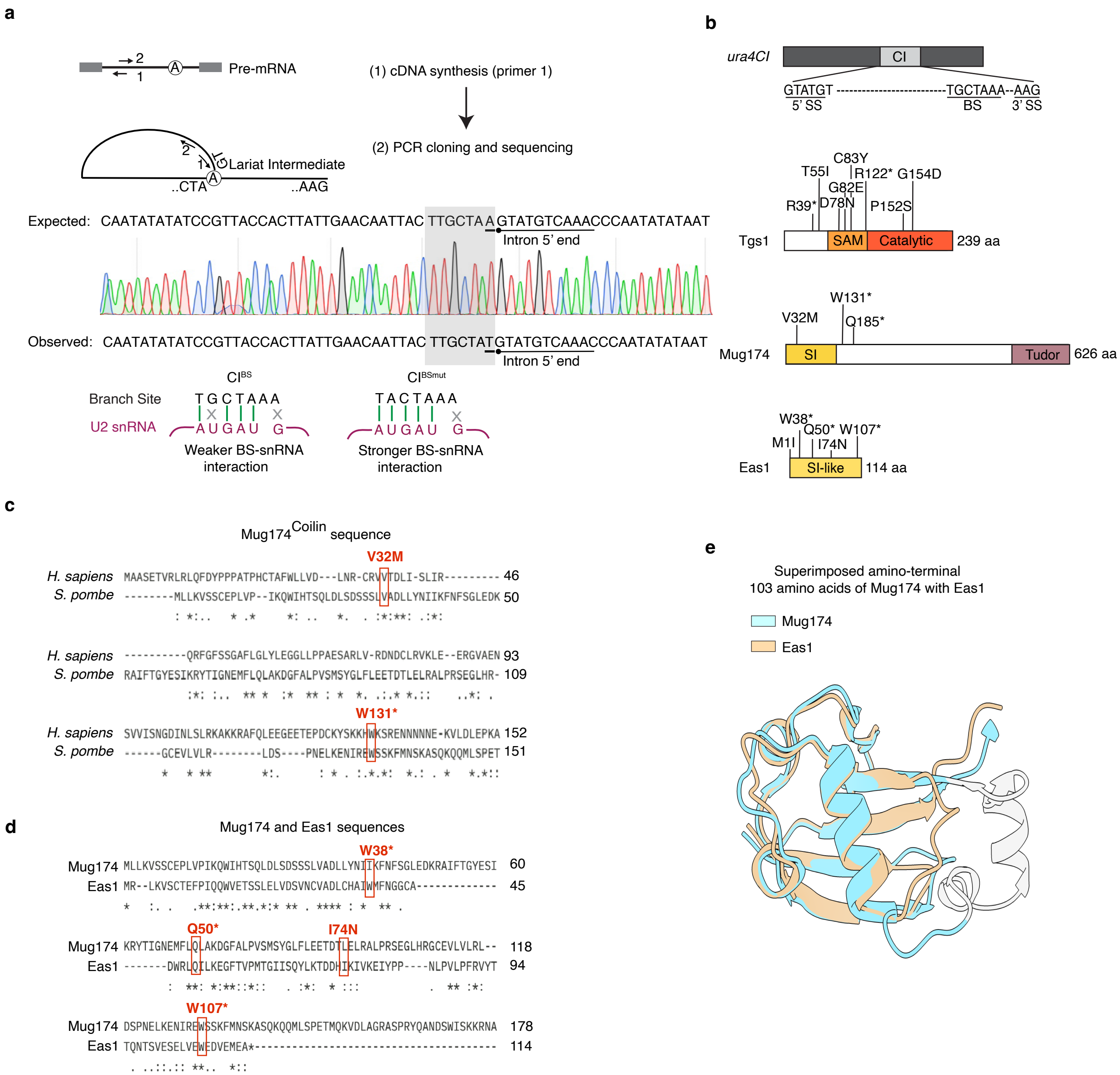

Supplementary Fig. 2 | *Cis-* and *trans*-acting determinants of cryptic intron splicing. a, Schematic depicting the workflow of lariat PCR performed to identify the branch site of the *Tf2* cryptic intron. The chromatogram represents the result of lariat sequencing. The reverse transcriptase traversing through the branch point during cDNA synthesis tends to skip the branch point A and introduces error at the adjacent residue converting A to T in the observed chromatogram profile. Base-pairing of the original (*CI*^{BS}) and mutant branch site (*CI*^{BSmut}) with the corresponding residue in U2 snRNA is shown. **b**, Schematic depicting the domain architecture of *trans*-acting factors identified in the screen. For the *ura4CI* schematic, the 5' splice site, branch site and 3' splice site sequences of the intron are shown. Abbreviations used: CI- cryptic intron, SAM- S-adenosyl methionine, and SI- self interaction. The locations of mutations obtained in the screen are indicated. **c**, Sequence alignment of the N-terminal region of Coilin (*H. sapiens*) and Mug174 (*S. pombe*) with mutations identified in Mug174 highlighted. **d**, Sequence alignment of the N-terminal region of Mug174 with full-length Eas1. Mutations identified in Eas1 are highlighted. **e**, Superimposition of the predicted structure (AlphaFold Protein Structure Database) of the N-terminal region 103 amino-acids of Mug174 with the N-terminal 82 amino-acids of Eas1 and structural alignments computed using ChimeraX. An alpha-helix and short loop that is unique to Mug174 and therefore unaligned, is shown in grey.

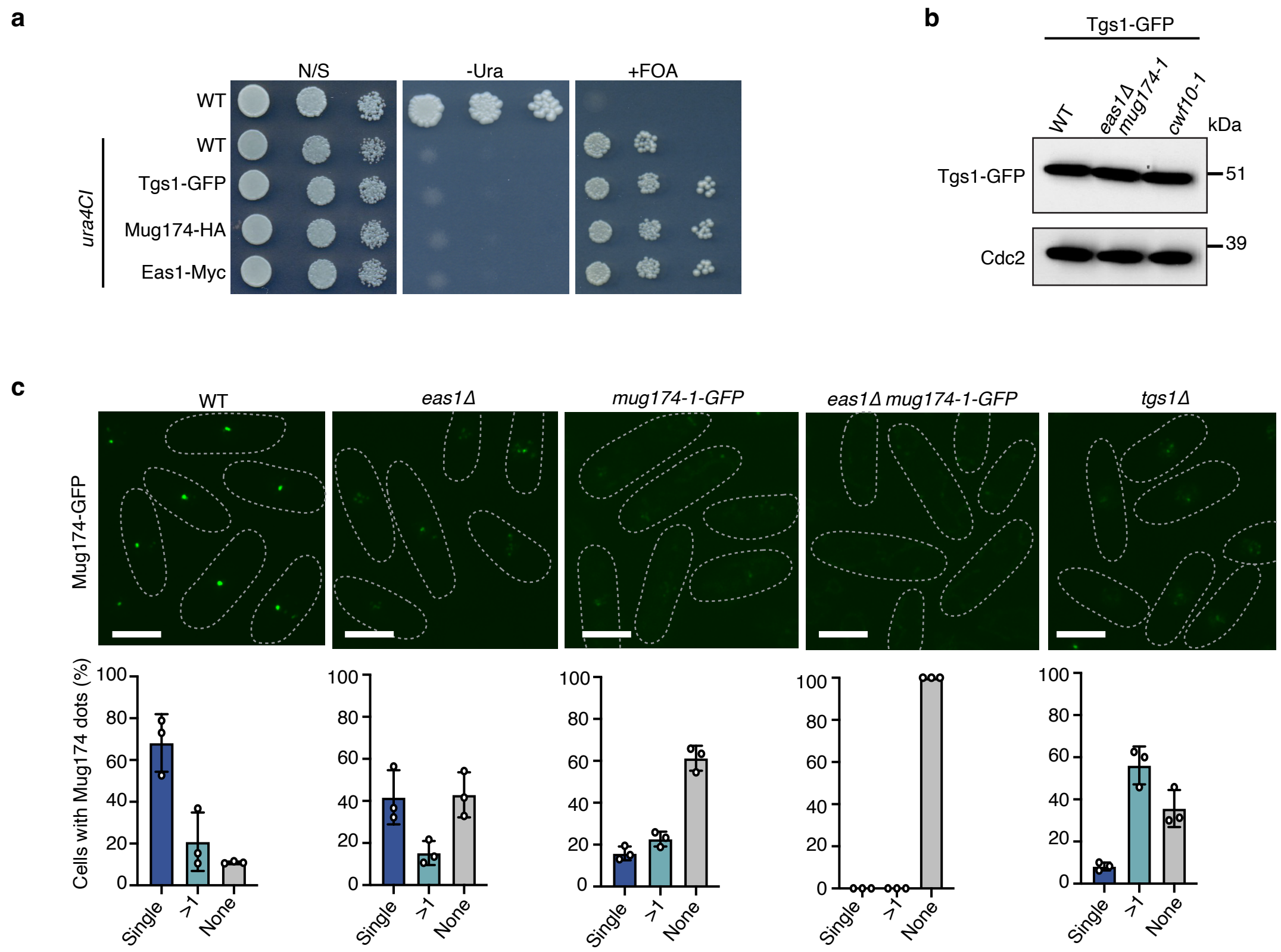

Supplementary Fig. 3 | Formation of Mug174 foci is dependent on other TEaM components. **a,** Ten-fold serial dilution of the WT and GFP tagged strains in the *ura4CI* background spotted on non-selective (N/S), uracil lacking (-Ura) and the counter-selective 5-FOA (+FOA) containing media. WT strain with *ura4*+ serves as the control for growth on -Ura and lack of growth on 5-FOA. **b**, Immunoblot of Tgs1-GFP protein levels in WT, *eas1Δ mug174-1* and *cwf10-1* mutant cells. **a, b**, Data are reprsentative of two independent experiments with similar results**. c**, Live cell imaging of Mug174-GFP in the indicated strains. Scale bars, 5 *μ*m. The percentage of cells showing single foci vs multiple or no foci in each strain background is indicated. Atleast n = 75 cells were counted for each strain. Data are presented as mean ± SD. Source data are provided as a source data file.

ura4

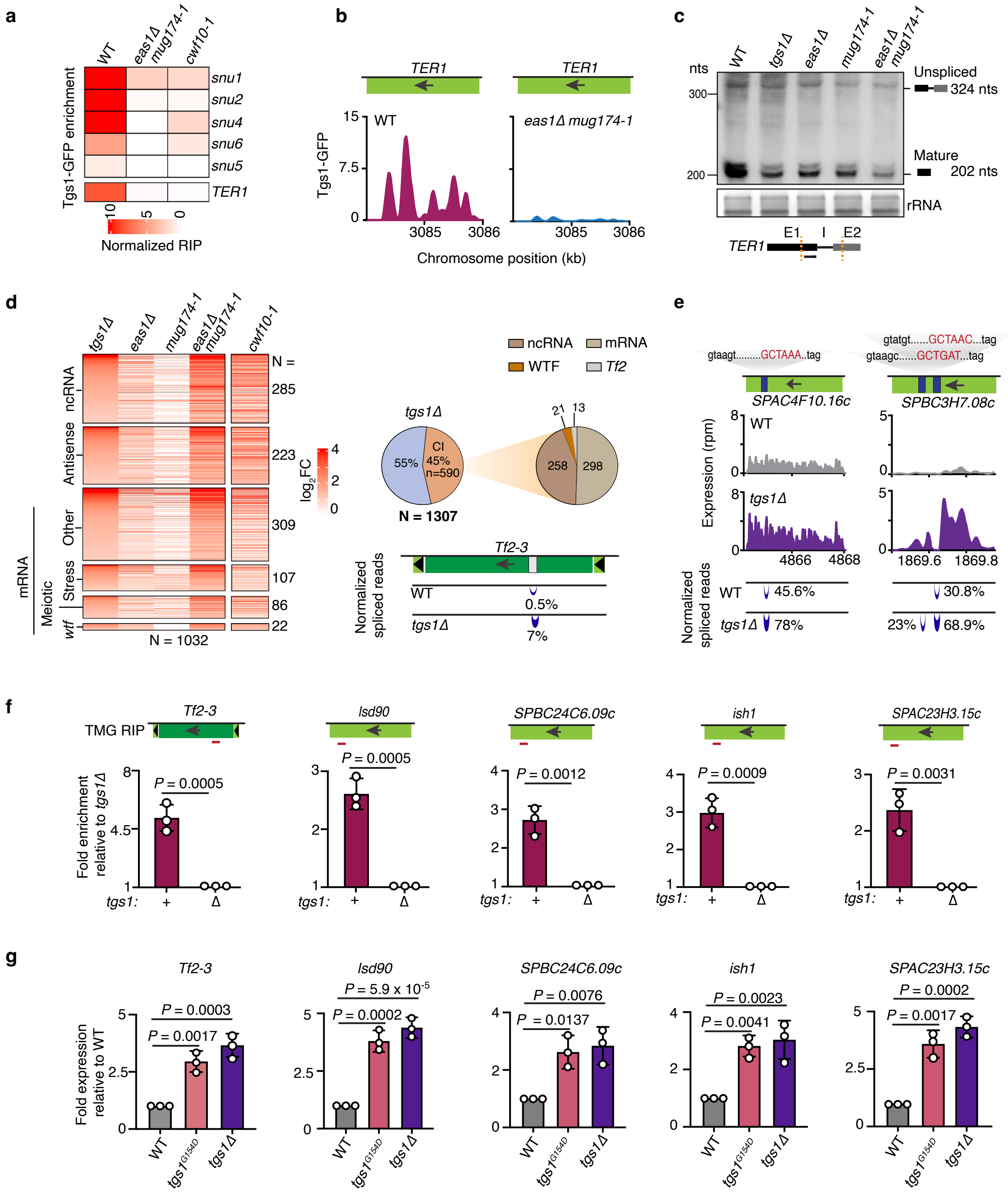
a
WT
eas1Δ mug174-1
cwf10-1
snu1
snu2
snu4
snu6
snu5
TER1
Tgs1-GFP enrichment
10 5 0
Normalized RIP
b
TER1
TER1
WT
eas1Δ mug174-1
Tgs1-GFP
15
7.5
0
3085
3086
Chromosome position (kb)
c
nts
WT
tgs1Δ
eas1Δ
mug174-1
eas1Δ mug174-1
300
200
Unspliced
324 nts
Mature
202 nts
rRNA
TER1
E1
I
E2
d
tgs1Δ
eas1Δ
mug174-1
eas1Δ mug174-1
cwf10-1
N =
ncRNA
285
Antisense
223
Other
309
Stress
107
Meiotic
86
wtf
22
mRNA
N = 1032
log2FC
4
2
0
ncRNA
mRNA
WTF
Tf2
tgs1Δ
55%
CI 45% n=590
N = 1307
21
13
258
298
Tf2-3
Normalized spliced reads
WT
0.5%
tgs1Δ
7%
e
gtaagt.........GCTAAA..tag
gtatgt......GCTAAC...tag
gtaagc......GCTGAT...tag
SPAC4F10.16c
SPBC3H7.08c
Expression (rpm)
WT
tgs1Δ
8
0
5
4866
4868
1869.6
1869.8
Normalized spliced reads
WT
45.6%
30.8%
tgs1Δ
78%
23%
68.9%
f
TMG RIP
Tf2-3
lsd90
SPBC24C6.09c
ish1
SPAC23H3.15c
Fold enrichment relative to tgs1Δ
P = 0.0005
P = 0.0005
P = 0.0012
P = 0.0009
P = 0.0031
tgs1:
+
Δ
g
Tf2-3
lsd90
SPBC24C6.09c
ish1
SPAC23H3.15c
Fold expression relative to WT
P = 0.0003
P = 0.0017
P = 5.9 x 10-5
P = 0.0002
P = 0.0076
P = 0.0137
P = 0.0023
P = 0.0041
P = 0.0002
P = 0.0017
WT
tgs1G154D
tgs1Δ

Supplementary Fig. 4 | TEaM targets diverse RNA species including *Tf2* retrotransposon and gene transcripts. **a**, Heatmap of Tgs1-GFP enrichment (RIP-seq) on snRNAs in WT, *eas1Δ mug174-1* and *cwf10-1* mutant strains. **b**, Tgs1-GFP enrichment profile (RIP-seq) on TER1 RNA in WT and the *eas1Δ mug174-1* mutant. **c**, Northern blot analysis to examine *TER1* processing in the indicated strains. Schematic depicts TER1 RNA with the location of the probe indicated with a black solid line and the regions digested by oligo-directed RNaseH as dotted lines. **d**, Left: Heatmap representation of 1032 transcripts commonly upregulated in *tgs1Δ* and *eas1Δ mug174-1* mutant cells (≥ 2-fold) relative to WT. Top right: Percentage of CI-containing transcripts in *tgs1Δ* upregulated category of 1307 transcripts and further classification of the upregulated CI-containing transcripts by type. Bottom right: The normalized number of spliced reads mapping to *Tf2* cryptic intron in WT and *tgs1Δ*. **e**, Normalized number of spliced reads mapping to annotated introns with sub-optimal splice features in WT and *tgs1Δ* cells. **a-e**, Experiments were performed in two independent biological replicates with similar results. **f**, RT-qPCR analysis was performed to determine the enrichment of TMG cap on the indicated transcripts in anti-TMG cap RIP samples. Fold enrichment was calculated relative to *tgs1Δ*. The *abp1DDE* mutation is used in all genetic backgrounds to enable *Tf2* transcript detection. Data are presented as mean ± SD; n = 3 independent experiments, *P*-value by two-tailed unpaired t tests. **g**, RT-qPCR analysis was performed to measure transcript levels in $tgs1^{G154D}$ and *tgs1Δ* mutant strains. Results are expressed as fold change relative to WT. Data are presented as mean ± SD; n = 3 independent experiments. *P*-values were determined by one-way ANOVA followed by Dunnett's multiple comparisons test. (**f, g**) Position of oligos used for RT-qPCR are indicated by the red bar. Source data are provided as a source data file.

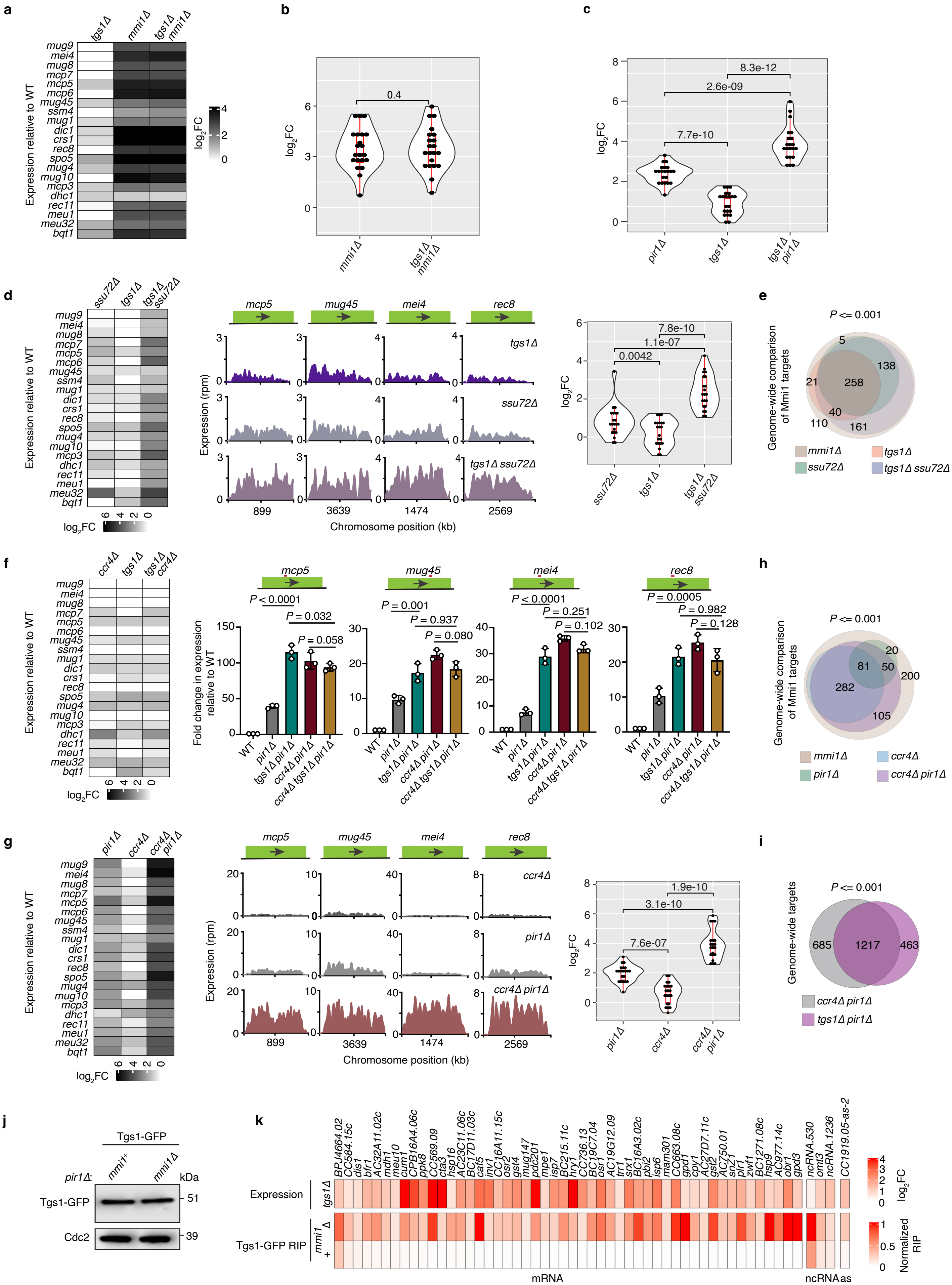
a
tgs1Δ
mmi1Δ
tgs1Δ mmi1Δ
mug9
mei4
mug8
mcp7
mcp5
mcp6
mug45
ssm4
mug1
dic1
crs1
rec8
spo5
mug4
mug10
mcp3
dhc1
rec11
meu1
meu32
bqt1
Expression relative to WT
log_2FC
b
0.4
log_2FC
mmi1Δ
tgs1Δ mmi1Δ
c
8.3e-12
2.6e-09
7.7e-10
log_2FC
pir1Δ
tgs1Δ
tgs1Δ pir1Δ
d
ssu72Δ
tgs1Δ
tgs1Δ ssu72Δ
Expression relative to WT
log_2FC
mcp5
mug45
mei4
rec8
tgs1Δ
ssu72Δ
tgs1Δ ssu72Δ
Expression (rpm)
899
3639
1474
2569
Chromosome position (kb)
7.8e-10
1.1e-07
0.0042
log_2FC
e
P <= 0.001
Genome-wide comparison of Mmi1 targets
5
138
21
258
40
110
161
mmi1Δ
tgs1Δ
ssu72Δ
tgs1Δ ssu72Δ
f
ccr4Δ
tgs1Δ
tgs1Δ ccr4Δ
Expression relative to WT
log_2FC
mcp5
mug45
mei4
rec8
P < 0.0001
P = 0.032
P = 0.058
P = 0.001
P = 0.937
P = 0.080
P < 0.0001
P = 0.251
P = 0.102
P = 0.0005
P = 0.982
P = 0.128
Fold change in expression relative to WT
WT
pir1Δ
tgs1Δ pir1Δ
ccr4Δ pir1Δ
ccr4Δ tgs1Δ pir1Δ
h
P <= 0.001
Genome-wide comparison of Mmi1 targets
20
81
50
200
282
105
mmi1Δ
ccr4Δ
pir1Δ
ccr4Δ pir1Δ
g
pir1Δ
ccr4Δ
ccr4Δ pir1Δ
Expression relative to WT
log_2FC
mcp5
mug45
mei4
rec8
ccr4Δ
pir1Δ
ccr4Δ pir1Δ
Expression (rpm)
899
3639
1474
2569
Chromosome position (kb)
1.9e-10
3.1e-10
7.6e-07
log_2FC
i
P <= 0.001
Genome-wide targets
685
1217
463
ccr4Δ pir1Δ
tgs1Δ pir1Δ
j
Tgs1-GFP
pir1Δ:
mmi1+
mmi1Δ
kDa
Tgs1-GFP
51
Cdc2
39
k
BPJ4664.02
CC584.15c
dis1
bfr1
AC32A11.02c
mdh1
meu10
cum1
CPB16A4.06c
ppk8
CC569.09
cta3
hsp16
AC23C11.06c
BC17D11.03c
caf5
inv1
CC16A11.15c
osr2
gst4
mug147
pdc201
mpe1
isp7
BC215.11c
hry1
CC736.13
BC19C7.04
osr1
AC19G12.09
trr1
srx1
BC16A3.02c
pbi2
isp6
mam301
CC663.08c
gpd1
cpy1
AC27D7.11c
gst2
AC750.01
snz1
plr1
zwf1
BC1271.08c
hsp9
AC977.14c
obr1
gpd3
ncRNA.530
omt3
ncRNA.1236
CC1919.05-as-2
Expression
tgs1Δ
Tgs1-GFP RIP
mmi1 Δ
+
mRNA
ncRNA
as
log_2FC
Normalized RIP

Supplementary Fig. 5 | TEaM functions in parallel with the MTREC and CPF, but epistatically with CCR4-NOT, to silence gametogenic genes. a, Heatmap showing gametogenic transcript levels in the indicated mutant strains, relative to WT. **b**, **c**, Violin Plots showing the log_2FC expression of gametogenic genes (n = 21) in the indicated strains. The box-plot in red gives the interquartile ranges (25%-75% percentile) with the median marked by a horizontal line in the middle. *P*-values are estimated using the paired 2-tailed t-tests. **d,** Heatmap showing gametogenic transcript levels in the indicated mutant strains, relative to WT (left). Normalized RNA-seq expression profiles showing gametogenic transcript levels across *tgs1Δ*, *ssu72Δ* and *tgs1Δ ssu72Δ* double mutant (middle). Violin Plots showing the log_2FC expression of gametogenic genes (n = 21) in the indicated strains. *P*-values are estimated using the paired 2-tailed t tests (right). **e**, Euler diagram showing the overlap of Mmi1 regulon transcripts (n = 753) with transcripts upregulated in *tgs1Δ*, *ssu72Δ* and *tgs1Δ ssu72Δ* double mutant (fold change, FC>=2). *P*-values indicate significance. **f**, Heatmap showing gametogenic transcript levels in the indicated mutant strains, relative to WT (left). Fold upregulation of gametogenic transcripts over WT in the indicated strains as determined by RT-qPCR, normalized to *act1*$^+$. Position of oligos used are indicated by the red line. Data are presented as mean ± SD; n = 3 independent experiments. *P* values are determined by One Way Anova with Tukey's multiple comparisons (right). **g**, Heatmap showing gametogenic transcript levels in the indicated mutant strains, relative to WT (left). Normalized RNA-seq expression profiles showing gametogenic transcript levels across *ccr4Δ*, *pir1Δ* and the *ccr4Δ pir1Δ* double mutant strains (middle). Violin Plots showing the log_2FC expression of gametogenic genes (n = 21) in the indicated strains. *P*-values are estimated using the paired 2-tailed t-tests (right). **h**, Euler diagram showing the overlap of Mmi1 regulon and transcripts upregulated in the *ccr4Δ pir1Δ* double mutant. *P* value for the 4-way overlap was calculated as decribed in Methods. **i**, Euler diagram showing the overlap of upregulated transcripts (Fold change >=2) in *ccr4Δ pir1Δ* and *tgs1Δ pir1Δ* double mutants. *P* value <= 0.001 indicates significance of overlap. **j**, Immunoblot of Tgs1-GFP levels in *pir1Δ* cells with or without Mmi1. These are strains used for Tgs1-GFP RIP-seq. **k**, Heatmap showing Tgs1-GFP enrichment (RIP-seq) on non-gametogenic transcripts in the presence and absence of Mmi1. Levels of the corresponding transcripts - mRNA, non-coding RNA (ncRNA) and antisense (as) in *tgs1Δ* are shown as heatmap on the right. The *mmi1Δ* strain used in this study also carried a truncation of mei4 (*mei4ΔC*). **a-k**, Experiments were performed in two independent biological replicates with similar results. Source data are provided as a source data file.

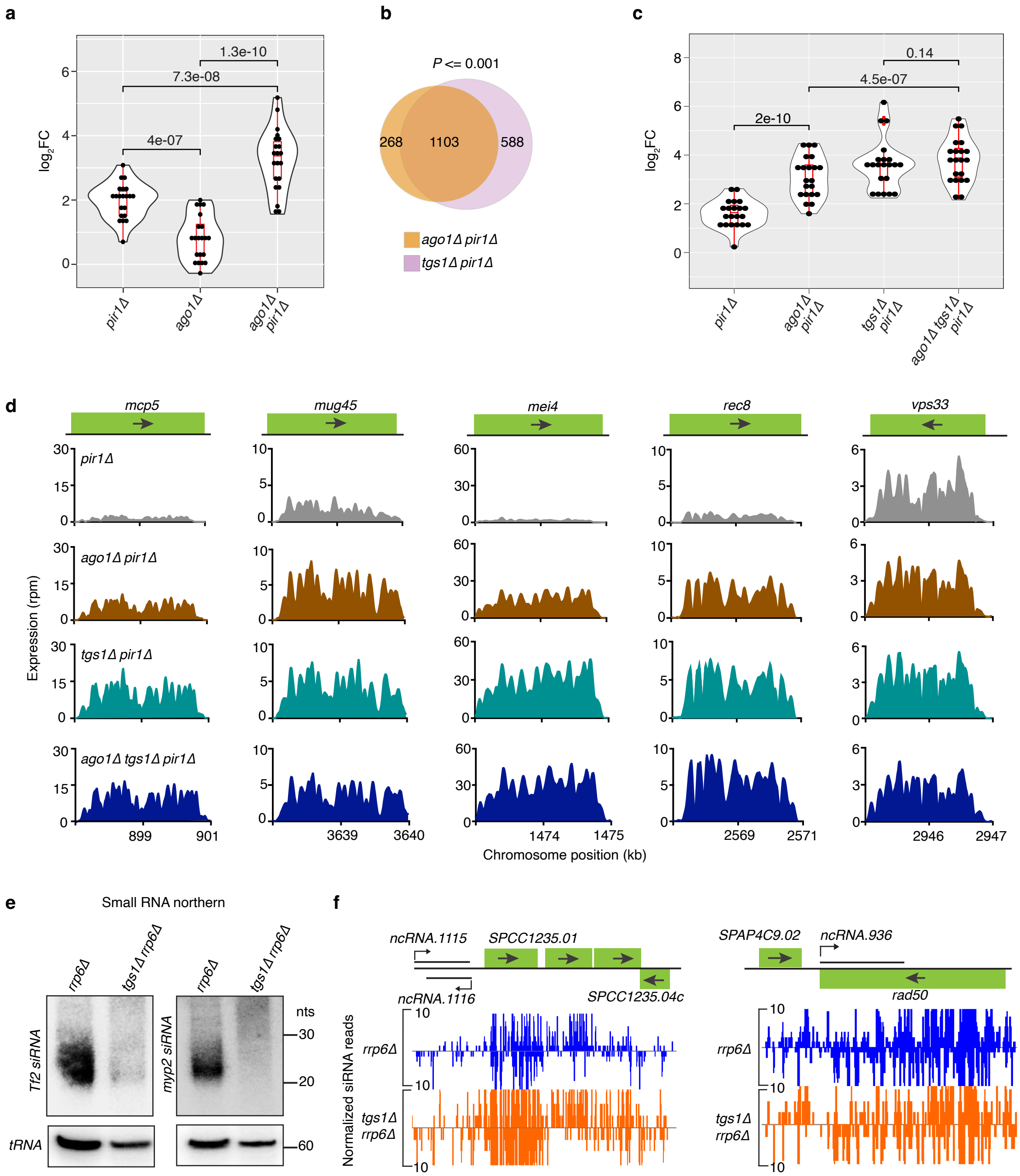

Supplementary Fig. 6 | Tgs1 drives RNAi-mediated silencing of target transcripts. a, Violin plots showing the log_2FC expression of gametogenic genes (n = 21) in the indicated strains. The box-plot in red gives the interquartile ranges (25%-75% percentile) with the median marked by a horizontal line in the middle. *P*-values are estimated using the paired 2-tailed t tests. **b**, Euler diagram showing the overlap of upregulated transcripts (Fold change >=2) in *ago1Δ pir1Δ* and *tgs1Δ pir1Δ* double mutants. *P*-value <=0.01 shows the significance of overlap. **c**, Violin plots showing the log_2FC expression of gametogenic genes (n = 21) in the indicated strains. *P*-values are estimated using the paired 2-tailed t tests. **d,** Normalized RNA-seq expression profiles of gametogenic transcripts in the indicated strains. *vps33* serve as a control locus. **e,** Northern blot analysis of siRNAs from *Tf2* and *myp2* loci in *rrp6Δ* and *tgs1Δ rrp6Δ* cells. **f,** Normalized profile of siRNAs mapping in indicated strains. Data are representative of two independent experiments. **a-f**, Experiments were performed in two independent biological replicates with similar results. Source data are provided as a source data file.

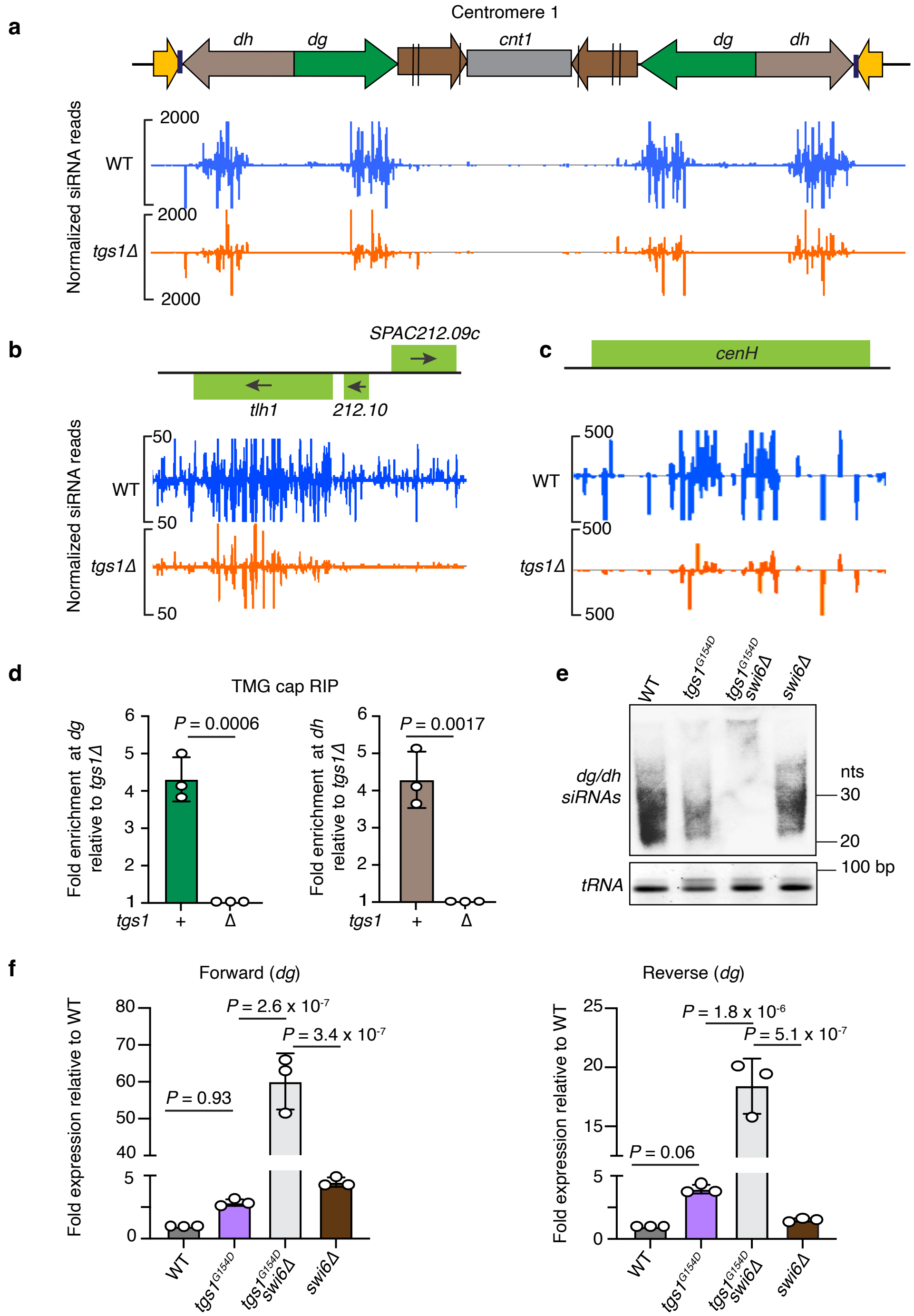

Supplementary Fig. 7 | Tgs1 affects production of siRNAs mapping to repeat elements present at pericentromeric regions, subtelomeres and the silent *mat* region. a, **b**, **c**, Normalized siRNA reads mapping to the pericentromeric repeats (**a**), *tlh1* element at subtelomeric regions of chromosome 1 (**b**) and *cenH* element present at silent *mat* region (**c**) in WT and *tgs1Δ* cells. **a-c**, Data are representative of two independent experiments. **d**, Enrichment of *dg* and *dh* transcripts in TMG-RIP samples analyzed by RT-qPCR. The *swi6Δ* background was used to enable centromeric transcript detection. Fold enrichment is relative to *tgs1Δ*. Data are presented as mean ± SD; n = 3 independent experiments; *P* values were determined by two-tailed unpaired t tests. **e**, Northern blot analysis of centromeric siRNAs in the indicated strains. tRNA levels serve as the loading control and were assessed by agarose gel electrophoresis of 10% of the input RNA from each sample. **f**, Fold upregulation of *dg* transcripts from forward and reverse strands over WT, as determined by RT-qPCR. *act1*⁺ was used for normalization. Data are presented as mean ± SD; n = 3 independent experiments; *P* values were determined by One Way ANOVA with Tukey's multiple comparisons test. Source data are provided as a source data file.

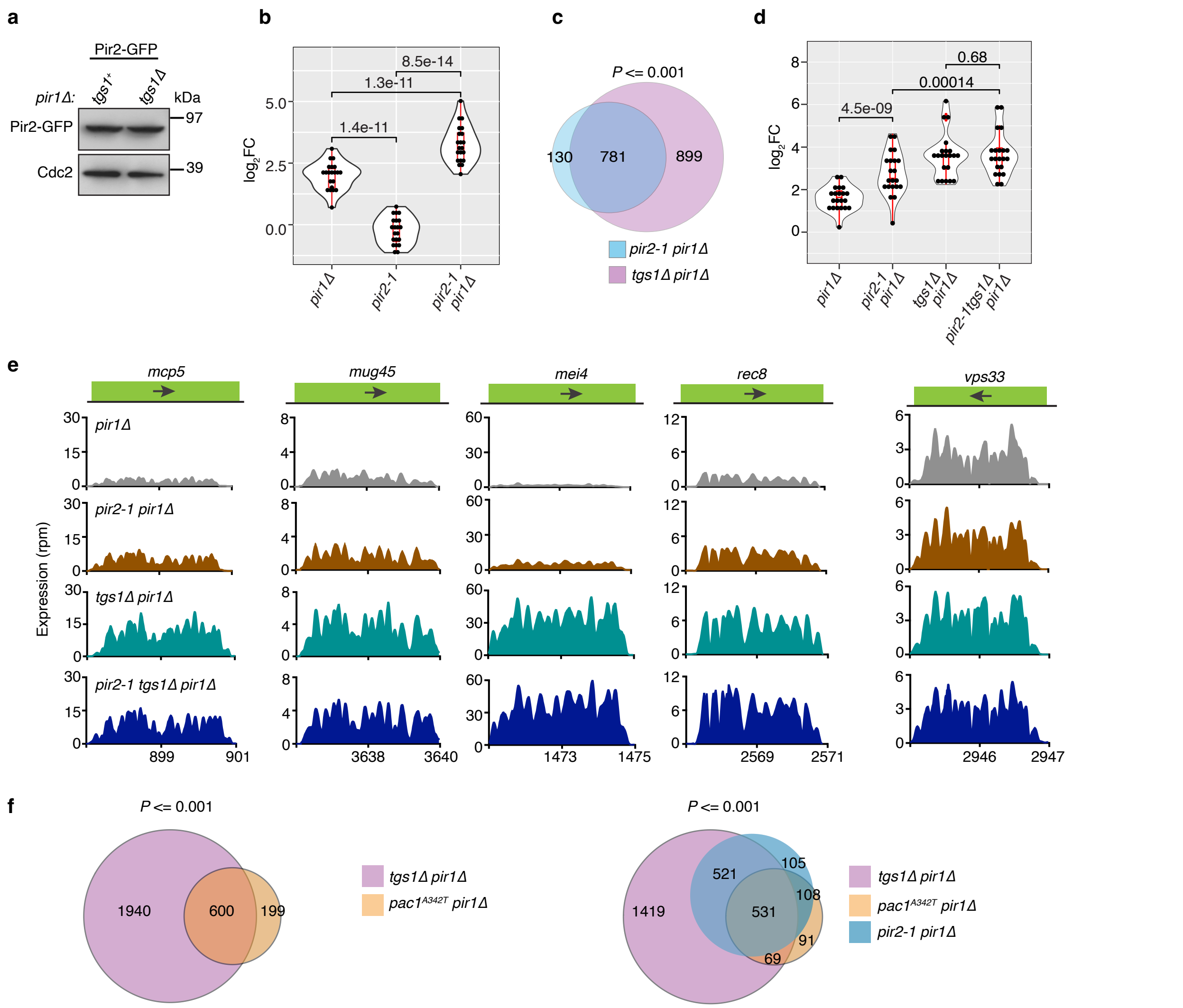

Supplementary Fig. 8 I Pir2 acts in the same pathway as Tgs1 in gametogenic gene silencing. a, Immunoblot of Pir2-GFP levels in *pir1Δ* cells with or without Tgs1**.** These are the same strains used in RIP-seq of Pir2-GFP**. b,** Violin plots showing the $\log_2$FC expression of gametogenic genes (n = 21) in the indicated strains. The box-plot in red gives the interquartile ranges (25%-75% percentile) with the median marked by a horizontal line in the middle. *P*-values are estimated using paired 2-tailed t-tests. **c,** Euler-diagram showing overlap in total upregulated (fold change, FC >=2) transcripts in *pir2-1 pirΔ and tgs1Δ pir1Δ. P* <= 0.001 indicates significance of overlap. Data are representative of two independent experiments. **d**, Violin Plots showing the $\log_2$FC expression of gametogenic genes (n = 21) in the indicated strains. *P*-values are estimated using the paired 2-tailed t-tests. *pir1Δ* and *tgs1Δ pir1Δ* is the same as in Suplementary Fig. 6c, d as samples were part of the same RNA-seq run. **e**, RNA-seq profiles of gametogenic transcripts in the indicated strains illustrated as normalized expression. *vps33* serve as a control locus. **f**, Euler-diagram showing overlap in total upregulated (fold change, FC >=1.5) transcripts in *tgs1Δ pirΔ and pac1^{A342T}pir1Δ* (left) and *tgs1Δ pirΔ, pir2-1 pir1Δ and pac1^{A342T}pir1Δ* (right). *P* <= 0.001 indicates significance of overlap. **a-f**, Experiments were performed in two independent biological replicates with similar results. Source data are provided as a source data file.

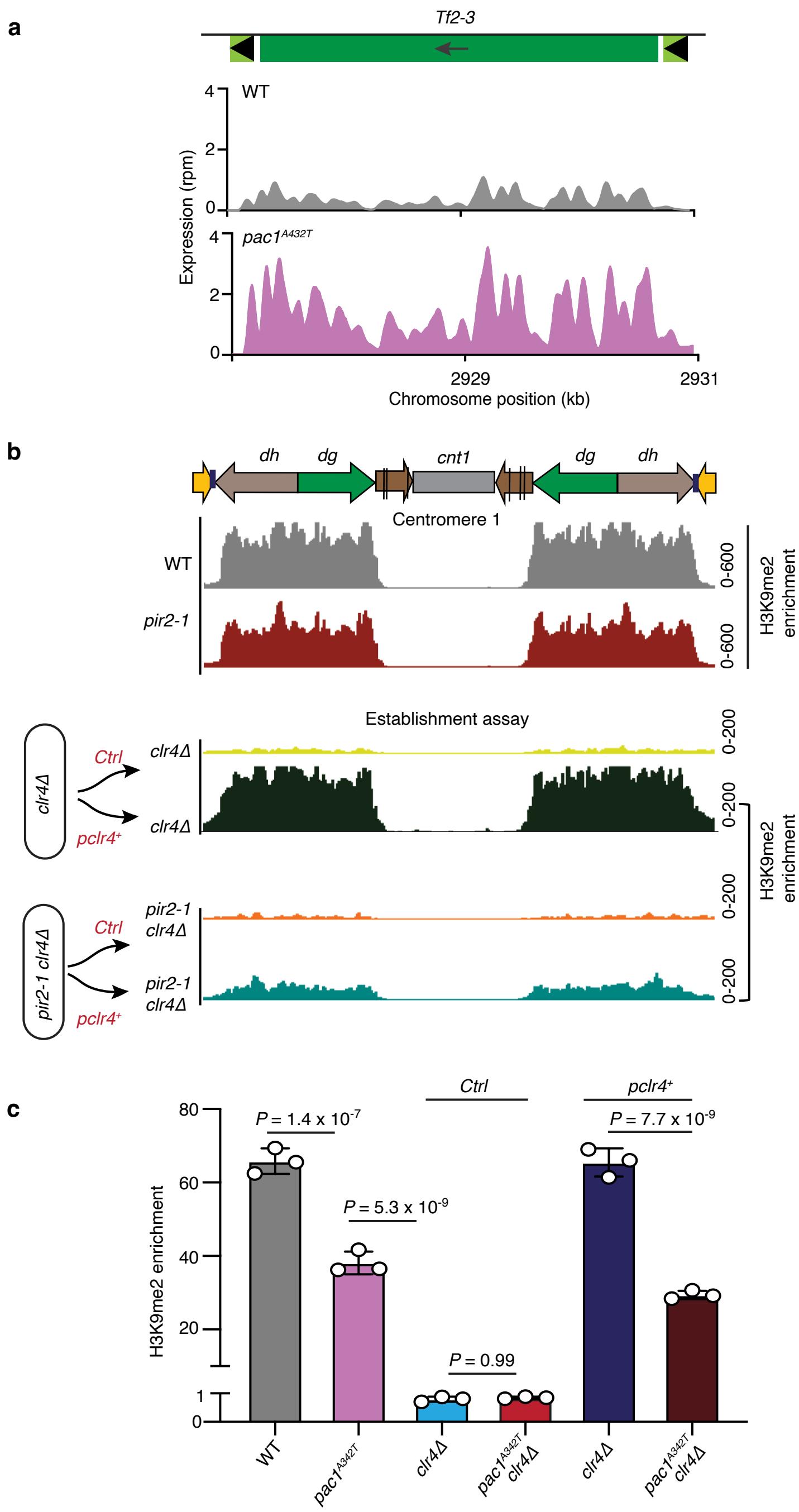

Supplementary Fig. 9 | Pac1, like Pir2, is required for *Tf2* retrotransposon silencing and de novo heterochromatin assembly at centromeric repeats. a, Normalized RNA seq expression profile of *Tf2* in WT and *pac1*^{A342T} strains. Data are representative of two independent RNA-seq experiments. **b,** ChIP-seq profile of H3K9me2 enrichment in the indicated strains. To assess heterochromatin establishment, H3K9me2 profiles were mapped following the reintroduction of a *clr4*$^{+}$-expressing (*pclr4*$^{+}$) plasmid into both *clr4Δ* and *pir2-1 clr4Δ* mutant cells. Empty vector is used as the negative control. **c**, Fold H3K9me2 enrichment at pericentromeric *dg* repeats normalized to WT, analyzed by ChIP-qPCR in the indicated strains. *leu1*$^{+}$ was used as the internal qPCR control. Data are presented as mean ± SD; n = 3 independent experiments. *P*-values are determined by one-way ANOVA followed by Tukey's multiple comparisons test. Source data are provided as a source data file.

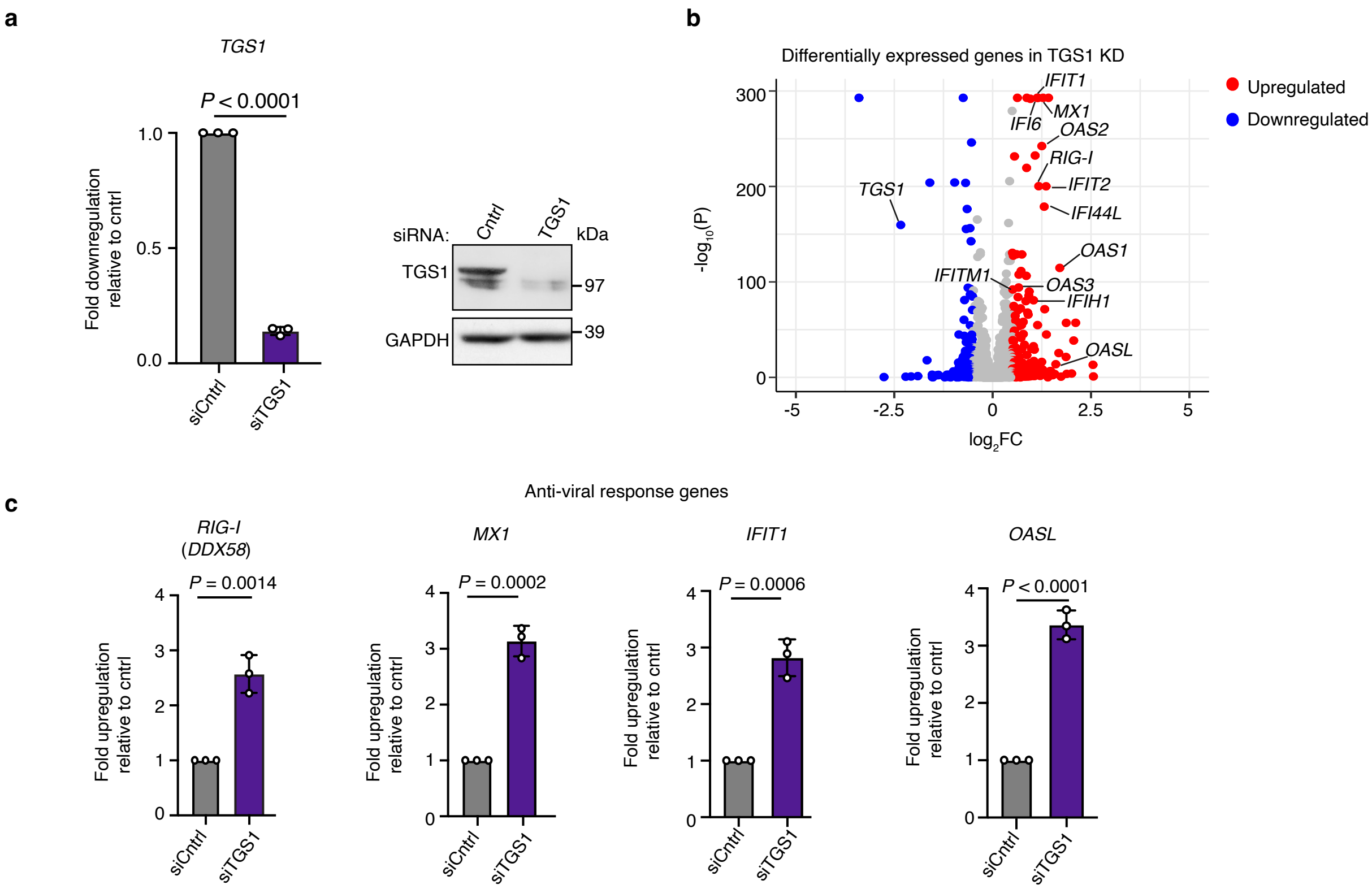

d

Transcript	Category	Function
RIGI	RIG-I pathway	Core sensor, cytosolic RNA helicase
IFIH1	RIG-1 pathway	Cytosolic RNA sensor MDA5; partner to RIG-I in RLR signalling
IFI6	Interferon stimulated gene (ISG), downstream of RIG-I	Interferon-induced, anti-apoptotic/antiviral protein
IFIT1	Interferon stimulated gene (ISG), downstream of RIG-I	Interferon-induced, restricts translation of viral RNAs
MX1	Interferon stimulated gene (ISG), downstream of RIG-I	Interferon-induced, Dynamin-like GTPase; blocks replication of many RNA viruses
IFIT2	Interferon stimulated gene (ISG), downstream of RIG-I	Interferon-induced, works with IFIT1/3 to inhibit viral translation
IFI44L	Interferon stimulated gene (ISG), downstream of RIG-I	Interferon-induced, antiviral
IFITM1	Interferon stimulated gene (ISG), downstream of RIG-I	Restricts viral entry in endosomes, Interferon-induced transmembrane protein
OAS1	OAS pathway	Core 2'-5'oligoadenylate synthetase enzyme
OAS2	OAS pathway	2'-5'-oligoadenylate synthetase, produces 2-5A to activate RNase L
OAS3	OAS pathway	High-affinity dsRNA sensor within OAS family
OASL	OAS pathway	OAS-like, lacks catalytic OAS activity but acts as a interferon stimulated antiviral effector; usually grouped with OAS genes

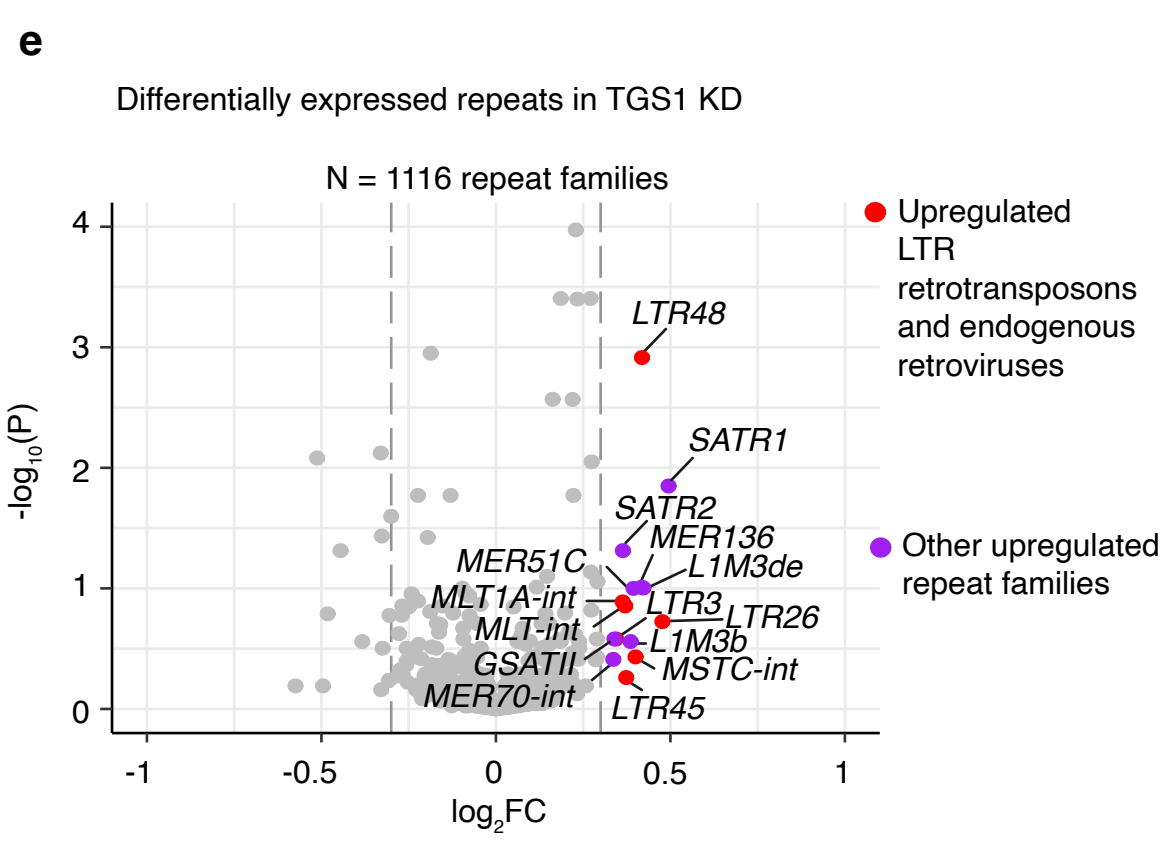

f

Repeat	Category
LTR48	LTR retrotransposon belonging to Endogenous Retrovirus (ERV/ERVL)
MLT1A-int	LTR retrotransposon belonging to Endogenous Retrovirus (ERV/ERVL)
MLT-int	LTR retrotransposon belonging to Endogenous Retrovirus (ERV/ERVL)
LTR26	LTR retrotransposon belonging to Endogenous Retrovirus (ERV/ERVL)
LTR3	LTR retrotransposon belonging to Endogenous Retrovirus (ERV/ERVL)
MSTC-int	LTR retrotransposon belonging to Endogenous Retrovirus (ERV/ERVL)
LTR45	LTR retrotransposon belonging to Endogenous Retrovirus (ERV/ERVL)
L1M3de	LINE-1 Retrotransposon Subfamily
L1M3b	LINE-1 Retrotransposon Subfamily
MER136	DNA Transposon Derived Repetitive Element
MER51C	DNA Transposon Derived Repetitive Element
MER70-int	DNA Transposon Derived Repetitive Element
SATR1	Satellite DNA Repeat Sequence
SATR2	Satellite DNA Repeat Sequence
GSATII	Centromeric/Pericentromeric Satellite DNA Repeat

Supplementary Fig.10 | TGS1 knockdown in human cells leads to upregulation of transposon derived repeats and anti-viral response genes. a, RT-qPCR analysis showing TGS1 transcript downregulation in cells transfected with siRNA against TGS1 (siTGS1) when compared to control siRNA (siCntrl). **b**, Volcano plot showing transcripts de-regulated in cells transfected with siTGS1 when compared to siCntrl. Red dots - upregulated, blue - downregulated, grey - not majorly affected. Representative anti-viral response pathway transcripts that are upregulated in TGS1 knockdown (siTGS1) are labelled. *P* value cut-off applied is <= 0.001. Data are representative of two independent RNA-seq experiments. **c**, RT-qPCR analysis of the indicated genes in cells transfected with siCntrl or siTGS1, n = 3. *P* values are determined by two-tailed unpaired t test. **d**, Table showing anti-viral response genes upregulated in TGS1 knockdown based on their category and function in anti-viral response. **e**, Volcano plot showing $\log_2$FC expression of repeat families in siTGS1 relative to siCntrl cells. Upregulated repeat families are highlighted as red and purple dots. Red dots- LTR retrotransposons and endogenous retroviruses. Purple dots- other upregulated repeat families. Data are representative of two independent RNA-seq experiments. **f**,Table showing repeat elements upregulated in TGS1 knockdown categorized based on their family. Source data are provided as a source data file.

Uncropped Blots

Supplementary Fig. 1c

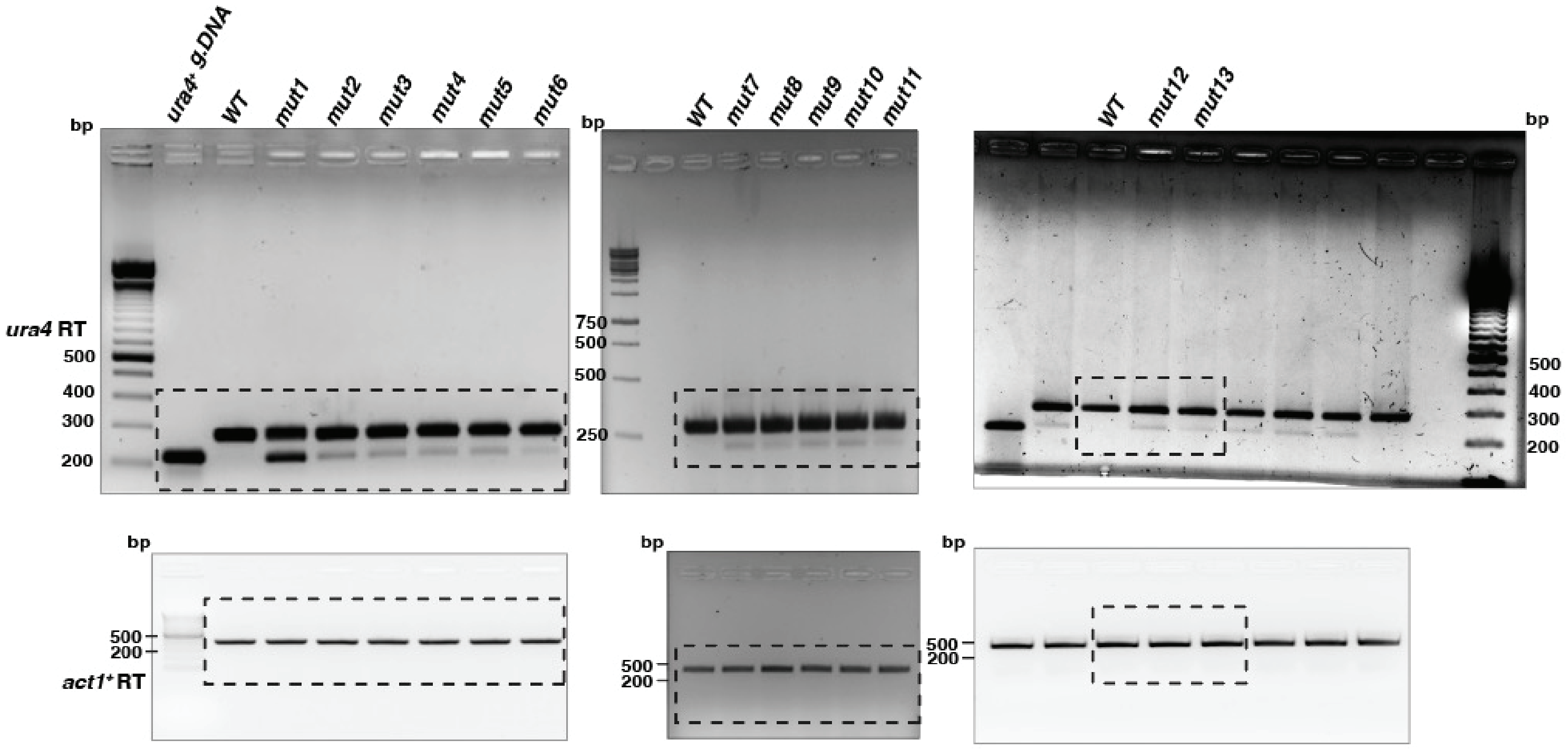

Supplementary Fig. 3b

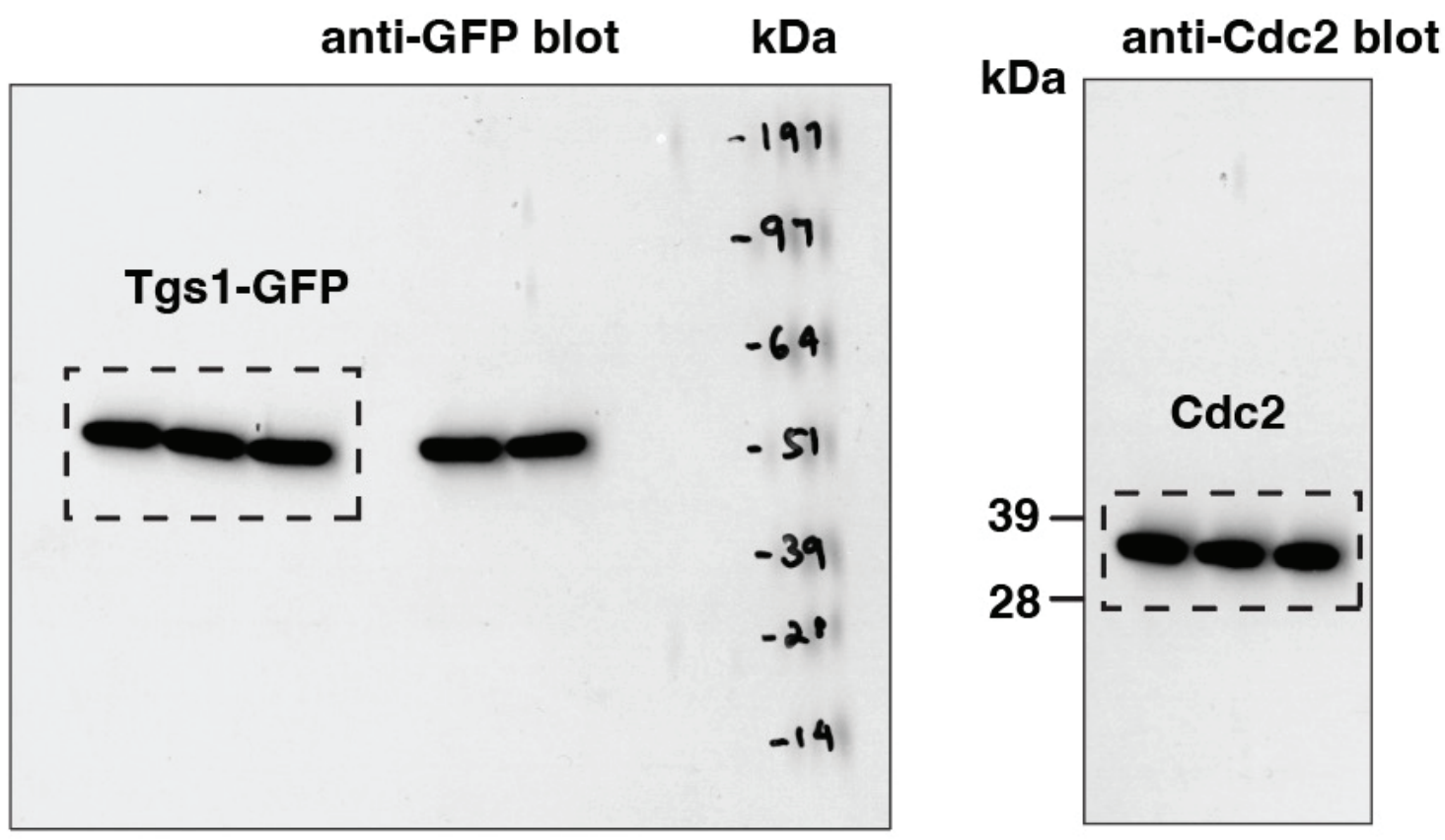

Supplementary Fig. 4c

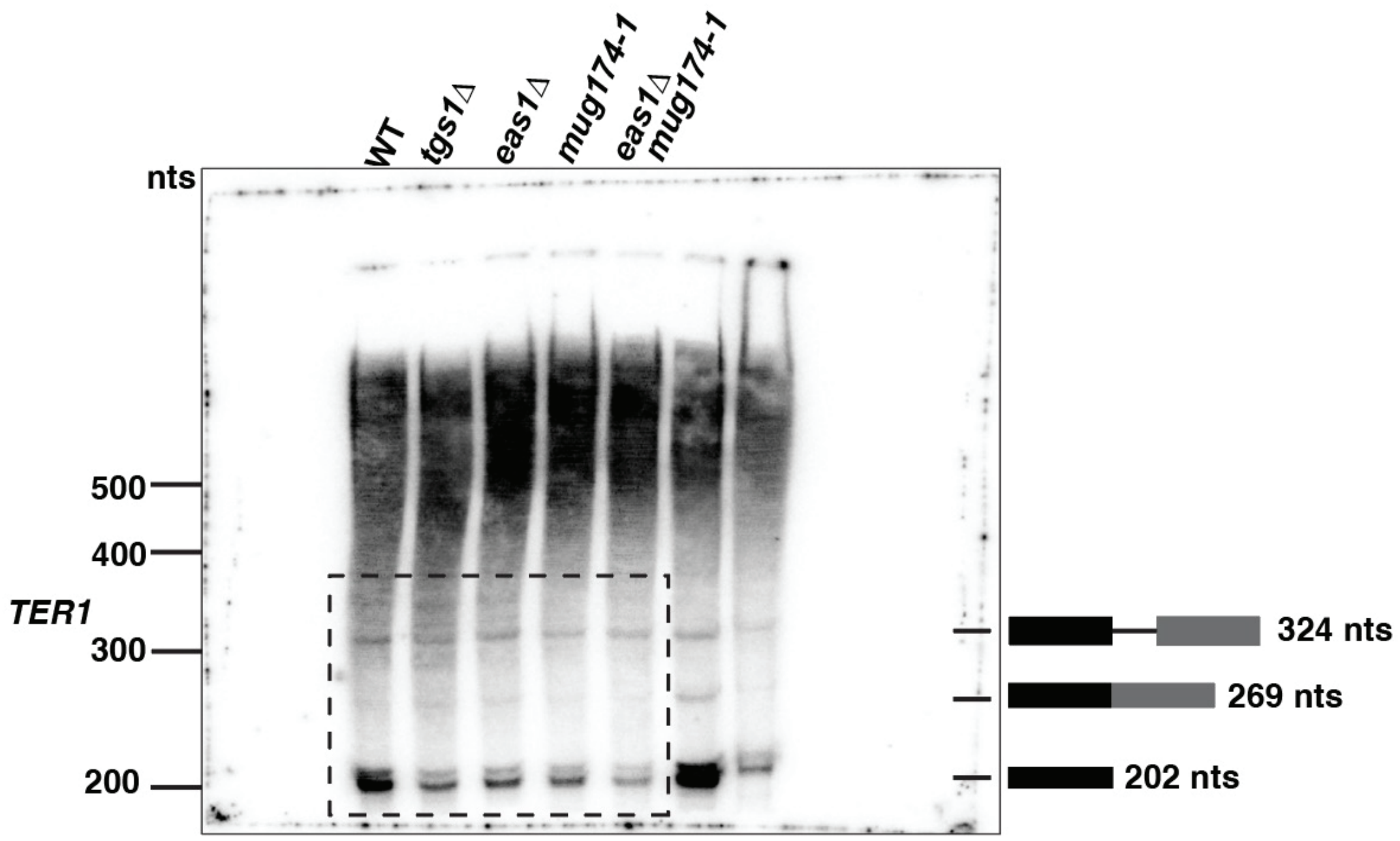

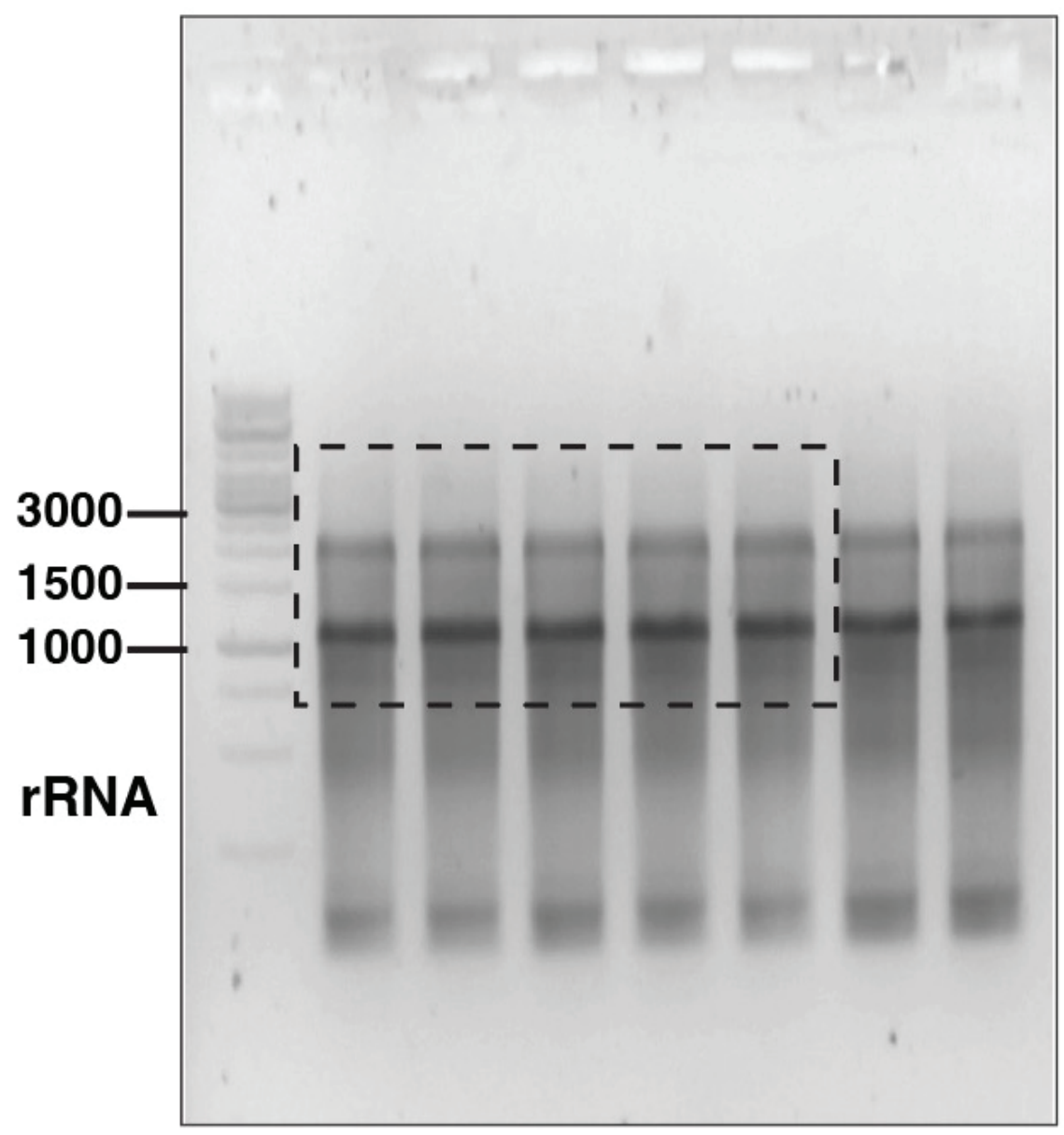

Supplementary Fig. 5j

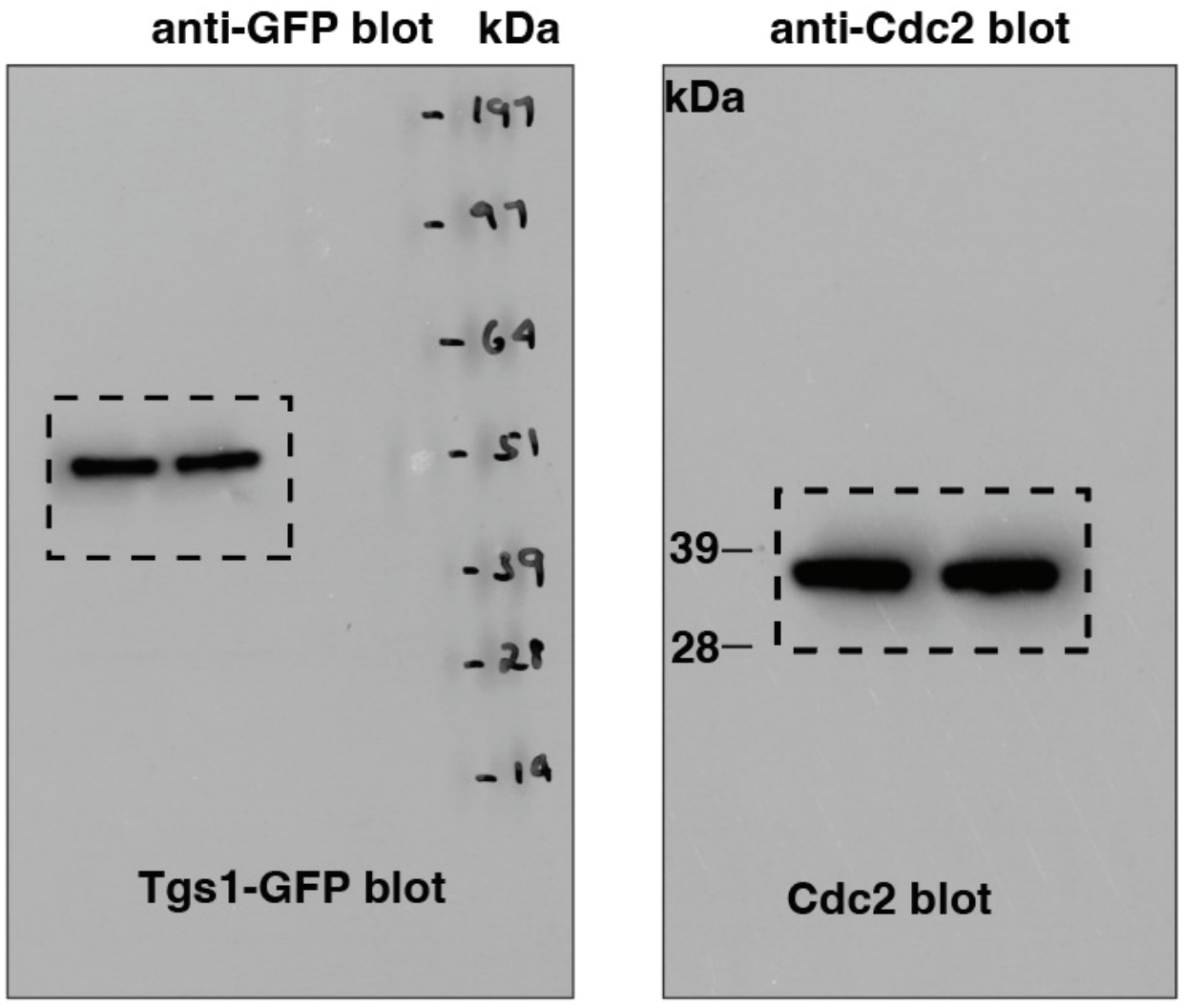

Supplementary Fig. 6e

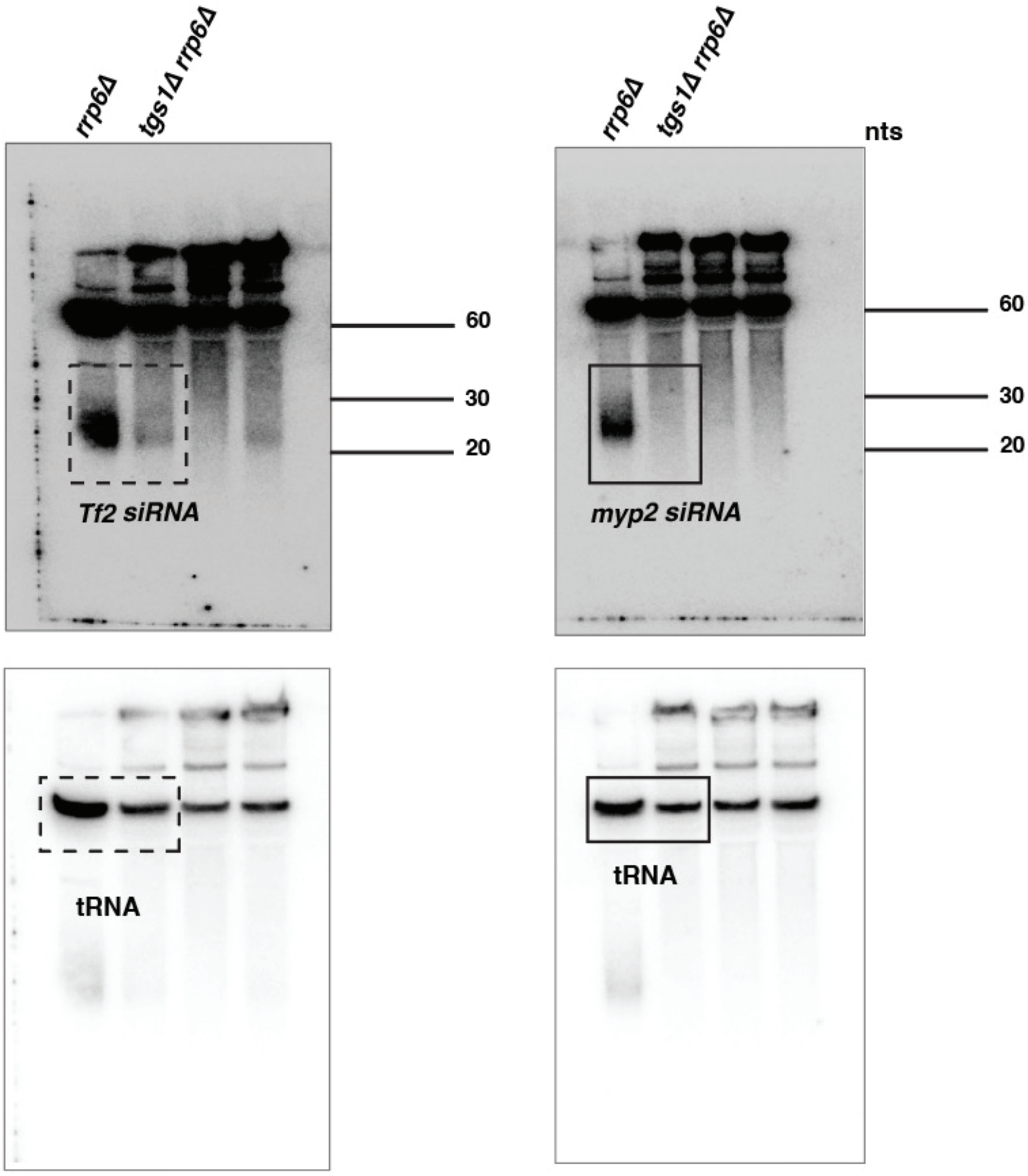

Supplementary Fig. 7e

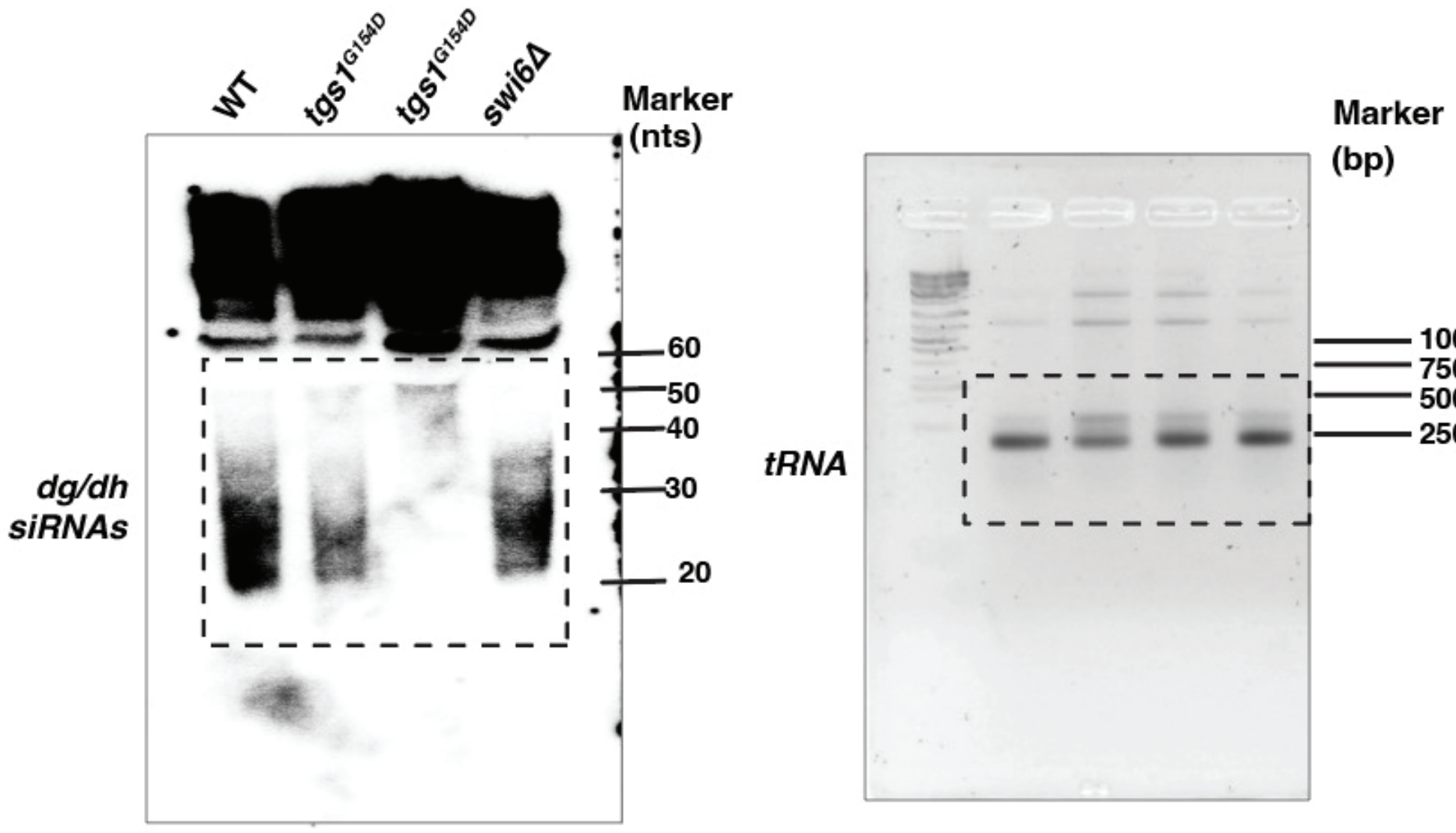

Supplementary Fig. 8a

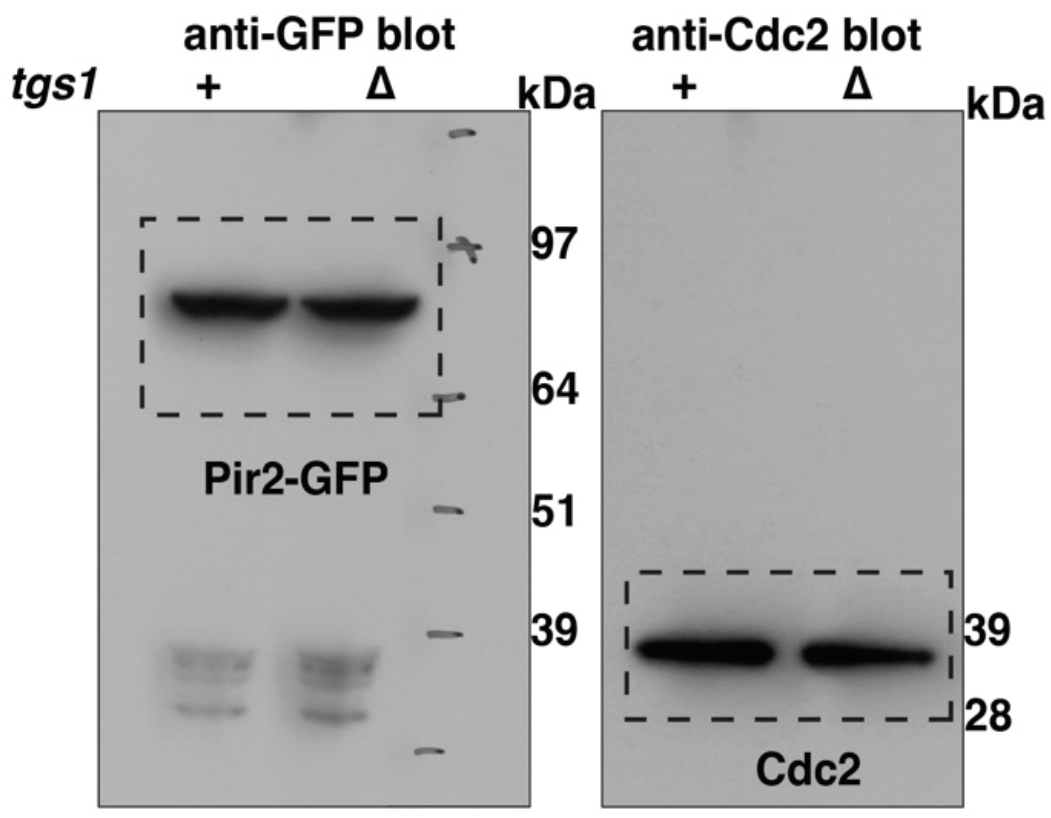

Supplementary Fig. 10a

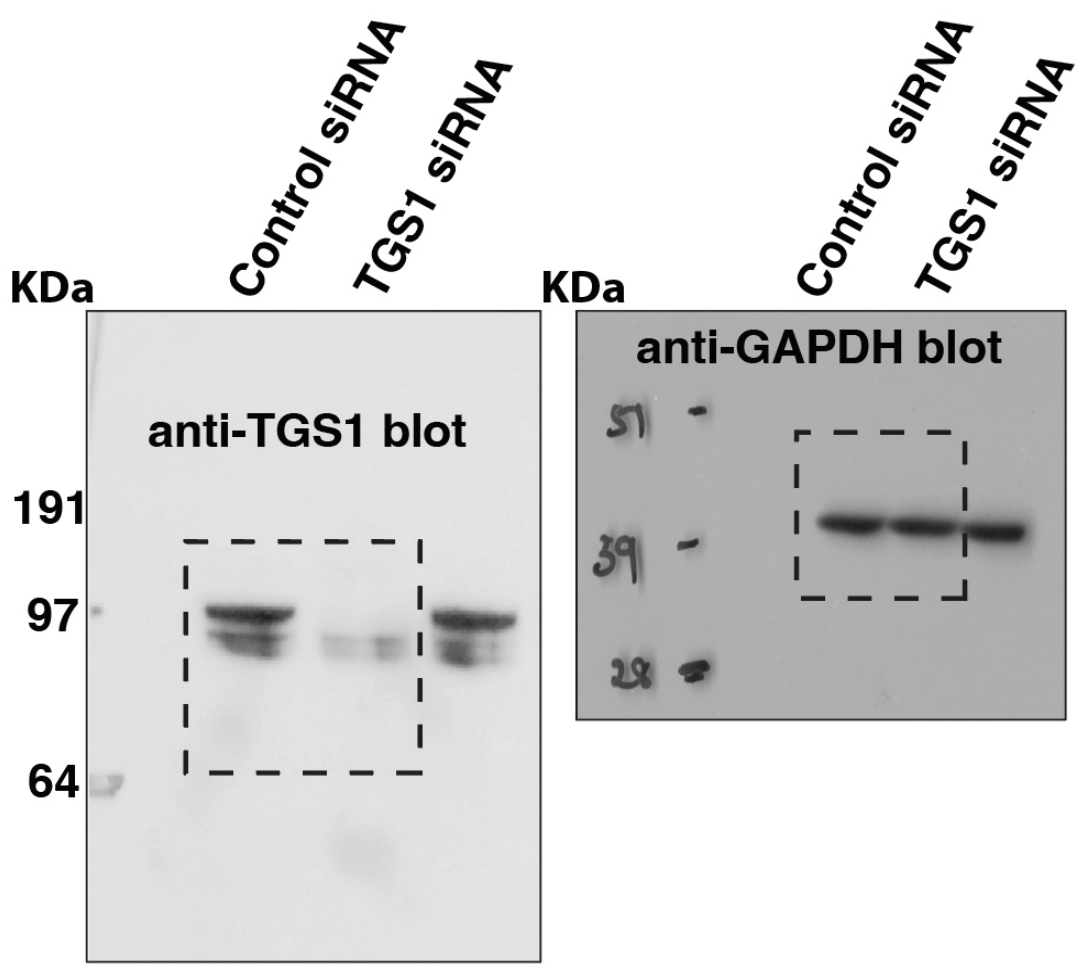

Supplement: Supplementary file 1 [file 41467_2026_75362_MOESM1_ESM.pdf]
