# Supplementary material for: Spliceosomal proteins direct RNA methylation to modulate gene expression and silence retrotransposons
Source: Nat Commun. 2026 Jul 8;17:8405. doi: 10.1038/s41467-026-75362-5 (PMC13473130; doi:10.1038/s41467-026-75362-5)
Supplement: Supplementary file 2 [file 41467_2026_75362_MOESM2_ESM.pdf]

Description of Additional Supplementary Files

File Name: **Supplementary Data 1**

Description: List of factors identified in Mug174 and Eas1 purifications.

File Name: **Supplementary Data 2**

Description: List of cryptic intron containing targets upregulated in *tgs1*Δ.

File Name: **Supplementary Data 3**

Description: Summary of siRNA profile across heterochromatin domains.

File Name: **Supplementary Data 4**

Description: List of *S. pombe* strains used in the study.

File Name: **Supplementary Data 5**

Description: List of oligonucleotides used in this study.
