# Supplementary material for: Spliceosomal proteins direct RNA methylation to modulate gene expression and silence retrotransposons
Source: Nat Commun. 2026 Jul 8;17:8405. doi: 10.1038/s41467-026-75362-5 (PMC13473130; doi:10.1038/s41467-026-75362-5)
Supplement: Supplementary file 3 [file 41467_2026_75362_MOESM8_ESM.pdf]

nature portfolio

Corresponding author(s): Shiv Grewal

Last updated by author(s): 2026/06/25

Reporting Summary

Nature Portfolio wishes to improve the reproducibility of the work that we publish. This form provides structure for consistency and transparency in reporting. For further information on Nature Portfolio policies, see our Editorial Policies and the Editorial Policy Checklist.

Statistics

For all statistical analyses, confirm that the following items are present in the figure legend, table legend, main text, or Methods section.

n/a	Confirmed	
☐	☒	The exact sample size (*n*) for each experimental group/condition, given as a discrete number and unit of measurement
☐	☒	A statement on whether measurements were taken from distinct samples or whether the same sample was measured repeatedly
☐	☒	The statistical test(s) used AND whether they are one- or two-sided<br>*Only common tests should be described solely by name; describe more complex techniques in the Methods section.*
☒	☐	A description of all covariates tested
☐	☒	A description of any assumptions or corrections, such as tests of normality and adjustment for multiple comparisons
☐	☒	A full description of the statistical parameters including central tendency (e.g. means) or other basic estimates (e.g. regression coefficient) AND variation (e.g. standard deviation) or associated estimates of uncertainty (e.g. confidence intervals)
☐	☒	For null hypothesis testing, the test statistic (e.g. *F*, *t*, *r*) with confidence intervals, effect sizes, degrees of freedom and *P* value noted<br>*Give P values as exact values whenever suitable.*
☒	☐	For Bayesian analysis, information on the choice of priors and Markov chain Monte Carlo settings
☒	☐	For hierarchical and complex designs, identification of the appropriate level for tests and full reporting of outcomes
☒	☐	Estimates of effect sizes (e.g. Cohen's *d*, Pearson's *r*), indicating how they were calculated

Our web collection on statistics for biologists contains articles on many of the points above.

Software and code

Policy information about availability of computer code

Data collection: Microscopy imaging was done with Delta Vision Elite microscope (Applied Precision, GE Healthcare). RNA-seq, RIP-seq and small RNA-seq data were collected using next generation Illumina sequencer NextSeq 500/550. ChIP-seq data were collected using next generation Illumina sequencer Miseq. Western blot signals were acquired using the Supersignal West Pico PLUS Chemiluminescent substrate (Thermofisher) and Amersham Hyperfilm ECL developed with Konica SRX-101A. Real time PCR was performed on QuantStudio3 (applied biosystems) using iTaq Universal SYBR Green Supermix (BioRad). Northern blot was scanned using Typhoon FLA 9500 phosphor imager (GE Healthcare).

Data analysis: Deconvolution and maximum intensity projection for microscopy images were done using SoftWoRx (http://incelldownload.gehealthcare.com/bin/download_data/SoftWoRx/6.5.2/SoftWoRx.html). All other processing was done using ImageJ2 1.53v.
Heatmaps were produced using ComplexHeatmap (https://www.bioconductor.org/packages/release/bioc/html/ComplexHeatmap.html)
Eulerr diagrams were produced using https://cran.r-project.org/package=eulerr
hg19-RepeatMasker open-4.0.5
Salmon 1.10.1
MACS2 v2.2.7.1
RIP-seq, RNA seq and ChIP-seq data were visualized in Integrated Genome Viewer (IGV_2.17.4).
Small RNA-seq data were visualized in the Integrated Genome Browser (IGB 10.1.0).
RNA seq and RIP seq reads were trimmed with fastp v1.0.1 and aligned with STAR aligner v2.7.11b software to S. pombe V2 reference sequence which is available at https://www.pombase.org/. Variable interval bedgraphs, normalized to counts per million mapped reads and including both uniquely and multi-mapping reads, were generated by STAR and were further processed to produce 10 bp fixed interval versions for purposes of figure construction. Read counts for transcripts were computed from BAMs using Rsubread and differential expression with respect to WT controls was computed using DEGseq (10.18129/B9.bioc.DEGseq).

For RIP seq, read counts for transcripts were computed from the resulting BAM files using Rsubread for both RIP samples and matched untagged controls. Total counts per transcript were normalized to counts per million mapped reads per 1 Kb after which the normalized untagged counts were subtracted from the corresponding normalized Rip counts. Negative values were converted to zero.
For small RNA seq, single ended short reads were quality trimmed with fastp and aligned to the pombe reference using Novoalign (http://www.novocraft.com/products/novoalign). SGR files, whose format allows the simultaneous display of read coverage from both strands, were subsequently created from the Novoalign-generated BAM files. SGRs were either filtered to remove multi-mapping reads or left unfiltered to include multi-mapping reads and subsequently normalized to counts per 10 million mapped reads (CP10M).
ChIP seq reads were trimmed with fastp v1.0.1 and aligned with BWA aligner v0.7.17 software to S. pombe V2 reference sequence which is available at https://www.pombase.org/. MACS2 v2.2.7.1 callpeaks function was used to produces ChIP enrichment Bedgraphs followed by bdgcmp function to compute fold enrichment over input. Integrative Genomics Viewer v2.17.4 was used to plot enrichments along the fission yeast chromosomes.
Serial dilution plates were scanned using Epson Perfection V700 Photo and presented with Adobe Photoshop 22.4.2 and Illustrator 2024.
The statistical tests and statistic parameters including the mean and standard deviation (SD) were calculated and plotted with Prism 10 for Mac (GraphPad Software).

For manuscripts utilizing custom algorithms or software that are central to the research but not yet described in published literature, software must be made available to editors and reviewers. We strongly encourage code deposition in a community repository (e.g. GitHub). See the Nature Portfolio guidelines for submitting code & software for further information.

Data

Policy information about availability of data

All manuscripts must include a data availability statement. This statement should provide the following information, where applicable:

- Accession codes, unique identifiers, or web links for publicly available datasets
- A description of any restrictions on data availability
- For clinical datasets or third party data, please ensure that the statement adheres to our policy

The RNA-seq , RIP-seq, small RNA-seq and ChIP-seq data reported in this paper are available at Gene Expression Omnibus (https://www.ncbi.nlm.nih.gov/geo/) with accession number GSE300777 (https://www.ncbi.nlm.nih.gov/geo/query/acc.cgi?acc=GSE300777); GSE300779 (https://www.ncbi.nlm.nih.gov/geo/query/acc.cgi?acc=GSE300779); GSE300776 https://www.ncbi.nlm.nih.gov/geo/query/acc.cgi?acc=GSE300776); GSE300775 (https://www.ncbi.nlm.nih.gov/geo/query/acc.cgi?acc=GSE300775respectively. Mass-spec data are available at MassIVE database with accession number MSV000100022.
Source data are provided with this paper in the Source Data file.
Raw images of WB, NB and gels are provided in the corresponding Source Data file with this paper.
S. pombe ASM294v2.30 reference sequence is available at https://www.pombase.org/.

Research involving human participants, their data, or biological material

Policy information about studies with human participants or human data. See also policy information about sex, gender (identity/presentation), and sexual orientation and race, ethnicity and racism.

Reporting on sex and gender	N/A
Reporting on race, ethnicity, or other socially relevant groupings	N/A
Population characteristics	N/A
Recruitment	N/A
Ethics oversight	N/A

Note that full information on the approval of the study protocol must also be provided in the manuscript.

Field-specific reporting

Please select the one below that is the best fit for your research. If you are not sure, read the appropriate sections before making your selection.

☒ Life sciences ☐ Behavioural & social sciences ☐ Ecological, evolutionary & environmental sciences

For a reference copy of the document with all sections, see nature.com/documents/nr-reporting-summary-flat.pdf

Life sciences study design

All studies must disclose on these points even when the disclosure is negative.

Sample size	Sample size selection was based on experience from prior studies: [Sahu et al., 2024, Mol Cell (PMID: 39096900); Wei et al., 2021, Nat Cell Biol (PMID: 33574613); Holla et al., 2020, Cell (PMID: 31883795); Mazza et al., 2013, Methods Mol Biol (PMID: 23980004)
Data exclusions	No data were excluded from analyses.
Replication	For all experiments, at least two biological replicates were performed. All attempts at replication were successful. The number of replicates is indicated in the figure legends.

Randomization	Randomization was not relevant to the assays conducted in this study. All cultures were grown under strictly controlled conditions and collected without any subjective selection.
Blinding	Investigators were not blinded during this study; experimental conditions were strictly controlled, and proper controls utilized. No subjective group allocation was required.

Reporting for specific materials, systems and methods

We require information from authors about some types of materials, experimental systems and methods used in many studies. Here, indicate whether each material, system or method listed is relevant to your study. If you are not sure if a list item applies to your research, read the appropriate section before selecting a response.

Materials & experimental systems

n/a	Involved in the study
☐	☒ Antibodies
☐	☒ Eukaryotic cell lines
☒	☐ Palaeontology and archaeology
☒	☐ Animals and other organisms
☒	☐ Clinical data
☒	☐ Dual use research of concern
☒	☐ Plants

Methods

n/a	Involved in the study
☐	☒ ChIP-seq
☒	☐ Flow cytometry
☒	☐ MRI-based neuroimaging

Antibodies

Antibodies used	RIP and ChIP experiments were performed by combining 1 ml of pre-cleared cell lysate with appropriate antibody:<br>For RIP, 2.5 µg of anti-GFP (Abcam, Catalog#ab290) was used. For TMG RIP, 10 µg of anti-2,2,7-trimethylguanosine (m3G/TMG) mAb (MBL Life Science, Catalog#RN019M, clone 235-1) was used. For ChIP, 2.5 µg of anti-H3K9me2 (Abcam, Catalog#ab115159) was used. Immunoprecipitation experiments were performed with 100 µl of ChromoTek GFP-Trap Agarose (ChromoTek, Catalog#gta-20). Western blot experiments were performed with anti-GFP (Roche, Catalog#11814460001; 1:1000 dilution), anti-HA (Santa Cruz, Catalog# Sc-7392; 1:1000 dilution), anti-c-Myc 9E10 (Santa Cruz, Catalog#sc-40; 1:1000 dilution), anti-FLAG M2 (Sigma Aldrich, Catalog#F3165; 1:1000 dilution) or anti-Cdc2 (Santa cruz, Catalog#sc-53217) at 1:1000 dilution. We used 1:2500 dilution of secondary anti-mouse IgG1 (GE Healthcare, Catalog#NA931) HRP linked antibody. Western blot experiments involving human cell-line extracts were performed with anti-PIMT (Bethyl, A300-814A; 1:1000 dilution) and anti-GAPDH (Abcam, ab8245; 1:1000 dilution). We used 1:2500 dilution of secondary anti-mouse IgG1 (GE Healthcare, Catalog#NA931) and rabbit IgG (GE Healthcare, Catalog#NA934) HRP linked antibody.
Validation	All antibodies used in this study are well-recognized clones. Antibodies have been validated both by the manufacturer and citations provided for the scientific literature. Validation data for each antibody can be accessed on the respective manufacturer's homepage.

Eukaryotic cell lines

Policy information about cell lines and Sex and Gender in Research

Cell line source(s)	Human foreskin fibroblasts BJ CRL-2522 (ATCC)
Authentication	Human foreskin fibroblasts BJ CRL-2522 was frequently checked for their morphological features
Mycoplasma contamination	The cell lines were tested for Mycoplasma contamination by PCR
Commonly misidentified lines (See ICLAC register)	No commonly misidentified cell line was used

Plants

Seed stocks	N/A
Novel plant genotypes	N/A
Authentication	N/A

ChIP-seq

Data deposition

- [x] Confirm that both raw and final processed data have been deposited in a public database such as GEO.
- [x] Confirm that you have deposited or provided access to graph files (e.g. BED files) for the called peaks.

Data access links<br>*May remain private before publication.*	The RNA-seq, RIP-seq, small RNA-seq and ChIP-seq data reported in this paper are available at Gene Expression Omnibus (https://www.ncbi.nlm.nih.gov/geo/) with accession number GSE300777 (https://www.ncbi.nlm.nih.gov/geo/query/acc.cgi?acc=GSE300777); GSE300779 (https://www.ncbi.nlm.nih.gov/geo/query/acc.cgi?acc=GSE300779); GSE300776 (https://www.ncbi.nlm.nih.gov/geo/query/acc.cgi?acc=GSE300776); GSE300775 (https://www.ncbi.nlm.nih.gov/geo/query/acc.cgi?acc=GSE300775) respectively. Mass-spec data are available at MassIVE database with accession number MSV000100022.
Files in database submission	Please see above.
Genome browser session<br>(e.g. UCSC)	N/A

Methodology

Replicates	Atleast two biological replicates were successfully performed for RIP seq, RNA seq and small RNA seq experiments.
Sequencing depth	RNA Seq libraries were sequenced to ~20-50 million reads for S. pombe and ~80-150 million reads for human.<br>RIP Seq libraries were sequenced to ~30-60 million reads.<br>Small RNA Seq libraries were sequenced to ~30-60 million reads.<br>ChIP Seq libraries were sequenced to ~1-3 million reads.
Antibodies	anti-GFP (Abcam, Cat#ab290),<br>anti-H3K9me2 (Abcam, Catalog#ab115159)
Peak calling parameters	--broad, --nomodel, --extsize 147 -g 12.57e6
Data quality	Reads were quality-trimmed using fastp. QC reports are generated by fastp.
Software	fastp v1.0.1(read trimming), STAR aligner v2.7.11b (alignment of reads from RNAseq and generation of RPM-normalized bedgraphs), BWA v0.7.17 (alignment of reads from ChIPseq), MACS2 v2.2.7.1 (generation of fold enrichment bedgraphs of ChIPseq data).
