# Supplementary material for: Spliceosomal proteins direct RNA methylation to modulate gene expression and silence retrotransposons
Source: Nat Commun. 2026 Jul 8;17:8405. doi: 10.1038/s41467-026-75362-5 (PMC13473130; doi:10.1038/s41467-026-75362-5)
Supplement: Supplementary file 4 [file 41467_2026_75362_MOESM9_ESM.pdf]

nature portfolio

Peer Review File

Spliceosomal proteins direct RNA methylation to modulate gene expression and silence retrotransposons

Corresponding Author: Dr Shiv Grewal

This file contains all reviewer reports in order by version, followed by all author rebuttals in order by version.

Version 0:

Reviewer comments:

Reviewer #1

(Remarks to the Author)
This is an impressive and expansive manuscript that describes a new complex of proteins, including tgs (and enzyme that makes trimethylated G caps on RNA) as critical for several aspects of gene silencing, splicing, siRNA production, retrotransposon silencing and other functions. A new complex is described (TeAM).

The question for me is the mechanism. It is appealing that the RNAs are targeted fro trimethyl G capping which targets them for degradation. But the amount of TMG (trimethyl G) caps is not measured quantitatively, in part because there is no clear method to do so. But central to the idea is quantitative conversion of the caps of these mRNAs to TMG. It would be good if the authors can think of ways to address this. The other issue is that the Tgs enzyme also installs TMG caps on snRNAs, and the Tgs enzyme was found to interact with key snRNP components. How do we know that the effects on splicing are not due to impaired TMG formation in the snRNA? I don't think this has been addressed and some evidence is needed to distinguish the contribution of different Tgs targets on the biological effects seen upon Tgs depletion.

Reviewer #2

(Remarks to the Author)
In the current study, Vijayakumari and co-workers Identified trimethylguanosine synthase (Tgs1) in a screen for mutants with enhanced splicing of a cryptic intron present in retrotransposons in the fission yeast S. pombe. They show that these transcripts are modified by trimethylguanosine (TMG), and that Tgs1 recruitment is tightly linked to splicing, as TMG capping depends on an inefficient splice site within the cryptic intron and requires splicing factors. Mass spec and co-IP experiments further reveal that Tgs1 associates with splicing factors and the YTH RNA-binding protein Mmi1. In addition, they identified Mug174 and an unknown protein termed Eas1, mutants of which were also isolated in this screen. All three proteins co-localize to nuclear condensates resembling Cajal bodies and affect each other's localization, consisting with a previous study (Deng et al 2024). Moreover, recruitment of Tgs1 to target genes is impaired in mug174 and eas1 mutants, which show an additive effect. Based on these findings, the authors propose that Tgs1, Eas1 and Mug174 form a physical complex, which they name 'TEaM'.

Beyond retrotransposons, the authors show that Tgs1 contributes to the repression of a broad set of transcripts including non-coding RNAs, stress-related, and gametogenic genes. They propose that this repression is mediated by two alternative pathways involved in Tgs1 recruitment: transcripts containing cryptic splice sites are recognized by splicing factors, whereas transcript harboring DSR (determinant of selective removal) elements are recognized by Mmi1. Mmi1 has previously been shown to target RNAs primarily to the nuclear exosome via its interaction with Pir1 (also known as Iss10), which associates with the central RNA surveillance hub MTREC. In this study, the authors provide evidence that Mmi1 also interacts with Tgs1, resulting in targeting TMG-capped transcripts to the RNAi machinery via Pir2-ARS2, possibly through direct its binding to the TMG structure and recruiting RNAi factors like RNA-dependent RNA polymerase . This RNAi-dependent mechanism also acts on TMG-capped pericentromeric heterochromatic transcripts in parallel to Swi6-dependent siRNA generation, consistent with earlier findings (Yu et al 2021). Finally, the authors demonstrate that retrotransposon repression by Tgs1 is conserved in mammalian cells.

Overall, this study provides new and important insights into the specificity and molecular mechanisms of RNA-targeting pathways that regulate non-coding RNAs, retrotransposons, and developmental genes through splicing-coupled and RNAi-

mediated repression. While some aspects were previously known, such as the association of Tgs1 with the coilin homolog Mug174, their contribution to the pericentromeric heterochromatin assembly, and the role of Pir2-ARS2 in RNAi production, the present work substantially advances the field by integrating these processes into a coherent network of regulatory pathways and by revealing functional connections that had not been fully appreciated. The experiments have been carefully performed, most data are appropriately represented, and the conclusions are, for the most part, well supported by the findings. In particular, the genetic data revealing redundancy among the different pathways are compelling; however, the physiological relevance of the Tgs1/Pir2-dependent RNAi pathway remains unclear, as its contribution is largely detectable only under sensitized conditions (i.e. in the absence of RNA degradation by MTREC-Pir1 or Swi6).

Despite these strengths, the manuscript occasionally presents mechanistic interpretations in a manner that by be overly simplified, most notably in concluding the existence of a physical complex formed by Tgs1 and its associated partners. In this case, critical experiments are lacking, and in the absence of additional data, alternative models should be considered and explicitly discussed. In addition, some data analyses lack appropriate controls (e.g. wild-type conditions), making it difficult to assess epistatic interactions in mutants with weak phenotypes. Finally, several findings that corroborate earlier studies are adequately acknowledged or placed into the context of the existing literature. These issues, together with the specific points outlined below, must be addressed before publication can be recommended.

Major points:

(1) Additional work is necessary to support the claim that Tgs1, Mug174 and Eas1 form a physical complex. At present, direct biochemical evidence is lacking, and several observations are difficult to reconcile in this model:
• The mutant mug174-1 (V32M) only partially recapitulates the splicing phenotypes of tgs1Δ and eas1Δ (Figure 1b, c) and is therefore not well suited for genetic interaction analysis.
• The reported additive phenotypes of mug174-1 and eas1Δ (Figure 2, Ext. Figure 4-5), are inconclusive (see above) and are not readily consistent with a simple model of a stable ternary complex with Tgs1. Conversely, Mug174 condensates are affected differently in eas1Δ and tgs1Δ backgrounds, resulting in multiple foci.
• N-terminal residues (1-103) of Eas1 and Mug174 show high similarity based on structure prediction (Ext. Figure 2e). The N-terminal domain of Mg174 has previously shown to be necessary for interaction with Tgs1, suggesting that both Eas1 and Mu174 interact with Tgs1 via their N-terminal domains and may therefore compete for binding.
• Partially distinct sets of splicing factors co-purify with Mug174 and Eas1 (Figure 2a, b).
• Unlike tgs1Δ and eas1Δ, the complete deletion mutant mug174Δ displays a growth phenotype (not shown but reported in Deng 2024) and also displays quiescence phenotypes that differ from tgs1Δ (Deng 2024), suggesting additional functions, Given the weaker phenotype of eas1Δ relative to tgs1Δ, the additive phenotype with mug174-1, and the predicted structural similarity between Eas1 and Mug174, an alternative model is that Tgs1 forms two independent complexes with Eas1 and Mug174, respectively. Importantly, the presence of Mug174 in Eas1 pulldown/mass spectrometry experiments (and vice versa) does not by itself support a shared complex, as other factors unlikely to be part of such a complex (e.g. Mmi1, Sm proteins, and general splicing factors) are present at comparable levels. To demonstrate that all three proteins can co-exist in a shared complex, the authors should analyze purifications from yeast (or reconstituted recombinant proteins) using SDS page and size exclusion chromatography or density-gradient fractionation, as performed in their previous work (Thillainadesan et al 2020). Otherwise, the conclusion regarding a single physical 'TEaM' complex needs to be substantially toned down.
(2) The authors argue that the absence of additive phenotypes between mutants indicates epistatic interactions and, by extension, that the corresponding factors act within the same pathway. However, expression analyses of selected Mmi1-regulated genes (Ext. Figures 5d, 6b, and 8b) lack comparison wild-type cells. This omission makes it difficult to assess whether the very low gene expression profiles observed in mutants such as tgs1Δ, pir2-1, or ago1Δ represent baseline expression or genuine mutant phenotypes. Epistatic interactions, particularly between weak mutants, can only be meaningful interpreted when a clear phenotype is present. Otherwise, the absence of phenotypes in single and double mutants merely indicate that neither factor contributes clearly under the tested conditions. For a rigorous assessment of epistatic interactions, the authors should analyze these mutants under sensitized conditions where phenotypes are robust, for example by combining them with pir1Δ and comparing the double mutants pir1Δ tgs1Δ and pir1Δ ago1Δ to the corresponding triple mutant pir1Δ tgs1Δ ago1Δ. I would also strongly recommend including control genes or loci that are not affected by these pathways to allow a clearer assessment of specificity.
(3) Several results reported here confirm key findings from previous studies, but these are not sufficiently acknowledged or integrated. For example, the authors report that pericentromeric transcripts are TMG-capped (Figure 6d), that Tgs1 activity contributes to heterochromatin silencing (Figure 6f), that Tgs1 is required for H3K9me maintenance (Figure 6c) and Pir2-1 required for de novo heterochromatin assembly (Ext. Figure 8g), and that Tgs1 acts in parallel to Swi6 in RNAi-dependent heterochromatin silencing at pericentromeres (Figure 6b, c, f). With the exception of Pir2-1, (which largely phenocopies tgs1Δ), these findings were already reported by Yu et al. (2020; PMID: 33527595). Similarly, Deng et al. (2024; PMID: 38828770) demonstrated that Mug174 contributes to pericentromeric heterochromatin assembly. I strongly recommend combining these largely confirmatory data in the supplementary material and reframing them explicitly in the context of the existing literature. Deng et al 20204 further showed that Mug174 interacts and colocalize with Tgs1, that Mug174 foci formation depend on Tgs1, and that snRNA TMG capping requires Mug174, analogous to Tgs1. Although the physical association and functional relationship between Tgs1 and Mug174 were established prior to the present study, this information is marginally discussed in the current manuscript and should be incorporated more explicitly.

Minor points

Text, page 9: "Similar cumulative derepression was observed when tgs1Δ was combined with deletion of ssu72" appears overstated. Although transcript levels in the double mutant are modestly increased relative to the single mutants, the

cumulative derepression (~ 2-3 rpm) is substantially weaker than that observed in tgs1Δ pir1Δ (10-60 rpm). Please consider revising this statement accordingly.
Extended Figure S4 a, b vs. Figure 3a, b: Please clarify whether experiments in Extended Figure 4 a, b were also performed in an abp1 mutant background, as in Figure 3 a, b. This not clear from Results text. More generally, I recommend indicating sensitized strain backgrounds not only in the legend (as currently done) but also directly in the Figures (Figures 3a/Ext. Figure 4a), either in the panels or axis label (e.g. "Tgs1 enrichment in pir1Δ/abp1-DDE strain").
Suppl Figure S3c: mug174-1 should be labeled as mug174-1-GFP (panel 4)
Suppl. Figure S5h: Please clarify whether expression data in the right column were obtained in tgs1Δ or tgs1Δ mmi1Δ double mutants.
mmi1 mutant: According to S. pombe literature, mmi1 is an essential gene, but its essential function can be bypassed by deleting mei2. Please clarify how mmi1Δ experiments have been performed and in which genetic background.

Reviewer #3

(Remarks to the Author)
The study entitled "Spliceosomal proteins direct RNA methylation to modulate gene expression and silence retrotransposons" reports the discovery of a conserved regulatory pathway controlling RNA modification in Schizosaccharomyces pombe. Using a genetic screen based on introducing cryptic introns from the Tf2 retrotransposon, the authors identified mutations that affect splicing efficiency. They showed that Tgs1, which forms the TEaM complex with Eas1 and Mug174, promotes inefficient splicing of these cryptic introns in a dose-dependent manner by catalyzing TMG capping of target transcripts. In addition, the YTH family RNA-binding protein Mmi1 recruits Tgs1 to specifically repress retrotransposons or gametogenic genes harboring cryptic introns, thereby directing RNA methylation–dependent silencing of gametogenic genes and retrotransposons, and triggering RNAi and heterochromatin deposition at target loci. The data thus establish a silencing cascade whereby the cryptic intron splicing machinery directs RNA interference through RNA modification and heterochromatin.

Specific comments:

1. Missing genetic screening details. Although the design of the forward genetic screen with backcross selection is rigorous, essential details of the genetic screening are missing, including screen library information, screening scale, and mutagenesis/coverage QC.

2. The manuscript shows that Cwf10 recruits Tgs1 during cryptic intron splicing-induced silencing. However, it remains unclear whether the other splicing factors identified in figure 2A and 2B play a role, given that Prp43 has a higher coverage than Cwf10. The authors can explain why Cwf10 is not identified in the initial genetic screening. In addition, the functional relationship between the novel TEaM machinery and the canonical splicing machinery remains unexplored.

3. Pir2-TMG interaction is not fully delineated (Figure 7). The manuscript demonstrates Tgs1-dependent Pir2 recruitment to gametogenic transcripts by RIP-seq. However, whether Pir2 directly interacts with TMG caps is not clear. The authors also mention that Pir2-CBC has a 7mG specificity, it would substantially strengthen the proposed mechanism if the preferential binding of Pir2 to TMG versus 7mG caps could be demonstrated.

4. Incomplete human analysis (Figure 8). While TGS1 knockdown in human BJ fibroblasts shows retrotransposon de-repression and antiviral activation, the manuscript does not investigate whether the mechanistic principles established in fission yeast apply to human. The unanswered questions include whether similar spliceosomal complex is identified, and whether the de-repressed human retrotransposons harbour cryptic introns. The authors may also consider utilizing more human cell lines to expand generalizability. Additionally, are gametogenic genes upregulated following TGS1 knockdown in human cells? On the other hand, considering this manuscript primarily focuses on yeast, these additional human cell data may not be essential.

5. In Extended data Fig 4d, the authors compare eas1Δ mug174-1 double mutant with tgs1Δ single mutant. The data would be improved if eas1Δ and mug174-1 single mutants (as in Figure 1B and 1C) can be included

6. The manuscript has several very short sections (e.g., "TEaM-mediated gene repression requires Tgs1 catalytic activity"). The authors can consider integrating these sections into adjacent sections for better narrative flow.

7. For all heatmaps comparing gene expression across conditions, adding violin plots would help illustrate whether expression differences between groups are statistically significant.
8. The analysis focuses on transposons and gametogenic genes. Are other genes also affected upon loss of the TEaM complex?

In summary, this work potentially reframes our understanding of substrate specificity in RNA silencing from DNA/RNA sequence motifs to RNA processing dynamics. The scope demonstrated in this study is broad and interdisciplinary, integrating chromatin regulation, RNA silencing, and splicing. The manuscript presents technical novelty and clear data presentation.

Version 1:

Reviewer comments:

Reviewer #1

(Remarks to the Author)
The manuscript was already excellent - the revisions have addressed all the remaining concerns.

Reviewer #2

(Remarks to the Author)
The authors have substantially revised the manuscript and added several new data that further strengthen their conclusions. In particular, they provide additional evidence that Tgs1 functions in the same pathway as Pir2 in the regulation of gametogenic transcripts. They also uncover important physiological roles of this pathway, demonstrating that Tgs1 suppresses meiotic transcripts and sporulation in the absence of the lncRNA sme2 and that Tgs1 is required for de novo siRNA production and heterochromatin re-establishment.

However, my principal concern regarding the conclusion that Tgs1, Mug174 and Eas1 assemble into a physical complex remains unresolved. I agree that several observations are consistent with this model, including the newly presented data showing that the in vivo association of Tgs1 with Mug174 and Eas1 depends on the presence of the respected partner proteins. Nevertheless, I remain unconvinced that the current evidence is sufficient to support the strong conclusion that these proteins form a trimeric complex, as stated in their response.

First, no direct biochemical evidence for such a complex is provided. Although the reported in vivo association are suggestive, biochemical purification remains the gold standard for establishing the composition and stoichiometry of a protein complex.

Second, I continue to find the genetic data difficult to reconcile with the proposed model. The observation that association of Mug174 with Tgs1 requires Eas1, and vice versa (Figure 2e and 2f), implies that disrupting both Mug174 and Eas1 should not produce strongly additive effects if their functions are exerted primarily through the same complex. The authors suggest that the missense mug174-1 allele may reveal functional information through genetic interactions; however, based on the co-IP data, the mug174-1 variant would not be expected to associate with Tgs1 in the absence of Eas1. Yet, as I noted in my previous review, the double mutant eas1Δ mug174-1 displays additive accumulation of ncRNAs, antisense and other transcripts. The authors acknowledge in the discussion that “Mug174 and Eas1 appear partially redundant in certain contexts”, but the regulation of these targets represents a central finding of this study. Consequently, it remains unclear how these observations support the strong conclusions regarding the proposed “TEaM complex” currently emphasized in the abstract and elsewhere in the text.

Taken together, the evidence supports a close functional and physical relationship among Tgs1, Mut174, and Eas1, but the data do not fully establish that these proteins form a physical complex. Given the absence of direct biochemical evidence and the apparent inconsistency between the interaction and genetic data, I strongly encourage the authors to moderate their claims and adopt more cautious wording throughout the manuscript.

Reviewer #3

(Remarks to the Author)
In the revised manuscript, the authors have adequately addressed most of the reviewers’ concerns, and the updated figures now reflect the statistical significance of key findings.
Minor points:
1. The new RIP-seq data extend the connection between TEaM and splicing regulation beyond Cwf10. Given that the MS data identify several other prominent splicing factors, the authors might consider briefly speculating in the Discussion about the possibility that additional splicing regulators could also contribute to TEaM recruitment.
2. The identification of additional TEaM-sensitive gene classes is highlighted in Supplementary Fig. 4d. A brief comment in Discussion to speculate whether these loci also tend to harbor cryptic introns would extend the TEaM-Pir2 pathway beyond the current retrotransposon focus.
3. The authors report upregulation of innate immune response genes and retrotransposon expression upon TGS1 knockdown in human cells. It would be valuable to discuss how these observations may relate to the previously identified dysregulated gametogenic transcripts. A brief elaboration in the Discussion on the potential mechanistic links could strengthen the conceptual framework of the study.

Open Access This Peer Review File is licensed under a Creative Commons Attribution 4.0 International License, which permits use, sharing, adaptation, distribution and reproduction in any medium or format, as long as you give appropriate credit to the original author(s) and the source, provide a link to the Creative Commons license, and indicate if changes were made.
In cases where reviewers are anonymous, credit should be given to 'Anonymous Referee' and the source.
The images or other third party material in this Peer Review File are included in the article's Creative Commons license, unless indicated otherwise in a credit line to the material. If material is not included in the article's Creative Commons license and your intended use is not permitted by statutory regulation or exceeds the permitted use, you will need to obtain permission directly from the copyright holder.
To view a copy of this license, visit https://creativecommons.org/licenses/by/4.0/

We thank the reviewers for their valuable feedback. Guided by their insightful comments and editorial recommendations, we have extensively revised the manuscript by performing additional experiments and updating the text accordingly. These revisions address all reviewer concerns and further strengthen the study's main conclusions.

Some of the key changes that we have made are as follows:

(a) An important finding of our study is that the trimethylguanosine synthase Tgs1/TEaM promotes the targeting of transcripts by the RNAi machinery through a mechanism involving Pir2/ARS2. In the revised manuscript, we present new data showing that Pir2/ARS2 associates with Pac1, the *S. pombe* homolog of the DROSHA ribonuclease (**Fig. 8a**). Our analyses further demonstrate that both Pir2/ARS2 and Pac1/DROSHA are required for the efficient production of initial siRNAs and for *de novo* heterochromatin assembly at centromeric repeats (**Fig. 8f, g and Supplementary Fig. 9b, c**). This finding is notable because mammalian ARS2 also associates with DROSHA during miRNA biogenesis. Together, these results suggest the existence of an evolutionarily conserved mechanism, analogous to the pathway involved in miRNA production, that initiates conversion of centromeric transcripts into siRNAs and triggers *de novo* heterochromatin nucleation by the RNAi machinery.

(b) Our analyses show that the Tgs1 RNA methyltransferase contributes to the silencing of meiotic genes in mitotically dividing cells; however, the biological consequences of their untimely expression in Tgs1-deficient cells had not been explored. To address this, we used a functional assay based on rescue of the meiotic arrest observed upon deletion of the *sme2* locus, which produces a non-coding RNA. Cells lacking *sme2* fail to undergo proper meiosis due to their inability to release RNA elimination factors from meiotic gene transcripts, thereby preventing their upregulation; this defect can be suppressed by deleting RNA elimination factors, leading to increased meiotic gene expression. We find that loss of Tgs1 similarly suppresses meiotic defects in *sme2Δ* cells, which correlates with derepression of meiotic genes (**Fig. 4g**). Indeed, deletion of *tgs1* in cells lacking the RNA elimination factor Pir1, which acts in parallel with Tgs1, rescued meiotic defects in 77.2% of cells, compared to 7.3% and 24.6% in *tgs1Δ* and *pir1Δ* single mutants, respectively, correlating with increased meiotic gene expression (**Fig. 4g**). Together, these results suggest that Tgs1-mediated silencing is functionally important for preventing inappropriate activation of the meiotic gene expression program.

(c) Based on mass spectrometry analyses of immunoaffinity-purified Mug174 and Eas1 proteins, we found that Tgs1, Mug174, and Eas1 co-purify, and these associations were independently confirmed by co-immunoprecipitation and western blot analyses. In response to a comment by reviewer 2 that additional evidence is required to support the conclusion that these proteins form a complex, we tested whether loss of Mug174 affects the association between Tgs1 and Eas1, and

conversely whether deletion of Eas1 alters the interaction between Tgs1 and Mug174. In both cases, Tgs1 interactions with Mug174 and Eas1 were disrupted in the absence of Eas1 and Mug174, respectively (**Fig. 2e, f**). Together with the co-purification data, these findings provide further support for the conclusion that Tgs1, Mug174, and Eas1 form a complex, although we cannot exclude the possibility that weaker dimeric interactions (Tgs1–Eas1 and Tgs1–Mug174) may exist or that Mug174 and Eas1 perform additional functions independently of the TEaM complex. These possibilities are now discussed in the revised text.

(d) We strengthened our conclusions regarding the epistatic relationship between Tgs1 and factors such as Ago1 and Pir2. As noted by Reviewer 2, epistatic interactions in weak mutants can only be meaningfully interpreted when a clear phenotype is present. To enable a more rigorous assessment, we have now performed our analyses under sensitized conditions, comparing the phenotype of the *tgs1Δ* mutant to other mutant alleles in a *pir1Δ* background, as recommended by the reviewer. For example, to establish the epistatic relationship between Tgs1 and Ago1 in meiotic gene silencing, we conducted RNA-seq and compared the levels of meiotic gene transcripts in *ago1Δ pir1Δ* and *tgs1Δ pir1Δ* double mutants, as well as in the *ago1Δ tgs1Δ pir1Δ* triple mutant (**Supplementary Fig. 6c, d**). We found that the triple mutant does not show cumulative upregulation beyond that observed in either double mutant, supporting the conclusion that these factors act in the same pathway. A similar analysis was performed for *pir2-1 tgs1Δ*, *tgs1Δ pir1Δ*, and *pir2-1 tgs1Δ pir1Δ* mutants (**Supplementary Fig. 8d, e**), yielding consistent results.

(e) We show that the Tgs1 RNA methyltransferase is recruited to target transcripts by the splicing machinery engaged at inefficiently spliced cryptic introns. Consistent with this, mutation of the splicing factor Cwf10 impairs Tgs1 recruitment to transcripts containing cryptic introns, including those produced from the *ura4* locus harboring a cryptic intron (*ura4-CI*). In response to a suggestion from Reviewer 3, we asked whether additional splicing factors might influence Tgs1 recruitment to *ura4-CI*. Because several subunits of the Prp19 complex co-purify with TEaM components, we examined a previously reported mutant allele of Prp5, a Prp19 complex subunit, to assess its role in Tgs1 recruitment and TMG capping of the *ura4-CI* transcripts. RNA immunoprecipitation experiments revealed that *prp5-1* mutant cells exhibit reduced Tgs1 recruitment, which correlates with decreased TMG capping of *ura4-CI* transcripts (**Fig. 2h, i**). Together, these findings indicate that, in addition to Cwf10, efficient targeting of Tgs1 to cryptic intron–containing transcripts requires a component of the Prp19 complex.

(f) Following the reviewer suggestion, we have added violin plots showing statistical significance for all mutant comparisons (**Supplementary Fig. 5b, c, d, g; Supplementary Fig. 6a, c; Supplementary Fig. 8b, d**).

(g) We have modified figures and substantially revised the text to incorporate all reviewer suggestions.

The specific comments made by the reviewers, which are indicated in italics, are addressed as follows (responses shown in blue):

Reviewer #1:

The reviewer commented, "*This is an impressive and expansive manuscript that describes a new complex of proteins, including tgs (and enzyme that makes trimethylated G caps on RNA) as critical for several aspects of gene silencing, splicing, siRNA production, retrotransposon silencing and other functions*.", and provided suggestions to improve the quality of our study.

We thank the reviewer for their encouraging remarks. We address the specific points below.

The question for me is the mechanism. It is appealing that the RNAs are targeted for trimethyl G capping which targets them for degradation. But the amount of TMG (trimethyl G) caps is not measured quantitatively, in part because there is no clear method to do so. But central to the idea is quantitative conversion of the caps of these mRNAs to TMG. It would be good if the authors can think of ways to address this.

The reviewer raises an important question and also acknowledges the methodological limitations that hinder quantitative measurement of TMG cap formation. Tgs1-target transcripts, including those derived from retrotransposons, centromeric repeats, and genic loci, are subject to degradation and are therefore generally present at much lower levels; this rapid turnover further complicates quantitative measurement of the TMG cap. However, in our study, we performed RNA immunoprecipitation using an antibody against the TMG cap, followed by RT-qPCR, to assay TMG capping of transcripts (**Fig. 1k, 2i**, **Supplementary Fig. 4f, Fig. 4j**). To ensure the validity of these results, we included appropriate controls. In addition to analyzing *tgs1Δ* strains, we also measured TMG capping in cells carrying a mutation in the catalytic methyltransferase domain of Tgs1 ($Tgs1^{G154D}$) (**Fig. 1k**). This mutation, which disrupts methyltransferase activity in vitro (**Fig. 1g**), also severely impairs TMG capping of Tgs1 target transcripts in vivo (**Fig. 1k**). Consistent with this defect, transcripts that are upregulated in *tgs1Δ* cells are similarly upregulated in the catalytic mutant, despite $Tgs1^{G154D}$ being expressed at levels comparable to wild-type Tgs1 (**Fig. 1j**). The concordance between the loss of TMG capping detected by the antibody-based assay and the derepression of Tgs1 target transcripts supports the validity of our conclusions (**Supplementary Fig. 4f, g; Fig. 4j, k**). While fully quantitative measurement of TMG caps would be valuable, such analyses require methodological advances beyond the scope of this 8-year study.

The other issue is that the Tgs enzyme also installs TMG caps on snRNAs, and the Tgs enzyme was found to interact with key snRNP components. How do we know that the effects on splicing are not due to impaired TMG

formation in the snRNA? I don't think this has been addressed and some evidence is needed to distinguish the contribution of different Tgs targets on the biological effects seen upon Tgs depletion.

We have considered the reviewer's suggestion. Although TMG is abundant on snRNAs required for pre-mRNA splicing, studies in *Drosophila* show that loss of Tgs1, which is essential for TMG capping, causes lethality without severely disrupting splicing (PMID: 33085660), and the TMG cap is not required for nuclear accumulation of snRNAs (PMID: 33085660). Moreover, in vitro reconstitution experiments have demonstrated that uncapped snRNAs can support various steps of splicing, indicating that the TMG cap is not an indispensable structural feature of the splicing machinery (PMID: 2524422; PMID: 7664740).

In this context, several lines of evidence argue that the phenotypes observed upon loss of Tgs1 in our study are unlikely to reflect general splicing defects. RNA-seq analyses show that Tgs1-deficient cells remain viable and exhibit no major global splicing abnormalities (**see Response Data Fig. 1➔**); instead, *tgs1* mutant alleles were repeatedly identified in our screen that show enhanced splicing of inefficient cryptic introns. In fact, our analyses demonstrated that increased splicing of cryptic introns is a general feature of *tgs1Δ* mutant cells. Importantly, we show that Tgs1 directly targets transcripts from retrotransposons, centromeric repeats, and meiotic genes, where it associates with these RNAs to install the TMG cap and promote recruitment of the Pir2/ARS2 complex, facilitating RNAi-mediated processing and silencing. Loss of TMG capping, including in catalytic Tgs1 mutants, correlates with defective silencing of these transcripts. Moreover, disruption of downstream factors such as Pir2 or Ago1 in cells with otherwise functional Tgs1 phenocopies the silencing defects seen in *tgs1*Δ cells. Collectively, these results support a direct role for Tgs1 in transcript silencing rather than suggesting an indirect effect via snRNA function.

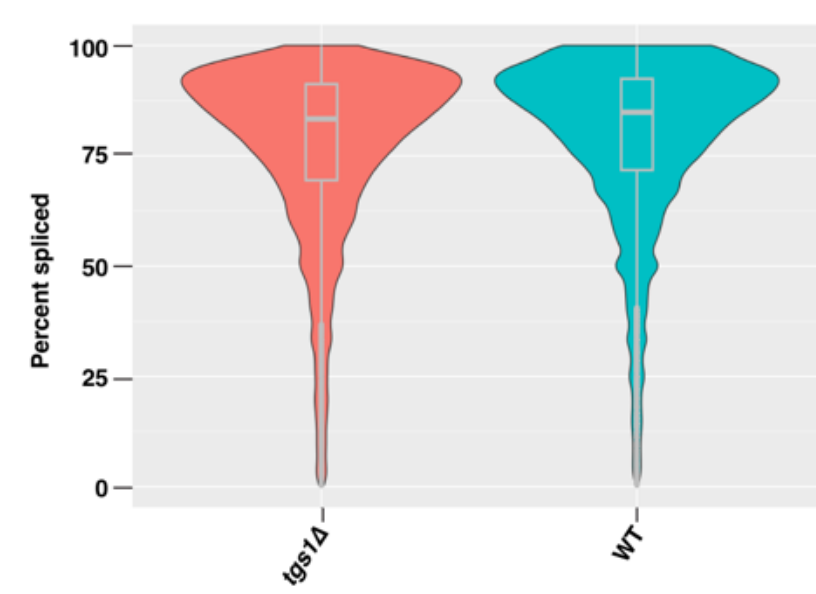

Response Data Fig.1 Distribution of splicing efficiencies for ~5100 canonical introns in RNA Seq of *tgs1Δ* (n = 3) and WT (n = 4). Efficiency is computed per intron from RNAseq alignments as the percentage of reads spanning the intron that are spliced at the intron boundaries. Median values are *tgs1Δ* = 83% and WT = 85%.

Reviewer #2:

The reviewer stated, "*Overall, this study provides new and important insights into the specificity and molecular mechanisms of RNA-targeting pathways that regulate non-coding RNAs, retrotransposons, and developmental genes through splicing-coupled and RNAi-mediated repression*" and noted " *the present work substantially advances the field by integrating these processes into a coherent network of regulatory pathways and by revealing functional connections that had not been fully appreciated. The experiments have been carefully performed, most data are appropriately represented, and the conclusions are, for the most part, well supported by the findings. In particular, the genetic data revealing redundancy among the different pathways are compelling*".

We sincerely appreciate the positive feedback and are thankful to the reviewer for insightful suggestions to improve the quality of our study. We have addressed the reviewer's concerns as follows.

The reviewer commented, "the genetic data revealing redundancy among the different pathways are compelling; however, *the physiological relevance of the Tgs1/Pir2-dependent RNAi pathway remains unclear, as its contribution is largely detectable only under sensitized conditions (i.e. in the absence of RNA degradation by MTREC-Pir1 or Swi6)*." We have performed additional experiments to address this important point.

First, to assess the physiological relevance of meiotic gene upregulation in cells lacking Tgs1, we employed a functional assay based on suppression of the meiotic arrest phenotype associated with deletion of the *sme2* locus, which encodes a noncoding RNA. Homothallic *sme2Δ* cells fail to undergo proper meiosis due to an inability to induce meiotic gene expression; however, this defect can be alleviated by disrupting RNA elimination factors that mediate meiotic gene silencing. We find that loss of Tgs1 similarly suppresses meiotic defects in *sme2Δ* cells, which tightly correlates with derepression of meiotic genes (**Fig. 4g**). Indeed, deletion of *tgs1* in cells lacking the RNA elimination factor Pir1, which acts in parallel with Tgs1, rescued meiotic defects in 77.2% of cells. Cells lacking Tgs1 alone also showed a marked increase in sporulation, with *sme2Δ tgs1Δ* double mutants exhibiting 7.3% sporulation compared to 0% in *sme2Δ* single mutant. Collectively, these findings indicate that Tgs1-mediated silencing of meiotic genes plays a critical functional role in preventing inappropriate activation of the meiotic gene expression program during mitotic growth. Regarding the comment that Tgs1's contribution is primarily detected under sensitized conditions, we note that redundancy in biological systems often necessitates the use of such conditions to reveal otherwise hidden functions of specific factors. In this context, our findings show that Tgs1/TEaM associates with Mmi1 to silence meiotic genes; however, this role has remained obscured due to redundancy with other Mmi1-associated silencing effectors.

Second, we provide evidence that both Tgs1 and Pir2 are required for the de novo generation of siRNAs at centromeres, a critical initiating step in the cascade underlying RNAi-mediated heterochromatin assembly. To investigate their involvement in siRNA production, we utilized cells lacking the H3K9 methyltransferase Clr4, which, as previously reported (PMID: 11283354, PMID: 15475954), are defective in siRNA generation (**Fig. 6d, 8g**). Reintroduction of functional *clr4⁺* restored efficient production of centromeric siRNAs in *clr4Δ* cells, as detected using Northern blot analyses (**Fig. 6d, 8g**). In contrast, siRNA production remained severely impaired when *clr4⁺* was introduced into *tgs1Δ clr4Δ* or *pir2-1 clr4Δ* mutant backgrounds (**Fig. 6d, 8g**). Our findings suggest that Tgs1, targeted through a mechanism involving cryptic introns and the splicing machinery, facilitates the recruitment of Pir2, which is in turn required for de novo siRNA production and the initiation of heterochromatin assembly at centromeres. In this context, our results delineate a mechanistic framework for the long-standing question of how siRNA production and heterochromatin formation are initially triggered.

Major points:

(1) Additional work is necessary to support the claim that Tgs1, Mug174 and Eas1 form a physical complex. At present, direct biochemical evidence is lacking, and several observations are difficult to reconcile in this model:
• The mutant mug174-1 (V32M) only partially recapitulates the splicing phenotypes of tgs1Δ and eas1Δ (Figure 1b, c) and is therefore not well suited for genetic interaction analysis.

Missense alleles have the potential to uncover functional information of proteins through genetic interaction analysis that might be missed in the deletion background. In the revised text, we note that the *mug174-1* allele maps to the Tgs1-interacting region of Mug174 (Deng et al., 2024; PMID: 38828770), which suggests that this interaction may be required for controlling cryptic intron splicing.

• The reported additive phenotypes of mug174-1 and eas1Δ (Figure 2, Ext. Figure 4-5), are inconclusive (see above) and are not readily consistent with a simple model of a stable ternary complex with Tgs1. Conversely, Mug174 condensates are affected differently in eas1Δ and tgs1Δ backgrounds, resulting in multiple foci.
• N-terminal residues (1-103) of Eas1 and Mug174 show high similarity based on structure prediction (Ext. Figure 2e). The N-terminal domain of Mg174 has previously shown to be necessary for interaction with Tgs1, suggesting that both Eas1 and Mu174 interact with Tgs1 via their N-terminal domains and may therefore compete for binding.
• Partially distinct sets of splicing factors co-purify with Mug174 and Eas1 (Figure 2a, b).
• Unlike tgs1Δ and eas1Δ, the complete deletion mutant mug174Δ displays a growth phenotype (not shown but reported in Deng 2024) and also displays quiescence phenotypes that differ from tgs1Δ (Deng 2024), suggesting additional functions,
--Given the weaker phenotype of eas1Δ relative to tgs1Δ, the additive phenotype with mug174-1, and the predicted structural similarity between Eas1 and Mug174, an alternative model is that Tgs1 forms two independent complexes with Eas1 and Mug174, respectively. Importantly, the presence of Mug174 in Eas1 pulldown/mass spectrometry experiments (and vice versa) does not by itself support a shared complex, as other factors unlikely to be part of such a complex (e.g. Mmi1, Sm proteins, and general splicing factors) are present at comparable levels. To demonstrate that all three proteins can co-exist in a shared complex, the authors should analyze purifications from yeast (or reconstituted recombinant proteins) using SDS page and size exclusion chromatography or density-gradient fractionation, as performed in their previous work (Thillainadesan et al 2020). Otherwise, the conclusion regarding a single physical 'TEaM' complex needs to be substantially toned down.

The reviewer raises several important points. Although Mug174, Tgs1, and Eas1 co-purify, they also display functional differences that are not uniformly shared among all three proteins. In particular, the reviewer notes that Mug174 foci are differentially affected in *tgs1Δ* and *eas1Δ* cells. In this context, it is important to consider that Mug174 foci predominantly correspond to snRNAs, as reported by Deng et al. (2024), and therefore may not reflect TEaM binding to other classes of transcripts. That said, loss of TEaM components produces similar phenotypic outcomes in other settings. Notably, our data show that deletion of *tgs1*, *eas1*, or *mug174*, when combined with *swi6Δ*, results in similar defects in centromeric

siRNA production, supporting the existence of a trimeric complex. However, we cannot exclude the possibility that individual components perform functions outside of the TEaM complex. For example, as the reviewer highlights, *mug174Δ* cells exhibit defects related to quiescence (Deng et al., 2024; PMID: 38828770). Such multifunctionality is not unusual, as many proteins act in multiple, context-dependent roles.

However, a key point raised by the reviewer is that Tgs1 might form two independent complexes with Mug174 and Eas1. We have addressed this point by performing additional experiments. Our co-immunoprecipitation experiments show that Tgs1–Mug174 interaction is severely affected in *eas1Δ* cells and likewise Tgs1–Eas1 interaction is compromised in *mug174Δ* cells. These results are now included in the paper (**see Fig. 2e, f**) and support the existence of a Mug174-Tgs1-Eas1 complex. However, we do not discount the existence of dimeric complexes of Tgs1-Eas1 and Tgs1-Mug174. As suggested by the reviewer, we have modified text to address these points (**e.g. see Discussion section**). We also attempted to perform glycerol gradient analyses, as recommended, but fractionating Mug174 that has been reported to form phase-separated condensates (Deng et al 2024 PMID: 38828770) proved technically challenging.

(2) *The authors argue that the absence of additive phenotypes between mutants indicates epistatic interactions and, by extension, that the corresponding factors act within the same pathway. However, expression analyses of selected Mmi1-regulated genes (Ext. Figures 5d, 6b, and 8b) lack comparison wild-type cells. This omission makes it difficult to assess whether the very low gene expression profiles observed in mutants such as tgs1Δ, pir2-1, or ago1Δ represent baseline expression or genuine mutant phenotypes. Epistatic interactions, particularly between weak mutants, can only be meaningful interpreted when a clear phenotype is present. Otherwise, the absence of phenotypes in single and double mutants merely indicate that neither factor contributes clearly under the tested conditions. For a rigorous assessment of epistatic interactions, the authors should analyze these mutants under sensitized conditions where phenotypes are robust, for example by combining them with pir1Δ and comparing the double mutants pir1Δ tgs1Δ and pir1Δ ago1Δ to the corresponding triple mutant pir1Δ tgs1Δ ago1Δ. I would also strongly recommend including control genes or loci that are not affected by these pathways to allow a clearer assessment of specificity.*

We thank the reviewer for making this important point. In response, we have strengthened our conclusions regarding the epistatic relationships by performing additional experiments under more rigorous, sensitized conditions. Specifically, we re-examined the phenotype of the *tgs1Δ* mutant in comparison with other mutant alleles in a *pir1Δ* background, as recommended. To this end, we generated new RNA-seq datasets assessing the levels of gametogenic transcripts in *tgs1Δ pir1Δ*, *ago1Δ pir1Δ*, and *ago1Δ tgs1Δ pir1Δ* triple mutants (**Supplementary Fig. 6c, d**), and compared transcript levels in *pir2-1 pir1Δ*, *tgs1Δ pir1Δ*, and *pir2-1 tgs1Δ pir1Δ* triple mutants (**Supplementary Fig. 8d, e**). In both cases, the triple mutants do not exhibit cumulative upregulation beyond that observed in the corresponding double mutants, consistent with these factors acting within the same pathway. As suggested by the reviewer, we have also included a control locus that remains unaffected by these

pathways. Finally, to further examine the epistatic relationship between Tgs1 and Ccr4, an interaction less extensively discussed in the manuscript relative to Ago1 and Pir2, we performed RT–qPCR analysis in the *ccr4Δ tgs1Δ pir1Δ* triple mutant, alongside the *tgs1Δ pir1Δ* and *ccr4Δ pir1Δ* double mutants; these data, showing epistatic relationships, are now included in **Supplementary Fig. 5f** (right panel).

(3) *Several results reported here confirm key findings from previous studies, but these are not sufficiently acknowledged or integrated. For example, the authors report that pericentromeric transcripts are TMG-capped (Figure 6d), that Tgs1 activity contributes to heterochromatin silencing (Figure 6f), that Tgs1 is required for H3K9me maintenance (Figure 6c) and Pir2-1 required for de novo heterochromatin assembly (Ext. Figure 8g), and that Tgs1 acts in parallel to Swi6 in RNAi-dependent heterochromatin silencing at pericentromeres (Figure 6b, c, f). With the exception of Pir2-1, (which largely phenocopies tgs1Δ), these findings were already reported by Yu et al. (2020; PMID: 33527595). Similarly, Deng et al. (2024; PMID: 38828770) demonstrated that Mug174 contributes to pericentromeric heterochromatin assembly. I strongly recommend combining these largely confirmatory data in the supplementary material and reframing them explicitly in the context of the existing literature. Deng et al 20204 further showed that Mug174 interacts and colocalize with Tgs1, that Mug174 foci formation depend on Tgs1, and that snRNA TMG capping requires Mug174, analogous to Tgs1. Although the physical association and functional relationship between Tgs1 and Mug174 were established prior to the present study, this information is marginally discussed in the current manuscript and should be incorporated more explicitly.*

We have revised the text to include additional citations of relevant literature. The papers noted by the reviewer, including Deng et al. (2024 PMID: 38828770), were already cited; however, we have now incorporated further citations to these studies where appropriate. In response to the suggestion to move certain datasets to supplementary material, we have relocated some results, such as the TMG capping of centromeric transcripts, to the Supplementary Figures. However, we have retained in the main figure the data demonstrating that Tgs1 acts in parallel with Swi6 to promote centromeric siRNA production. These data serve as an important control alongside our results showing the effects of *mug174Δ* and *eas1Δ* on siRNA production in the presence and absence of Swi6 (**Fig. 6b, c**). Importantly, these experiments establish that the TEaM complex, rather than Tgs1 alone, is required for Swi6-independent generation of centromeric siRNAs. This information is central to the conclusions of our study. These results, together with data demonstrating that Tgs1 is essential for de novo generation of centromeric siRNAs, provide important insights into the initiating steps of RNAi-mediated heterochromatin assembly.

With respect to the results involving Pir2, we now provide additional evidence that Pir2/ARS2 associates with Pac1, the *S. pombe* homolog of the DROSHA ribonuclease, and further show that like Pir2/ARS2, Pac1 is required for retrotransposon repression. Additionally, we find that both Pac1 and Pir2/ARS2 are required for efficient production of initial siRNAs and for de novo heterochromatin assembly at centromeric repeats. These findings are presented in the new **Fig. 8** and **Supplementary Fig. 9**. Notably, mammalian ARS2 has been reported to associate with DROSHA during miRNA biogenesis, and our results therefore suggest the existence of an analogous mechanism that initiates the primary cascade

converting centromeric transcripts into siRNAs. Taken together, these analyses support a model in which the TEaM complex facilitates Pir2 recruitment, which in turn cooperates with Pac1 and other factors involved in Swi6-independent siRNA production to trigger de novo heterochromatin nucleation by the RNAi machinery.

Minor points

Text, page 9: "Similar cumulative derepression was observed when tgs1Δ was combined with deletion of ssu72" appears overstated. Although transcript levels in the double mutant are modestly increased relative to the single mutants, the cumulative derepression (~ 2-3 rpm) is substantially weaker than that observed in tgs1Δ pir1Δ (10-60 rpm). Please consider revising this statement accordingly.

We have made the suggested changes in the text.

Extended Figure S4 a, b vs. Figure 3a, b: Please clarify whether experiments in Extended Figure 4 a, b were also performed in an abp1 mutant background, as in Figure 3 a, b. This not clear from Results text. More generally, I recommend indicating sensitized strain backgrounds not only in the legend (as currently done) but also directly in the Figures (Figures 3a/Ext. Figure 4a), either in the panels or axis label (e.g. "Tgs1 enrichment in pir1Δ/abp1-DDE strain").

We have revised the figures to include the relevant strain background along the axis.

Suppl Figure S3c: mug174-1 should be labeled as mug174-1-GFP (panel 4)

We thank the reviewer for pointing this out and have corrected the label.

Suppl. Figure S5h: Please clarify whether expression data in the right column were obtained in tgs1Δ or tgs1Δ mmi1Δ double mutants.

Yes, the expression data presented were obtained using single *tgs1Δ* mutant cells.

mmi1 mutant: According to S. pombe literature, mmi1 is an essential gene, but its essential function can be bypassed by deleting mei2. Please clarify how mmi1Δ experiments have been performed and in which genetic background.

Indeed, as noted by the reviewer, *mmi1* is an essential gene, but its essential function can be bypassed by a deletion in the *mei4* locus. Accordingly, all experiments were performed using an *mmi1Δ* strain carrying a truncation of *mei4* (*mei4ΔC*). This information has now been included in the Methods section and in the figure legends.

Reviewer #3 (Remarks to the Author):

The reviewer states "*In summary, this work potentially reframes our understanding of substrate specificity in RNA silencing from DNA/RNA sequence motifs to RNA processing dynamics. The scope demonstrated in this*

study is broad and interdisciplinary, integrating chromatin regulation, RNA silencing, and splicing. The manuscript presents technical novelty and clear data presentation".

We thank the reviewer for these positive remarks and for helpful suggestions. We have addressed all concerns as follows.

Specific comments:

1. Missing genetic screening details. Although the design of the forward genetic screen with backcross selection is rigorous, essential details of the genetic screening are missing, including screen library information, screening scale, and mutagenesis/coverage QC.

We thank the reviewer for pointing this out. We have now included the genetic screen details such as number of reads, read depth and coverage in the methods section.

2. *The manuscript shows that Cwf10 recruits Tgs1 during cryptic intron splicing-induced silencing. However, it remains unclear whether the other splicing factors identified in figure 2A and 2B play a role, given that Prp43 has a higher coverage than Cwf10. The authors can explain why Cwf10 is not identified in the initial genetic screening. In addition, the functional relationship between the novel TEaM machinery and the canonical splicing machinery remains unexplored.*

We show that the splicing factor Cwf10 is required for the recruitment of Tgs1 to transcripts harboring inefficiently spliced cryptic introns (**Fig. 2h, i,** and **3a, b**). In the absence of Tgs1, splicing of these cryptic introns is enhanced, as observed in our genetic screen using a *ura4* reporter containing a cryptic intron (**Fig. 1c**). The reviewer raised the question of why *cwf10* was not identified in this screen. A plausible explanation is that, in addition to its role in recruiting Tgs1, Cwf10 is itself required for the splicing of cryptic introns. Indeed, our prior work has shown that efficient splicing of the cryptic intron within *tf2* retrotransposons depends on Cwf10 (Lee et al., 2013; PMID: 24210919).

Our selection of Cwf10 for this study was guided by two primary considerations. First, our recent work established a requirement for Cwf10 in de novo siRNA synthesis from transcripts harboring cryptic introns (Srivastav et al., 2025; PMID: 39747188). Second, Cwf10 was previously implicated in RNAi-mediated heterochromatin formation at centromeric repeats (Bayne et al., 2008; PMID: 18948543), which generate transcripts containing cryptic introns (Lee et al., 2013; PMID: 24210919). Notably, this function appears to be restricted to a subset of splicing factors rather than a general property of the spliceosome. In any case, considering that multiple subunits of the Prp19 splicing complex were identified in both Mug174 and Eas1 purified fractions (**Supplementary Data Table 1**), we investigated whether this complex contributes to Tgs1 recruitment to transcripts containing cryptic introns. To address this, we performed RNA immunoprecipitation assays using the available mutant allele *prp5-1*, a component of the Prp19 complex, to assess Tgs1 targeting and TMG capping of *ura4-CI* transcripts. Similar to *cwf10-1* cells,

the *prp5-1* mutant exhibited reduced Tgs1 recruitment and diminished TMG capping of *ura4*-CI transcripts (**Fig. 2h, i**). These data have now been incorporated into the revised manuscript. Due to the lack of available mutant alleles for the essential *prp43* gene, we were unable to evaluate its potential role in Tgs1 recruitment.

3. *Pir2-TMG interaction is not fully delineated (Figure 7). The manuscript demonstrates Tgs1-dependent Pir2 recruitment to gametogenic transcripts by RIP-seq. However, whether Pir2 directly interacts with TMG caps is not clear. The authors also mention that Pir2-CBC has a 7mG specificity, it would substantially strengthen the proposed mechanism if the preferential binding of Pir2 to TMG versus 7mG caps could be demonstrated.*

We appreciate this point made by the reviewer. Indeed, it would be interesting to know how TMG mediates recruitment of Pir2/ARS2 protein. Our current hypothesis is that Pir2/ARS2 association with the TMG cap is mediated indirectly through one or more bridging protein factors. However, despite extensive efforts, we have not yet identified the factor(s) responsible for linking Pir2/ARS2 to the TMG cap. Notably, our attempts to address this question led to the unexpected finding that Pir2/ARS2 associates with the *S. pombe* homolog of DROSHA, Pac1 (**Fig. 8a**). Furthermore, we show that Pac1, similar to Tgs1 and Pir2/ARS2, functions not only in meiotic gene silencing and retrotransposon repression (**Fig. 8b–d, Supplementary Fig. 9a**) but is also required for de novo siRNA production and heterochromatin establishment at centromeric repeats (**Fig. 8f, g, Supplementary Fig. 9b, c**).

4. *Incomplete human analysis (Figure 8). While TGS1 knockdown in human BJ fibroblasts shows retrotransposon de-repression and antiviral activation, the manuscript does not investigate whether the mechanistic principles established in fission yeast apply to human. The unanswered questions include whether similar spliceosomal complex is identified, and whether the de-repressed human retrotransposons harbour cryptic introns. The authors may also consider utilizing more human cell lines to expand generalizability. Additionally, are gametogenic genes upregulated following TGS1 knockdown in human cells? On the other hand, considering this manuscript primarily focuses on yeast, these additional human cell data may not be essential.*

We agree with the reviewer that the primary focus of this study is fission yeast. In response to this suggestion, we have moved the human data to **Supplementary Figure 10** and removed any mention of these analyses from the abstract. We have also taken care to avoid overinterpreting the results from the human data.

5. *In Supplementary Fig 4d, the authors compare eas1Δ mug174-1 double mutant with tgs1Δ single mutant. The data would be improved if eas1Δ and mug174-1 single mutants (as in Figure 1B and 1C) can be included.*

The single mutants *eas1Δ* and *mug174-1* are included in **Supplementary Fig. 4d**

6. *The manuscript has several very short sections (e.g., "TEaM-mediated gene repression requires Tgs1 catalytic activity"). The authors can consider integrating these sections into adjacent sections for better narrative flow.*

As suggested, we have consolidated some of the smaller sections in the revised version.

7. *For all heatmaps comparing gene expression across conditions, adding violin plots would help illustrate whether expression differences between groups are statistically significant.*

We thank the reviewer for this excellent suggestion. We have now added violin plots with P values for the different mutant combinations presented.

8. *The analysis focuses on transposons and gametogenic genes. Are other genes also affected upon loss of the TEaM complex?*

Some other categories of genes that are derepressed upon loss of the TEaM complex are non-coding RNA, antisense transcripts, stress response genes and wtf elements. These categories are highlighted in **Supplementary Fig. 4d.**

Response to Reviewer Comments:

We sincerely appreciate the reviewers' thoughtful and constructive feedback and thank you for your assistance in the review process. The reviewers' remaining comments, presented in *italics*, are addressed below, with our responses shown in blue.

Reviewer #1

The manuscript was already excellent - the revisions have addressed all the remaining concerns.

We thank the reviewer for supporting the publication of our work.

Reviewer #2

The authors have substantially revised the manuscript and added several new data that further strengthen their conclusions. In particular, they provide additional evidence that Tgs1 functions in the same pathway as Pir2 in the regulation of gametogenic transcripts. They also uncover important physiological roles of this pathway, demonstrating that Tgs1 suppresses meiotic transcripts and sporulation in the absence of the lncRNA sme2 and that Tgs1 is required for de novo siRNA production and heterochromatin re-establishment."

We sincerely thank the reviewer for their suggestions and appreciate their positive assessment that the newly added results have substantially strengthened the manuscript.

However, my principal concern regarding the conclusion that Tgs1, Mug174 and Eas1 assemble into a physical complex remains unresolved. I agree that several observations are consistent with this model, including the newly presented data showing that the in vivo association of Tgs1 with Mug174 and Eas1 depends on the presence of the respected partner proteins. Nevertheless, I remain unconvinced that the current evidence is sufficient to support the strong conclusion that these proteins form a trimeric complex, as stated in their response.

First, no direct biochemical evidence for such a complex is provided. Although the reported in vivo association are suggestive, biochemical purification remains the gold standard for establishing the composition and stoichiometry of a protein complex.

Second, I continue to find the genetic data difficult to reconcile with the proposed model. The observation that association of Mug174 with Tgs1 requires Eas1, and vice versa (Figure 2e and 2f), implies that disrupting both Mug174 and Eas1 should not produce strongly additive effects if their functions are exerted primarily through the same complex. The authors suggest that the missense mug174-1 allele may reveal functional information through genetic interactions; however, based on the co-IP data, the mug174-1 variant would not be expected to associate with Tgs1 in the absence of Eas1. Yet, as I noted in my previous review, the double mutant eas1Δ mug174-1 displays additive accumulation of ncRNAs, antisense and other transcripts. The authors acknowledge in the discussion that "Mug174 and Eas1 appear partially redundant in certain contexts", but the regulation of these targets represents a central finding of this study. Consequently, it remains unclear how these observations support the strong conclusions regarding the proposed "TEaM complex" currently emphasized in the abstract and elsewhere in the text.

Taken together, the evidence supports a close functional and physical relationship among Tgs1, Mut174, and Eas1, but the data do not fully establish that these proteins form a physical complex. Given the absence of direct biochemical evidence and the apparent inconsistency between the interaction and genetic data, I strongly encourage the authors to moderate their claims and adopt more cautious wording throughout the manuscript.

The reviewer agrees that our data support "*a close functional and physical relationship among the Tgs1, Mug174, and Eas1*", but points to the results showing that deletion of *eas1*, when combined with the partial loss-of-function *mug174-1* mutant, produces an additive phenotype. In response to the reviewer's comments, we have further revised the text to address this concern and acknowledge alternative interpretations. However, we emphasize that our conclusion that Tgs1, Mug174, and Eas1 are closely associated both physically and functionally is supported by multiple independent lines of evidence, as summarized below.

(1) Our biochemical analyses demonstrate that Mug174, Tgs1, and Eas1 co-purify with each other. Indeed, mass spectrometry analysis of Mug174 affinity-purified fractions identified both Tgs1 and Eas1. Reciprocally, affinity purification of Eas1 recovered both Tgs1 and Mug174 (see Fig. 2). These interactions were independently validated by co-immunoprecipitation and western blot analyses. In particular, affinity purification of GFP-tagged Tgs1 resulted in the co-purification of both Mug174 and Eas1.

(2) We further show that the interactions among Mug174, Tgs1 and Eas1 proteins are interdependent, consistent with their assembly into a common complex. Specifically, we tested whether loss of Mug174 affects the Tgs1–Eas1 interaction and whether deletion of Eas1 disrupts the Tgs1–Mug174 interaction. In both cases, association of Tgs1 with its partner was abolished in the absence of the third component (Fig. 2e,f).

(3) Beyond the biochemical evidence, our functional analyses strongly support the conclusion that Mug174, Tgs1 and Eas1 act together as a complex. Loss of Mug174, Tgs1, or Eas1 produces highly similar phenotypic outcomes across multiple assays. Notably, deletion of *mug174*, *tgs1*, or ***eas1***, when combined with *swi6Δ*, results in comparable defects in centromeric siRNA production and histone H3K9 methylation (Fig. 6b).

(4) Consistent with these findings, all three proteins were repeatedly identified in our genetic screen for factors required for cryptic intron splicing. Indeed, mutations in *mug174*, *tgs1*, and *eas1* similarly affect splicing of a cryptic intron inserted into both *ura4* (Fig. 1 and Supplementary Fig. 1) and *ade6* (Fig. 1f) reporter genes.

Taken together, our biochemical purification and functional analyses strongly support a close physical and functional association among Tgs1, Mug174, and Eas1. We appreciate the reviewer's comment regarding the precise stoichiometry of the complex. In response, we have revised the manuscript to avoid making assertions about stoichiometry, explicitly acknowledge the limitations of our data, and discuss alternative interpretations. **For example, we now describe TEaM as a protein assembly containing Tgs1, Mug174, and Eas1, rather than referring to it as a "trimeric" complex.**

We also explicitly state that **"we do not discount the possibility that residual association between Tgs1 and Mug174 is maintained in cells lacking Eas1, and vice versa."** In addition, in the section describing transcript upregulation in the *eas1Δ mug174-1* double mutant, we now state that **"The synthetic enhancement observed in the *mug174-1 eas1Δ* double mutant is consistent with a model in which Mug174 and Eas1 cooperatively support Tgs1-dependent TEaM activity. Under this model, the hypomorphic *mug174-1* allele retains sufficient function to sustain TEaM activity in cells expressing functional Eas1, but not when Eas1 is simultaneously absent."** Accordingly, the genetic interaction between *mug174-1* and *eas1Δ* is consistent with synthetic enhancement between a hypomorphic allele and a cooperating component of the same molecular pathway or protein assembly. Such interactions have

been widely exploited in other systems, including *C. elegans* (e.g. see PMID: 22118276), to identify functionally related proteins within the same pathway or complex.

Beyond these revisions, we have adopted more cautious language throughout the manuscript when referring to TEaM. Furthermore, the Discussion explicitly states that "unlike *tgs1Δ* and *eas1Δ* mutants, cells lacking Mug174 display growth defects, and Mug174 and Eas1 appear partially redundant in certain contexts. These differences suggest that, in addition to their shared role within TEaM, the individual components likely perform distinct functions outside this assembly."

We believe that these revisions appropriately address the reviewer's concerns while preserving conclusions that are fully supported by the biochemical and genetic evidence.

Reviewer #3

In the revised manuscript, the authors have adequately addressed most of the reviewers' concerns, and the updated figures now reflect the statistical significance of key findings.

We thank the reviewer for these positive remarks and for their helpful suggestions.

Minor points:

The new RIP-seq data extend the connection between TEaM and splicing regulation beyond Cwf10. Given that the MS data identify several other prominent splicing factors, the authors might consider briefly speculating in the Discussion about the possibility that additional splicing regulators could also contribute to TEaM recruitment.

In response to this point, we have added the following sentence in the discussion **"Both Cwf10 and Prp5, which we show are required for Tgs1 association and TMG capping of transcripts containing cryptic introns, have previously been implicated in siRNA production and RNAi-mediated heterochromatin formation at centromeric repeats. Importantly, this function appears to be restricted to a subset of splicing factors rather than representing a general property of the spliceosome"**.

The identification of additional TEaM-sensitive gene classes is highlighted in Supplementary Fig. 4d. A brief comment in Discussion to speculate whether these loci also tend to harbor cryptic introns would extend the TEaM-Pir2 pathway beyond the current retrotransposon focus.

Accordingly, we have added this statement in the discussion **"Our finding that 45% of transcripts upregulated in *tgs1Δ* cells contain cryptic introns, implies a broader role of Tgs1 in cryptic intron mediated gene regulation."**.

The authors report upregulation of innate immune response genes and retrotransposon expression upon TGS1 knockdown in human cells. It would be valuable to discuss how these observations may relate to the previously identified dysregulated gametogenic transcripts. A brief elaboration in the Discussion on the potential mechanistic links could strengthen the conceptual framework of the study.

We have included the following statement in the discussion section. "**In human cells, TGS1 depletion results in the accumulation of retrotransposon transcripts and activation of antiviral responses, raising the possibility that TGS1 cooperates with RNA-processing factors involved in silencing repetitive elements and gametogenic genes, such as ERH. Unlike in *S. pombe*, however, TGS1 knockdown did not induce gametogenic gene expression in BJ fibroblasts, likely reflecting the low basal expression of these genes in this cell type. Future studies in appropriate developmental and disease models will be**

required to determine whether TGS1 similarly suppresses inappropriate activation of gametogenic genes in mammals, including cancer-testis antigens."
